# A Two-Tier Golgi-Based Control of Organelle Size Underpins the Functional Plasticity of Endothelial Cells

**DOI:** 10.1016/j.devcel.2014.03.021

**Published:** 2014-05-12

**Authors:** Francesco Ferraro, Janos Kriston-Vizi, Daniel J. Metcalf, Belen Martin-Martin, Jamie Freeman, Jemima J. Burden, David Westmoreland, Clare E. Dyer, Alex E. Knight, Robin Ketteler, Daniel F. Cutler

**Affiliations:** 1 Endothelial Cell Biology Laboratory, Laboratory for Molecular and Cellular Biology, University College London, Gower Street, London WC1E 6BT, UK; 2 Translational Research Resource Center, Laboratory for Molecular and Cellular Biology, University College London, Gower Street, London WC1E 6BT, UK; 3 Bioinformatics Image Core, Laboratory for Molecular and Cellular Biology, University College London, Gower Street, London WC1E 6BT, UK; 4 Analytical Science Division, National Physical Laboratory, Hampton Road, Teddington, Middlesex TW11 0LW, UK; 5 Electron Microscopy Laboratory, Laboratory for Molecular and Cellular Biology, University College London, Gower Street, London WC1E 6BT, UK

## Abstract

Weibel-Palade bodies (WPBs), endothelial-specific secretory granules that are central to primary hemostasis and inflammation, occur in dimensions ranging between 0.5 and 5 μm. How their size is determined and whether it has a functional relevance are at present unknown. Here, we provide evidence for a dual role of the Golgi apparatus in controlling the size of these secretory carriers. At the ministack level, cisternae constrain the size of nanostructures (“quanta”) of von Willebrand factor (vWF), the main WPB cargo. The ribbon architecture of the Golgi then allows copackaging of a variable number of vWF quanta within the continuous lumen of the *trans*-Golgi network, thereby generating organelles of different sizes. Reducing the WPB size abates endothelial cell hemostatic function by drastically diminishing platelet recruitment, but, strikingly, the inflammatory response (the endothelial capacity to engage leukocytes) is unaltered. Size can thus confer functional plasticity to an organelle by differentially affecting its activities.

## Highlights

• Cisternal length within Golgi ministacks controls the size of vWF cargo nanostructures
• The Golgi ribbon allows copackaging of vWF nanostructures into WPBs of variable size
• Endothelial cells with small WPBs display a reduced platelet recruitment capability
• Control of organelle size may confer hemostatic plasticity to endothelia

## Graphical Abstract

**Figure:**
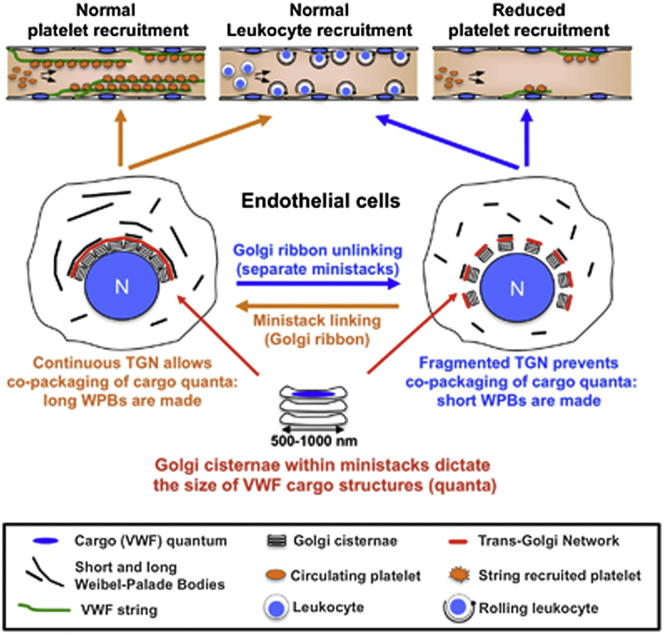

## Teaser

Weibel-Palade bodies are secretory granules central to the initiation of endothelial-mediated hemostasis and inflammation. Ferraro et al. show that Golgi ribbon architecture is required for assembly of large WPBs from cisterna-molded quanta of vWF cargo. Organelle size changes affect recruitment of platelets, but not leukocytes, conferring functional plasticity to endothelia.

## Introduction

**Figure 1:**
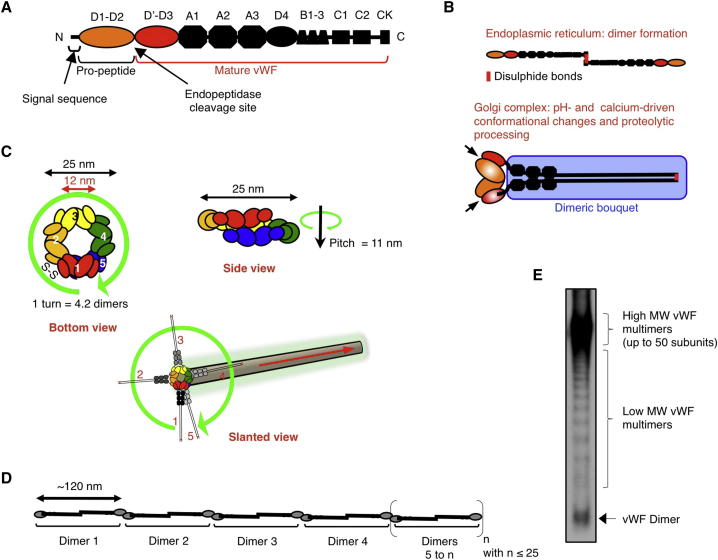
Biosynthesis and Structural Features of vWF (A) Diagram of the domain composition of prepro-vWF (Metcalf et al., 2008; Sadler, 1998). (B) Disulfide bonds formation in the ER generates pro-vWF dimers, and in the Golgi lumen, acidic pH and calcium promote the “dimeric bouquet” (blue boxed region) conformation (Zhou et al., 2011). Golgi’s acidic milieu is also required for proteolytic processing (arrows), likely by furin, which generates the prodomain and mature vWF. (C) At the Golgi, calcium and low pH promote vWF tubulation. A diagram of the spatial arrangement of five dimers (numbered 1–5) assembled into a tubule is shown. The propeptide (D1-D2) and the D’-D3 domains of each dimer are arranged into a right-hand helix forming the wall of the tubule (Huang et al., 2008). The helix has a period of 4.2 dimers and a pitch of 11 nm (for clarity, domains A1-CK are omitted in “bottom” and “side” views). In tubules, the configuration of D’-D3 domains of adjacent dimers is favorable to interdimer disulfide bond formation (S-S in bottom view) required for vWF multimerization. (D) Upon exocytosis, the shift to neutral pH disrupts propeptide/D’-D3 interactions and the bouquet conformation, leading to the extension of multimerized vWF. (E) vWF multimer analysis of an endothelial cell fraction containing WPBs (see Supplemental Experimental Procedures and Figure S1G). From bottom to top, bands visualize dimers, tetramers, hexamers, etc. In WPBs, the high-molecular-weight multimers contribute the majority of multimerized vWF.

Discovered five decades ago (Weibel and Palade, 1964), Weibel-Palade bodies (WPBs) are endothelial-specific secretory granules that are fundamental to the initiation of hemostatic and inflammatory responses. WPBs store endothelial von Willebrand factor (vWF), a large glycoprotein that undergoes complex processing along the secretory pathway (Metcalf et al., 2008). Synthesized as a proprotein, vWF dimerizes in the ER and is proteolytically cleaved in the Golgi apparatus, generating the propeptide and mature vWF forms, which remain tightly associated (Figures 1A–1C). The acidic milieu of the Golgi lumen is needed for cleavage, conformational changes, and self-assembly (Huang et al., 2008; Zhou et al., 2011). These processes are required for VWF’s compact packaging into tubules visible by electron microscopy (EM) (Weibel and Palade, 1964; Zenner et al., 2007), as well as its multimerization via extensive interchain disulfide bonding between mature dimers (Mayadas and Wagner, 1989; Figures 1C–1E). At the *trans*-Golgi network (TGN), vWF is finally packaged into nascent WPBs, which are 200–300 nm wide and have lengths in the micrometer range, through a process requiring the cytosolic clathrin/AP-1 coat machinery (Lui-Roberts et al., 2005; Metcalf et al., 2008).

vWF is fundamental to primary hemostasis. Upon vessel injury, WPBs undergo exocytosis, and the coiled vWF multimers, with masses reaching 20 MDa (Figures 1D and 1E), unfurl in the direction of blood flow, ultimately forming strings that can extend hundreds of micrometers. These strings bind circulating platelets, promoting their aggregation into a loose plug, the first step to curb hemorrhage. Quantitative and/or qualitative deficiencies in secreted vWF multimers lead to the most common bleeding disorder in humans, von Willebrand’s disease, which is estimated to affect up to 1% of the population (Sadler, 1998), while elevated serum vWF levels are associated with cardiovascular pathologies (van Galen et al., 2012). vWF is also involved in angiogenesis and inflammation and has been linked to atherosclerosis (Methia et al., 2001; Petri et al., 2010; Starke et al., 2011). We previously established that the typical elongated shape of WPBs reflects the lumenal arrangement of vWF tubules and is optimal for the organelle’s hemostatic role (Michaux et al., 2006a). However, how WPBs of vastly different sizes are generated and whether this is reflected in their physiology remain unknown. Here, we addressed these questions.

## Results

### A Length Unit for WPBs

**Figure 2:**
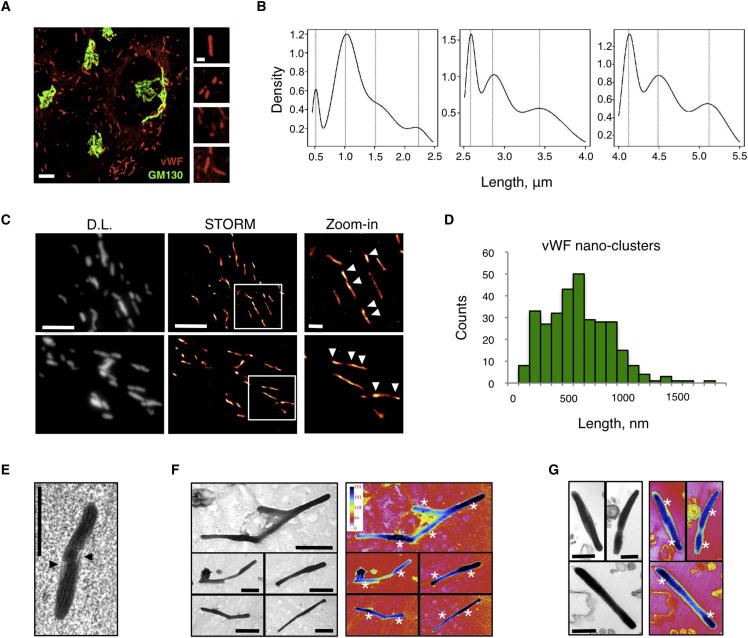
A Length Unit for WPBs (A) WPBs (vWF) and the Golgi (GM130) were visualized in HUVECs; scale bar, 5 μm. Right: magnified regions exemplify WPB length variability; scale bar, 1 μm. (B) High-throughput microscopic survey; the lengths of ∼2 million WPBs were measured and modeled to a mixture of Gaussian distributions (see Supplemental Experimental Procedures). (C) Diffraction-limited (DL) images of vWF-labeled HUVECs and their STORM reconstructions. Scale bar, 2 μm; zoom, 1 μm. Arrowheads indicate vWF nanoclusters within WPBs. (D) vWF nanocluster size frequency was quantified from STORM images; n = 312; median [quartiles] = 576 [382, 797] nm. (E) Electron micrograph from an HPF/FS HUVEC sample of a WPB (membrane continuities, arrowheads) showing two distinct regions. (F and G) TEM of cell-free WPBs chemically fixed and prepared for whole-mount (F) or thick sections (G). Gray levels were color-coded to highlight variation in content density (denser regions labeled by asterisks). Scale bars in (E)–(G), 500 nm. See also Figure S1 and Table S1.

WPBs are present in different sizes ranging between 0.5 and 5 μm (Weibel and Palade, 1964). To gain insight into possible mechanisms of size acquisition, we carried out a high-throughput microscopic survey on cultured human umbilical vein endothelial cells (HUVECs) stained for vWF to label these organelles. Due to their elongated shape (Figure 2A), changes in WPB size could be expressed as organelle length, an easily quantifiable parameter (see Supplemental Experimental Procedures and Figures S1A–S1C available online). Under our culture conditions, long WPBs represented a minority of the total population but contained a significant amount of vWF (Figure S1D, “number” versus “area”), a possible indication that long WPBs might be physiologically more important than suggested by their number. Most strikingly, our survey uncovered that WPB lengths cluster around values regularly spaced at ∼0.5 μm intervals (Figures 2B and S1E; Table S1). The periodic occurrence of “preferred” organelle lengths suggested the existence of a “length unit” for WPBs, with the number of units determining the size of individual organelles.

Since vWF expression is necessary for the formation of WPBs (Denis et al., 1998; Wagner et al., 1991) (see Figures 5A and 5B), we tested whether it also accounted for the “unit” identified in our morphometric survey. Nanoscopic imaging of vWF immunoreactivity in HUVECs by stochastic optical reconstruction microscopy (STORM), a superresolution technique (Fölling et al., 2008; Rust et al., 2006), showed that this cargo displayed localization clusters discretely spaced along the length of each organelle (Figures 2C, arrowheads, and S1F). These localization clusters were also observed using a monoclonal antibody to vWF (data not shown) and were not artifacts due to optical sectioning, since cell-free WPBs lying flat showed the same vWF pattern (Figures S1G and S1H). vWF is thus organized within WPBs into nanoscopic clusters whose median size correlates to the length unit inferred from high-throughput confocal morphometry (HTM; Figure 2D). Since vWF nanoclusters likely are the physical basis of the “length unit” uncovered by HTM and thus the origin of the WPBs’ discrete sizes (Figure 2B), we called them “quanta.”

vWF is the most abundant WPB cargo (Ewenstein et al., 1987); therefore, at the ultrastructural level, an uneven distribution of vWF within WPBs might be reflected by variations in the organelle content. Electron micrographs of WPBs, imaged within cells or isolated, were consistent with this prediction and displayed regions of higher electron density similar in size to that observed for the vWF quantum (Figures 2E–2G).

### WPB Length Is Determined at the Golgi

**Figure 3:**
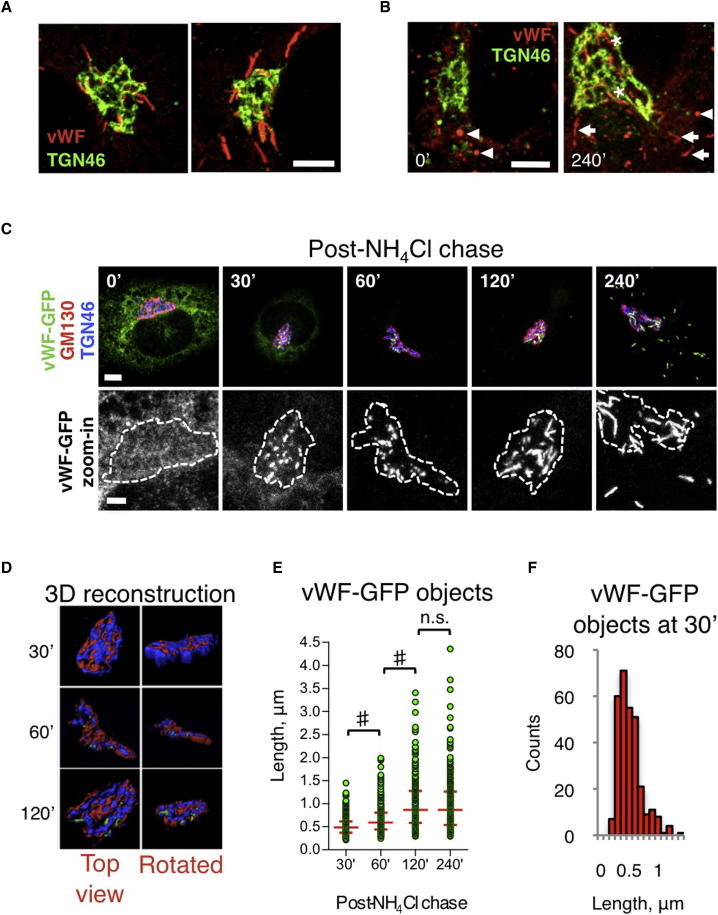
WPB Size Is Acquired before Budding from Golgi (A) WPBs in the Golgi region of untreated HUVECs. Scale bar, 5 μm. (B) WPB formation can be manipulated by NH_4_Cl incubation and washout. NH_4_Cl-treated HUVECs (2 days) were subjected to washout and fixed immediately or after 240 min, and endogenous vWF was visualized. Neutralization of the lumen induces organelle rounding (arrowheads). After Golgi reacidification (240 min), organelles forming at the Golgi were visible (asterisks) and newly made WPBs (elongated shape, arrows) repopulated the cell periphery. Scale bar, 5 μm. (C) The dynamics of WPB formation was analyzed using a vWF-GFP reporter. Dashed lines (zoom-in) outline the Golgi complex (GM130 and TGN46 staining). Scale bar, 5 μm; zoom, 2 μm. (D) 3D reconstruction of the Golgi region of the cells shown in (C); forming WPBs are within the Golgi volume up to 120 min. (E) Length of the vWF-GFP-labeled objects; medians and interquartile ranges are shown. n = 299, 243, 241, and 308 for 30’, 60’, 120’, and 240’, respectively. #p < 10^−10^. (F) Length frequency of vWF-GFP objects at 30 min. See also Movie S1.

The observation of long WPBs at or in close proximity to the Golgi (Figure 3A) is consistent with the notion that their formation is complete before they bud from the TGN (Lui-Roberts et al., 2005; Zenner et al., 2007). However, our findings raised the possibility that WPBs containing two or more quanta could be produced by post-Golgi homotypic fusion of one-quantum intermediates, in a process similar to the biogenesis of other secretory granules (Morvan and Tooze, 2008). vWF is structurally necessary for WPB production, and disrupting its processing and conformational rearrangements by neutralizing the acidic pH of the Golgi lumen prevents formation of WPBs (Wagner et al., 1986; Figure 3B). By coupling vWF-GFP expression to label newly formed WPBs with neutralization/reacidification of the Golgi lumen to control biogenesis, we analyzed the dynamics of WPB formation. At the Golgi, the initially small vWF-GFP-positive objects increased in size for up to 2 hr (Figures 3C–3E), when they resembled nascent Golgi-localized WPBs (refer to Figure 3A). However, between 2 and 4 hr, the time during which the organelles budded from the Golgi and populated the cell periphery, their size remained unchanged (Figures 3C and 3E). Of note, at 30 min, vWF-GFP object length peaked at ∼500 nm (Figure 3F), similar to the vWF unit size identified by HTM and superresolution imaging and suggested by EM. These data indicate that the final length of WPBs is reached during formation at the Golgi and not by postbudding head-on homotypic fusion of short WPBs. This conclusion is also supported by live imaging (Movie S1).

### Dual Role of the Golgi in Determining WPB Size

**Figure 4:**
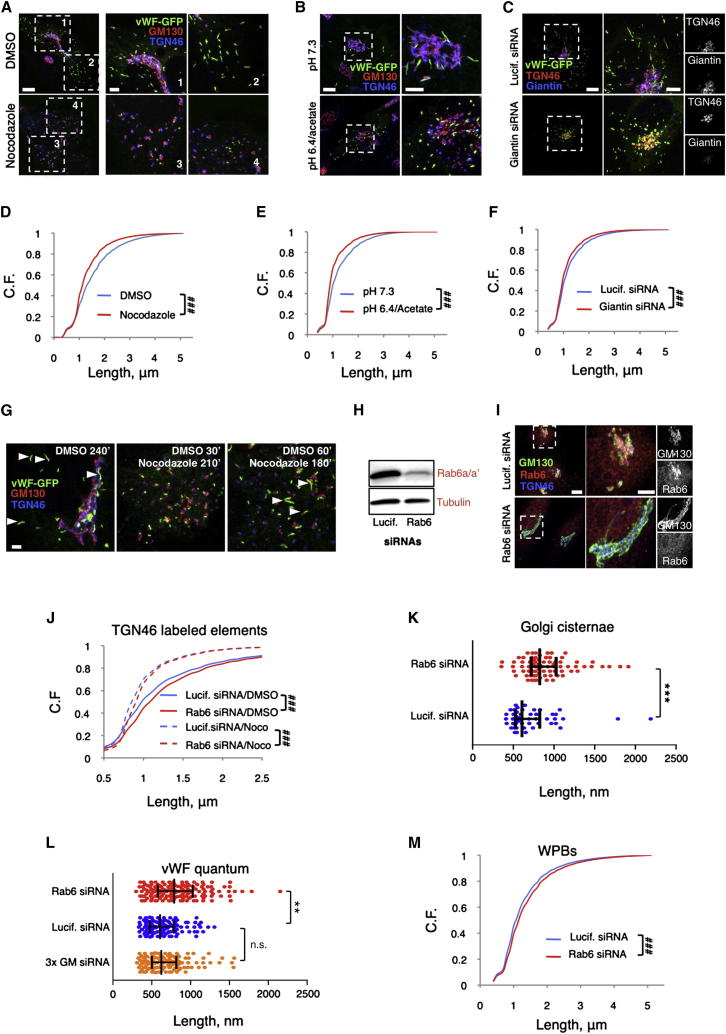
Golgi Ministacks and Ribbon Architecture Determine the Size of WPBs (A–C) Effect of Golgi ribbon unlinking on newly made WPBs (vWF-GFP labeled). Unlinking was induced with nocodazole (A), by lowering the pH of the cytosol in the presence of acetate (B), or by Giantin siRNA (C). Scale bars, 10 μm; magnifications, 5 μm. (D–F) HTM analysis of WPB length in cells treated as in (A)–(C). Endogenous vWF was stained to label the whole organelle population. Cumulative frequencies (C.F.) of the organelle number were plotted as function of organelle length. Number of WPBs analyzed: ∼10^4^–10^6^. ###p < 10^−15^. (G) Cells nucleofected with vWF-GFP were incubated with NH_4_Cl as described in Figure 3. Golgi ribbon was unlinked with nocodazole at the indicated times after NH_4_Cl washout. Arrowheads indicate long WPBs; scale bar, 2 μm. (H and I) siRNA-treatment targeting Rab6a/a’ isoforms resulted in an ∼85% reduction of their protein levels (H), in agreement with the fraction of cells showing loss of Rab6 staining at the Golgi (I). Scale bars, 10 μm; magnified 5 μm. (J) Golgi-positive structures (TGN46 positive) in siRNA-treated cells incubated overnight with DMSO or nocodazole and analyzed by HTM. For each treatment, n = ∼10^5^; ###p < 10^−15^. (K) siRNA-treated cells were incubated with nocodazole to unlink ministacks in order to facilitate quantification. Electron micrographs of cells were inspected and cisternal lengths were measured. Medians (quartiles): luciferase, 612 (529, 827) nm; Rab6, 829 (725, 1,029) nm. n = 47 and 77 for luciferase and Rab6 siRNAs, respectively. ^∗∗∗^p < 10^−3^. (L) vWF quantum size was measured in STORM images of siRNA-treated cells (3× GM indicates GM130, GRASP55, and Giantin triple knockdown). Medians and quartiles are shown. n = 153, 117, and 129 for luciferase, 3× GM, and Rab6 siRNAs, respectively. ^∗∗^p < 10^−2^. (M) HTM analysis of WPB length in siRNA-treated cells. For both treatments, n = ∼8 × 10^5^; *###*p < 10^−15^. See also Figures S2 and S3.

What structural feature of the WPB biogenetic compartment, the Golgi apparatus, might be responsible for the generation of vWF quanta? The Golgi apparatus, as part of the endomembrane system, evolved before the divergence of nucleated cell lineages (Dacks and Field, 2007; Klute et al., 2011; Mowbrey and Dacks, 2009) and almost all eukaryotes share an identical organization of this organelle in the form of stacks of flattened membrane cisternae, which receive cargos on the entry side from the ER and, after processing, release them at the exit side for delivery to their final cellular destination. While in most eukaryotic phyla, the Golgi apparatus is a collection of monocisternal piles (ministacks) separated from one another and scattered throughout the cytoplasm (Donohoe et al., 2013; Kang and Staehelin, 2008; Mogelsvang et al., 2003; Witte et al., 2011), in vertebrates, through mechanisms involving microtubules, motors, tethers, and homotypic cisternal fusion events, the ministacks are linked together into a centralized Golgi apparatus. At the light-microscopy level, this appears as a lace-like perinuclear structure, known as the ribbon (Nakamura et al., 2012). Three-dimensional, ultrastructural imaging shows that within the ribbon, individual ministacks can still be recognized as piled cisternal “compact zones” separated from each other by either interruptions or tubular networks, known as “fenestrated zones” (Ladinsky et al., 1999; Weidman et al., 1993). The basic structure of the ministack is not the only common Golgi feature across eukaryotes; strikingly, its dimensions are conserved as well. Individual cisternae, and thus the ministacks, have sizes in the range of 500–1,000 nm (Donohoe et al., 2013; Kang and Staehelin, 2008; Ladinsky et al., 1999; Mogelsvang et al., 2003; Weidman et al., 1993; Witte et al., 2011). These features led us to hypothesize that the cisternal dimensions within ministacks might limit the size of the vWF quanta. At the exit side of the Golgi, the TGN, especially in HUVECs (Figure S5B), is mostly a continuous compartment within which adjacent quanta could be positioned for copackaging into nascent WPBs, explaining the generation of a population of organelles of variable size (see Figures 7A, S5A, and S5B). This mode of biogenesis predicts that unlinking the Golgi ribbon into separated ministacks should prevent copackaging of vWF quanta at the TGN, resulting in the production of short WPBs (see Figure 7B, “ribbon unlinking”). To test this prediction, we disassembled the ribbon by depolymerizing microtubules with nocodazole, a treatment that reverts the Golgi to the eukaryotic default organization with ministacks juxtaposed to ER exit sites (Cole et al., 1996; Donohoe et al., 2013; Kang and Staehelin, 2008; Mogelsvang et al., 2003; Witte et al., 2011). In nocodazole, only short (≤2 μm) new WPBs were formed (Figures 4A and S2A). The size of these “mini” WPBs seemed to be limited by that of the Golgi elements that generated them and similar to that of the quantum, suggesting that Golgi ministacks make “mini-WPBs,” mostly containing a single vWF quantum, a conclusion supported by nanoscopy (Figures S2B–S2D). Ultrastructurally, these short organelles appeared morphologically normal (Figure S2I). Two alternative experimental approaches confirmed the effects of unlinking the Golgi ribbon on the size of newly made WPBs. In one, we lowered the cytosolic pH in the presence of acetate, which results in separated ministacks while leaving the microtubules unaffected (Figures 4B and S2E; Yoshida et al., 1999). In the other, we depleted three Golgi matrix proteins (GM130, GRASP55 [not shown], and Giantin) individually or together (Figures 4C, S2F, and S2G). The roles of these proteins in ribbon maintenance and ectopic ribbon formation were previously reported (Feinstein and Linstedt, 2008; Heuer et al., 2009; Koreishi et al., 2013; Puthenveedu et al., 2006). Irrespective of the ribbon-unlinking method used, the effect on the size of WPBs made during the experiments was sufficient to shift the length of the entire organelle population (Figures 4D–4F and S2H) while leaving the quantum size unchanged (Figure 4L). Finally, unlinking the ministacks before (30 min, Figures 3C and 3E) or soon after (60 min, Figures 3C and 3E) vWF-GFP objects began to grow in size during organelle formation showed that once WPB elongation (i.e., quanta copackaging) occurred, the ribbon was no longer required for the production of long organelles (Figure 4G).

Our model also predicts that if the cisternal dimensions limit those of the quantum, then changing the size of the cisternae should alter that of the quanta and, ultimately, that of WPBs (see Figure 7B, “cisternal lengthening”). Knockdown of Rab6a/a′ isoforms results in longer Golgi cisternae (Storrie et al., 2012), an effect that should be measurable as larger ribbons and, when they are unlinked, as larger ministacks. We confirmed both of these outcomes of Rab6a/a′ knockdown by HTM and by measuring cisternal length in electron micrographs (Figures 4H–4K, S3A, and S3B). vWF quantum size was increased in Rab6-siRNA treated cells (Figure 4L), an event reflected in a shift to higher values of WPB length clusters (Figure S3C). Finally, WPB size was increased in Rab6-depleted HUVECs, as was that of mini-WPBs upon ribbon unlinking (Figures 4M and S3D).

The effects of Golgi unlinking and cisternal lengthening on the sizes of the vWF quantum and WPBs support our biogenesis model (see Figure 7) and stress a requirement for the ribbon architecture of the Golgi to generate long WPBs from the quanta molded within the ministacks (Figure S5A).

### WPB Number and Size Depend on Cargo Availability

**Figure 5:**
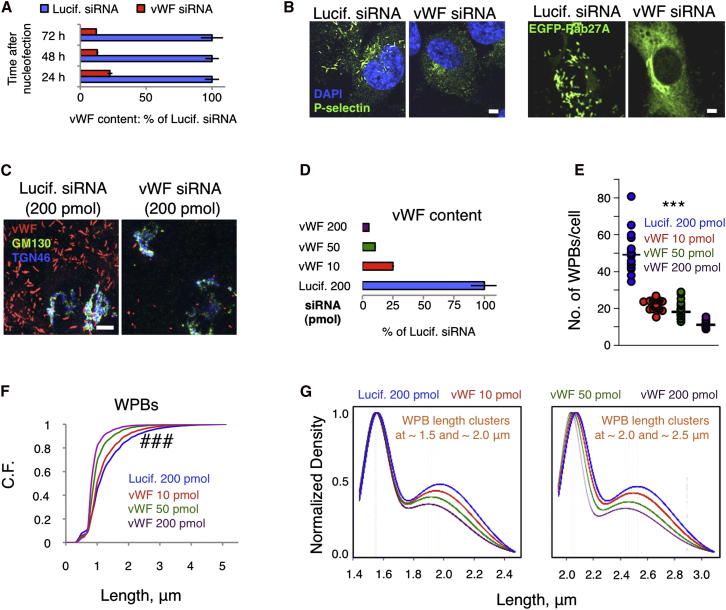
vWF Levels Control WPB Number and Size (A) Time course of siRNA-mediated vWF depletion (at 200 pmol siRNA/nucleofection reaction). vWF content was normalized to protein in cell lysates; mean ± SD (n = 3). (B and C) vWF depletion causes mislocalization of the WPB markers P-selectin and Rab27A (B) and a reduction in size of the residual organelles (C). Scale bars, 5 μm. (D) vWF cell content was modulated by titrating the amount of vWF-targeting siRNA delivered; mean ± SD. (E) HTM analysis of the number of WPBs per cell following vWF-targeting siRNA titration; median values are shown (n = 24 per treatment, with each observation from a separate well of 96-well plates); ^∗∗∗^p < 10^−3^ for all the pairwise comparisons. (F) Decrease in vWF cell content results in shortening of WPBs. Per treatment, n = 2.3 × 10^5^ to 8 × 10^5^; ###p < 10^−15^ for all pairwise comparisons. (G) Kernel densities for each data set in the indicated length ranges were calculated as in Figure 2B and normalized to the length cluster of highest density. The distance between the length clusters was unaffected (Table S2), indicating that vWF quantum size remains constant over an ∼20-fold change in cargo abundance (see D, Lucif. 200 pmol versus vWF 200 pmol).

One model of organelle size control invokes regulation by component availability, that is, abundance of structural and enzymatic components will determine the size or number of organelles (Goehring and Hyman, 2012). In the case of WPBs, it is well established that the cargo protein vWF is the driver for organelle formation (Wagner et al., 1991). We observed that the siRNA-mediated reduction of vWF protein levels resulted in a decrement not only of the number of WPBs, as might be expected, but also of the length of the residual organelles (Figure 5C). We tested the impact of vWF levels on the length of WPBs by titrating the vWF-targeting siRNA. The consequent gradual reduction in vWF cellular content correlated with a gradual decrease in the number of WPBs and, importantly, a shortening of WPB length (Figures 5D–5F). In contrast to the number and length of WPBs, the quantum size, as measured by the distance between length clusters, remained constant upon progressive reduction of vWF cell content (Figure 5G; Table S2). Control of the quantum size, therefore, seems to be independent of vWF levels and primarily set by cisternal dimensions (see Figure 7C).

### Functional Effects of WPB Size

**Figure 6:**
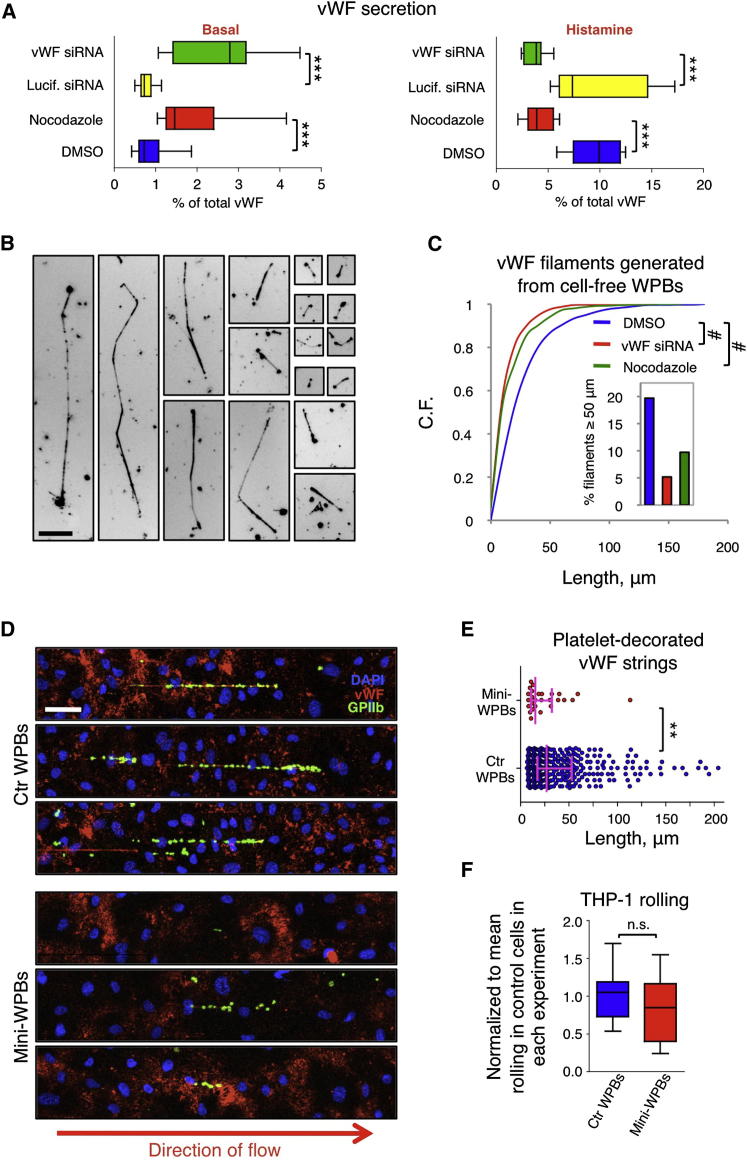
WPB Size and Endothelial Function (A) vWF release in the absence or presence of histamine measured from control (200 pmol luciferase siRNA/reaction or DMSO) and mini-WPB enriched HUVEC cells (200 pmol vWF siRNA/reaction or nocodazole). Box plots of 9–18 measurements per treatment from 3–4 experiments are shown. ^∗∗∗^p < 10^−3.^ (B) Cell-free WPBs were diluted and permeabilized to obtain well-separated vWF filaments. Representative images from control, VWF siRNA-, and nocodazole-treated samples are shown. Scale bar, 25 μm. (C) Cumulative frequency of vWF filament number as a function of filament length generated by cell-free WPBs obtained from HUVECs treated as indicated. DMSO, n = 858; vWF siRNA, n = 851; nocodazole, n = 885. #p < 10^−10^. Inset: percentage of long filaments in each treatment. (D) Formation of platelet-decorated vWF strings under flow following HUVEC stimulation with histamine. Platelets were identified by CD41/GPIIb labeling. (E) Length of platelet-decorated vWF strings; median and interquartile ranges are shown. Control, n = 366; mini-WPBs, n = 27 from 2 separate experiments. ^∗∗^p < 10^−2^. (F) Rolling of THP-1 monocyte-like cells on HUVEC monolayers following histamine stimulation; box plot of 12 measurements from 4 separate experiments. See also Figure S4.

Long WPBs are a minority but contain a considerable fraction of vWF cargo (Figure S1D), suggesting that this subpopulation of organelles might be especially important. We assayed the secretory behavior of cells enriched in short WPBs. To prompt the formation of mini-WPBs, we performed the two manipulations that had been shown to control organelle size: low levels of vWF expression and Golgi ribbon unlinking. We used nocodazole to unlink the ribbon, but before assaying secretion, we subjected the cells to washout and a short chase to allow reformation of microtubules (Figure S4A). Although working through different mechanisms, both of these size-reducing treatments had the same effect: vWF secretion was enhanced in the absence of secretagogue and reduced in its presence when compared with control cells (Figure 6A). Since ∼80% of synthesized vWF is stored in WPBs and its basal secretion occurs from WPBs (Giblin et al., 2008), the secretory phenotype observed suggested that short WPBs are less responsive to secretory agonists and are the main contributors to basal secretion of vWF. WPB size might therefore represent an important factor in the prothrombotic function of endothelial cells.

Although it may seem obvious that the size of WPBs is reflected in their hemostatic capability, structural considerations highlight that this relationship in fact cannot be predicted. vWF strings provide the adhesion platforms for circulating platelets in primary hemostasis (De Ceunynck et al., 2013; Dong et al., 2002). Strings are formed from secreted multimerised vWF, and multimer size affects string length (Nightingale et al., 2009). During biosynthesis, an unknown fraction of the total vWF is tubulated, a conformational arrangement that seems to be required for multimer formation (Huang et al., 2008; Zhou et al., 2011; summarized in Figure 1).

The largest multimers found in WPBs (Figure 1E) with ∼50 subunits (i.e., ∼25 dimers) in the coiled configuration would span only ∼63 nm of a tubule length (4.2 dimers per turn with a 11 nm pitch; see Figure 1; Huang et al., 2008). Therefore, the structural data suggest that tubules are for the most part composed of more than one multimer. Similarly, at exocytosis, a vWF 50-mer would extend for ∼3 μm (∼60 nm per subunit; see Figure 1D; Fowler et al., 1985), a size that alone cannot account for that of the vWF platelet-catching strings, some of which extend up to hundreds of micrometers (De Ceunynck et al., 2013). To establish whether organelle size does indeed have an effect on WPB hemostatic function, we experimentally addressed the issue.

Cell-free mini-WPBs produced shorter vWF filaments than control WPBs (Figures 6B, 6C, and S4D), confirming that experimentally induced mini-WPBs are structurally normal, since they store vWF that can make multimers and filaments (Figures 6B and S4C), and strongly suggesting a link between organelle length and hemostatic capability.

To test this more directly, we assayed vWF string formation and platelet capture by HUVECs under flow. In these experiments, mini-WPBs in HUVECs were obtained by nocodazole treatment, which had only a minimal effect on the number of WPBs per cell compared with vWF knockdown (Figures 5E and S4B). No platelet-decorated vWF strings were formed in unstimulated control or mini-WPB-bearing cells (Figure S4E). However, in the presence of histamine, platelet-decorated vWF string generation was strongly reduced from cells with short WPBs, and those fewer strings were also shorter (Figures 6D, 6E, and S4F). Of note, the extent of string number reduction could not be accounted for by the modest reduction in WPB numbers in nocodazole-treated cells (Figures S4B and S4F), suggesting that string formation might be cooperative in nature and thus subject to threshold effects. These data show that the subpopulation of long WPBs is disproportionately important for the production of vWF strings efficient in platelet recruitment.

In response to inflammation, circulating leukocytes are recruited to the affected tissues. Signaling to endothelial cells prompts exocytosis of WPBs, whose membrane cargo, P-selectin, becomes exposed in the vessel lumen, where it functions by binding circulating leukocytes. By decelerating the leukocytes, this interaction allows them to roll along the vessel wall and then firmly adhere to it, as required for their extravasation into the inflamed tissue (Mayadas et al., 1993). We tested whether the size of WPBs had any impact on this function, by measuring the rolling of THP-1 monocyte-like cells on HUVECs under conditions in which leukocyte-endothelial cell interactions depend exclusively on P-selectin (Doyle et al., 2011). Histamine stimulation induced this interaction in control cells and their mini-WPB-enriched counterparts to the same extent (Figures 5F and S4G). Thus, the proinflammatory function of WPBs is not affected by their size.

## Discussion

**Figure 7:**
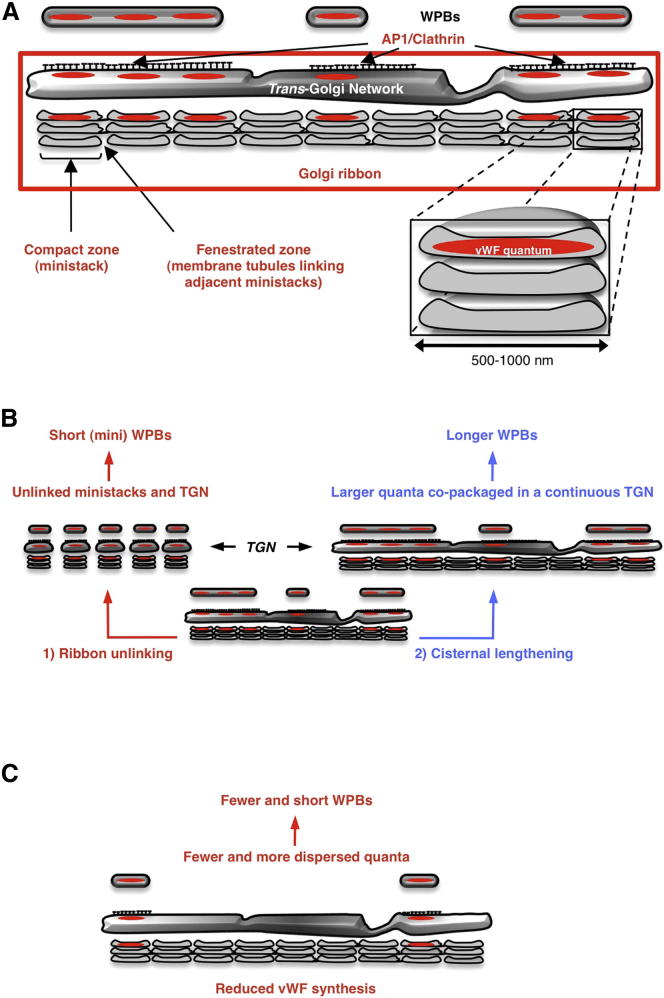
Structural Units and Compound Architecture of the Golgi Cooperate to Generate WPB Size (A) In vertebrates, the Golgi’s structural units, the ministacks, are brought into close proximity by motors and microtubules and fuse to their neighbors via tubular connections between homologous cisternae (fenestrated zones), generating a higher-order architecture, the ribbon (boxed in red). The cisternal dimensions limit the size of the vWF quanta, but in the continuous lumen of the TGN, adjacent quanta can be copackaged together into forming organelles. This process requires the adaptor complex AP1 and clathrin, which likely act as a scaffold (Lui-Roberts et al., 2005). The size of WPBs generated in this process depends on the number of copackaged quanta. (B) Unlinking the ribbon into separated ministacks has no effect on quantum size, but partitions the TGN and prevents multiple quanta copackaging. This results in the formation of short WPBs (pathway 1). Increasing the dimension of Golgi cisternae allows formation of bigger vWF quanta, whose copackaging produces longer WPBs (pathway 2). (C) Reduced synthesis of vWF results in fewer and more dispersed quanta, which are packaged in fewer and shorter WPBs. See also Figure S5.

We report a previously unappreciated role for the Golgi apparatus in controlling the size of a secretory carrier. This control requires cooperation between the two structural levels of this fundamental organelle: its functional unit, the ministack, and its compound architecture, the ribbon. Our data support a “two-tier” model of control of organelle size during biogenesis: within the ministacks, cisternal dimensions limit those of the forming vWF quanta, which in the continuous lumen provided by the TGN of linked ministacks can then be copackaged into nascent WPBs of sizes determined by the number of quanta they contain (Figures 7 and S5).

The Golgi apparatus is almost universally present in eukaryotes as scattered ministacks (Donohoe et al., 2013; Kang and Staehelin, 2008; Kondylis et al., 2007; Mogelsvang et al., 2003; Witte et al., 2011) and the mammalian Golgi ribbon can be reverted to this “default” organization without major defects in secretion (Cole et al., 1996). Why vertebrate cells need a ribbon is not fully understood, though experimental evidence suggests that this peculiar architecture might be required for the homogeneous distribution of processing enzymes, polarized secretion, cell migration, and function as a mitotic checkpoint (Miller et al., 2009; Puthenveedu et al., 2006; Rabouille and Kondylis, 2007; Yadav et al., 2009). Now we find that endothelial cells exploit the presence of a ribbon to produce a subset of long WPBs that are required for recruitment of platelets, a newly appreciated function for this Golgi superstructure.

Control of size endows WPBs with plasticity by allowing the uncoupling of their hemostatic and proinflammatory activities. Long WPBs are necessary for efficient platelet recruitment, a key event in primary hemostasis, but are dispensable for leukocyte rolling on endothelia, a process mediated by P-selectin and necessary for an adequate inflammatory response (Mayadas et al., 1993). P-selectin, the principal leukocyte receptor in the initial inflammatory response, is sorted to the WPB membrane during formation of these organelles by interacting with vWF (Bonfanti et al., 1989; Michaux et al., 2006b). Storage of P-selectin in WPBs is required for its function, since endothelial cells of vWF-deficient mice, which are devoid of WPBs but maintain levels of P-selectin similar to those in wild-type cells, display impaired leukocyte rolling and extravasation (Denis et al., 2001). Our finding that the production of short WPBs does not affect leukocyte recruitment in vitro indicates that vWF-dependent P-selectin sorting to the smaller organelles and thus the function of those organelles in inflammation are unaffected.

The discovery of WPB plasticity suggests a potential capability for vascular beds to regulate, spatially and/or temporally, their prothrombotic propensity by controlling the size of the WPBs they produce. Locally and over time, an endothelial district could modulate its own function by two mechanisms: transcriptional regulation of vWF cellular levels and changes in the Golgi architecture. Signaling networks modulate the architecture of the Golgi apparatus (Chia et al., 2012); therefore, pathways driving a lower or higher degree of ministack linking into a ribbon are bound to result in the production of short or long WPB cohorts, respectively, with consequences for their hemostatic activity. It has been reported that endothelial expression of the transcription factor KFL2 induces formation of short WPBs (van Agtmaal et al., 2012). Our findings suggest the intriguing possibility that the antithrombotic status induced by KLF2 (Lin et al., 2005) might be mediated at least in part by its effects on WPB size. Since expression of KLF2 does not reduce but actually upregulates vWF levels (Dekker et al., 2006; van Agtmaal et al., 2012), its effects on WPB size might be mediated through regulation of the Golgi ribbon, a hypothesis worth future study.

Size control of cellular organelles seems to be required in many developmental and homeostatic processes (Kirk et al., 2010; Levy and Heald, 2010, 2012; Rafelski et al., 2012; Sardiello et al., 2009; Schuck et al., 2009; Settembre et al., 2012). One mode of size control invokes component-mediated regulation, whereby levels of structural components, such as proteins and lipids, determine the organelle volume/size (Chan and Marshall, 2010; Goehring and Hyman, 2012). For single-copy organelles, component upregulation can thus lead to expansion, as in the case of the ER (Kirk et al., 2010; Schuck et al., 2009), whereas in the case of multicopy organelles, it can modulate the combined compartment volume by adjusting the number, as lysosomes do (Sardiello et al., 2009).

WPBs conform to the component-mediated size-control model by combining the modalities for single- and multicopy organelles: the expression levels of vWF, the cargo that is structurally necessary for organelle formation, impact not only the number but also the length of these secretory granules. This peculiarity might be explained by the existence of the vWF quantum. The Golgi will organize lower levels of vWF into fewer, more dispersed quanta that are not copackaged, thus generating both fewer and smaller WPBs (Figure 7C).

With regard to the supramolecular nature of the quantum, further investigations are needed to clarify its relationship to the other vWF structure observed within WPBs, the tubule. It is generally assumed (but remains to be proved) that vWF sorted into WPBs is present only in its tubulated form. Electron micrographs of WPB transverse sections often show amorphous content covering a large area interspersed with tubules, a morphological arrangement that has sparked speculation about the presence of an intertubule scaffold (Valentijn et al., 2008). Since vWF is the most abundant protein in WPBs (Ewenstein et al., 1987), it is reasonable to conclude that much of this cargo is not tubulated and might therefore provide the suggested “scaffold.” STORM images reflect antibody labeling and may detect vWF pools that overlap with the fraction structured into tubules only partially or not at all. Irrespective of its supramolecular nature, the quantum reflects either different vWF concentrations (as suggested in part by the inhomogeneous electron density in our EM data) and/or antibody accessibility. We also note that vWF tubules are almost invariably straight, an indication that they are stiff structures. Were all tubules to extend for the entire length of an organelle, it would be difficult to reconcile this rigidity with live imaging data showing that WPBs do bend, a clear sign of structural flexibility (Zenner et al., 2007). A possibility is therefore that the quantal arrangement of vWF may confer or reflect structural flexibility in long organelles.

The distinctive cigar-like shape of WPBs is of physiological significance because it mirrors the lumenal arrangement of vWF cargo in tubules. Perturbing the structure of the tubules results in round WPBs that respond normally to secretagogues but release tangled vWF strings that bind fewer platelets (Michaux et al., 2006a). Here, we report how size is also critical to WPB function.

## Experimental Procedures

### Cells, Culture Conditions, and Nucleofection

HUVECs from multiple donors were obtained from TCS Cellworks or Lonza. Cells were maintained as previously described (Zenner et al., 2007) and used within passage 4. Plasmids and siRNAs were introduced by nucleofection (Lonza) using a constant number of cells (2 × 10^6^) per reaction. See Supplemental Experimental Procedures for further details.

### Confocal Microscopy

Cells were fixed with 4% formaldehyde in PBS. Unless otherwise specified, samples were permeabilized and then incubated with primary antibodies followed by secondary antibodies conjugated to Alexa Fluor dyes (Molecular Probes, Life Technologies) or Cy5 (Jackson ImmunoResearch Laboratories). Mounted samples (ProLong Gold antifade reagent, Life Technologies) were imaged with a 63× oil immersion objective (NA 1.3) on a Leica Microsystems TCS SPE confocal system. Maximum intensity projections of image stacks (0.5 μm z step) are shown unless stated otherwise. See Supplemental Experimental Procedures for details.

### High-Throughput Confocal Microscopy

Cells cultured in 96-well plates were fixed and immunostained to label WPBs. Hoechst 33342 (Life Technologies) was used to counterstain nuclei. An Opera High Content Screening System (Perkin Elmer) was used for image acquisition of 5 to 25 fields of view per well (depending on the experiment) using a 40× air objective (NA 0.6). Image processing and parameter measurements from HTM are detailed in the Supplemental Experimental Procedures.

### Stochastic Optical Reconstruction Microscopy

Cells were immunostained as described for confocal microscopy. Images were acquired using a modified Olympus IX71 inverted objective-based total internal reflection (TIRF) microscope. The detailed workflow is available in Supplemental Experimental Procedures.

### Electron Microscopy

Chemically fixed or high-pressure frozen and free-substituted (HPF/FS) samples were processed as previously described (Michaux et al., 2006a; Zenner et al., 2007). For whole mounts of cell-free WPBs, samples were adsorbed for 10 min on formvar, carbon-coated, and glow-discharged copper grids (Agar Scientific) before fixation in 2% paraformaldehyde/1.5% glutaraldehyde in 0.1 M sodium cacodylate for 1 hr. After osmication and serial dehydration to 100% dry ethanol, the grids were critical-point dried using a Leica EM-CPD300 (Leica Microsystems). All samples were imaged with a Morada camera (OlympusSIS) in a Tecnai20 (FEI). iTEM software (Olympus SIS) was used to measure cisternal length of ministacks in nocodazole-treated luciferase- and Rab6a/a′-siRNA-treated HUVECs.

### Platelet-Decorated vWF Strings and Leukocyte Rolling

Recruitment assays of platelets or THP-1 cells under flow by resting or secretagogue stimulated endothelial monolayers were carried out essentially as previously described (Doyle et al., 2011; Michaux et al., 2006a). See Supplemental Experimental Procedures for details.

### Statistical Analysis

Nonparametric, two-tailed, two-independent-sample Wilcoxon rank-sum test was used. Data sets were analyzed using R (http://www.r-project.org/; see Supplemental Experimental Procedures; p values calculated up 10^−15^) or SOCR (Statistical Online Computational Resource, at University of California, Los Angeles; http://www.socr.ucla.edu/SOCR.html) for calculation of p values up to 10^−10^.

## Supplemental Information

Document S1. Supplemental Experimental Procedures, Figures S1–S5, and Tables S1 and S2

Movie S1. vWF-Positive Objects Merge within the Golgi to Generate a WPB, Related to Figure 3 HUVECs were nucleofected with vWF-GFP plasmid and incubated with BODIPY ceramide to label the Golgi apparatus. Image stacks were acquired every 10 s and 3D reconstructed with IMARIS. Two vWF-GFP-positive objects (highlighted in orange) are seen to merge within the volume of the Golgi apparatus (rendered in transparent gray) and move to the cell periphery as a single object.

Document S2. Article plus Supplemental Information
