# Supplementary material for: A Two-Tier Golgi-Based Control of Organelle Size Underpins the Functional Plasticity of Endothelial Cells
Source: Dev Cell. 2014 May 12;29(3):292–304. doi: 10.1016/j.devcel.2014.03.021 (PMC4022834; doi:10.1016/j.devcel.2014.03.021)
Supplement: Document S1. Supplemental Experimental Procedures, Figures S1–S5, and Tables S1 and S2 [file mmc1.pdf]

Developmental Cell, Volume *29*

Supplemental Information

A Two-Tier Golgi-Based Control of Organelle Size Underpins the Functional Plasticity of Endothelial Cells

Francesco Ferraro, Janos Kriston-Vizi, Daniel J. Metcalf, Belen Martin-Martin, Jamie Freeman, Jemima J. Burden, David Westmoreland, Clare E. Dyer, Alex E. Knight, Robin Ketteler, and Daniel F. Cutler

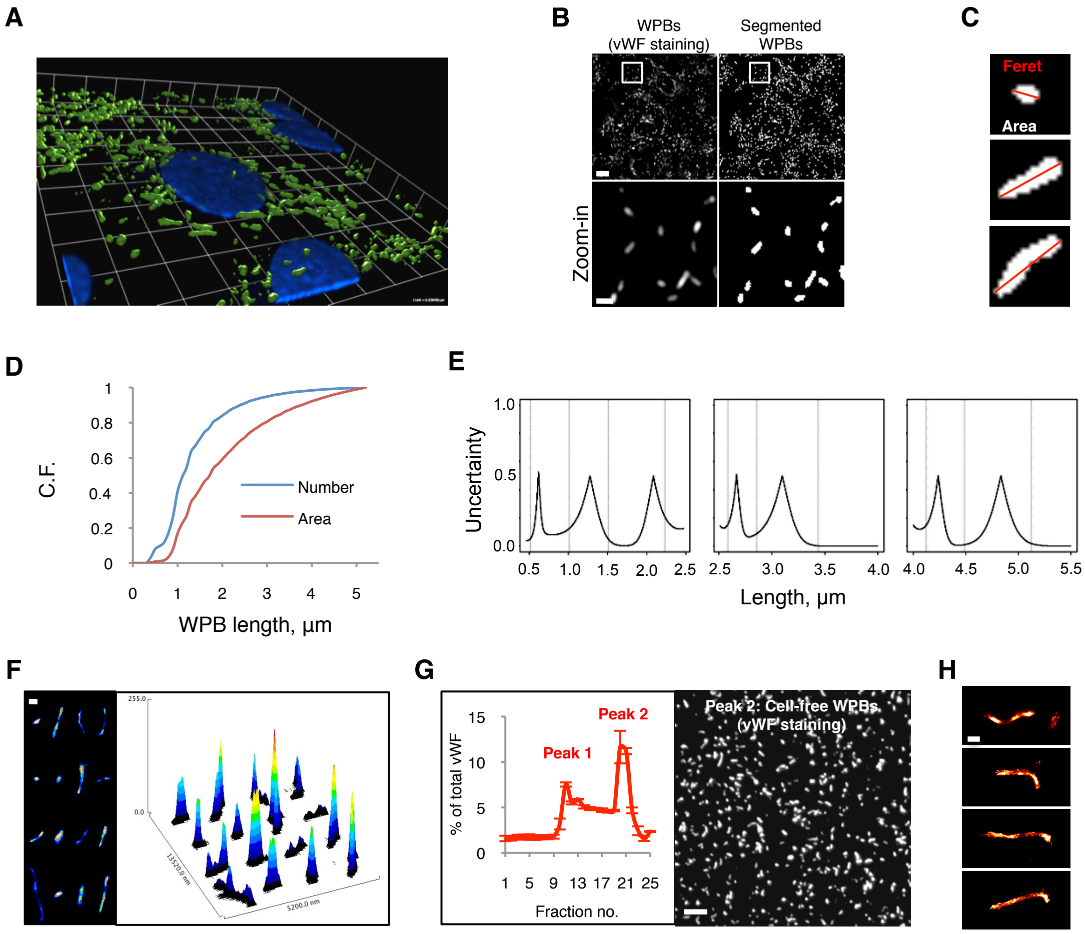
A
B
WPBs
(vWF staining)
Segmented
WPBs
Zoom-in
C
Feret
Area
D
C.F.
Number
Area
WPB length, µm
E
Uncertainty
Length, µm
F
G
% of total vWF
Peak 1
Peak 2
Fraction no.
Peak 2: Cell-free WPBs
(vWF staining)
H

Figure S1

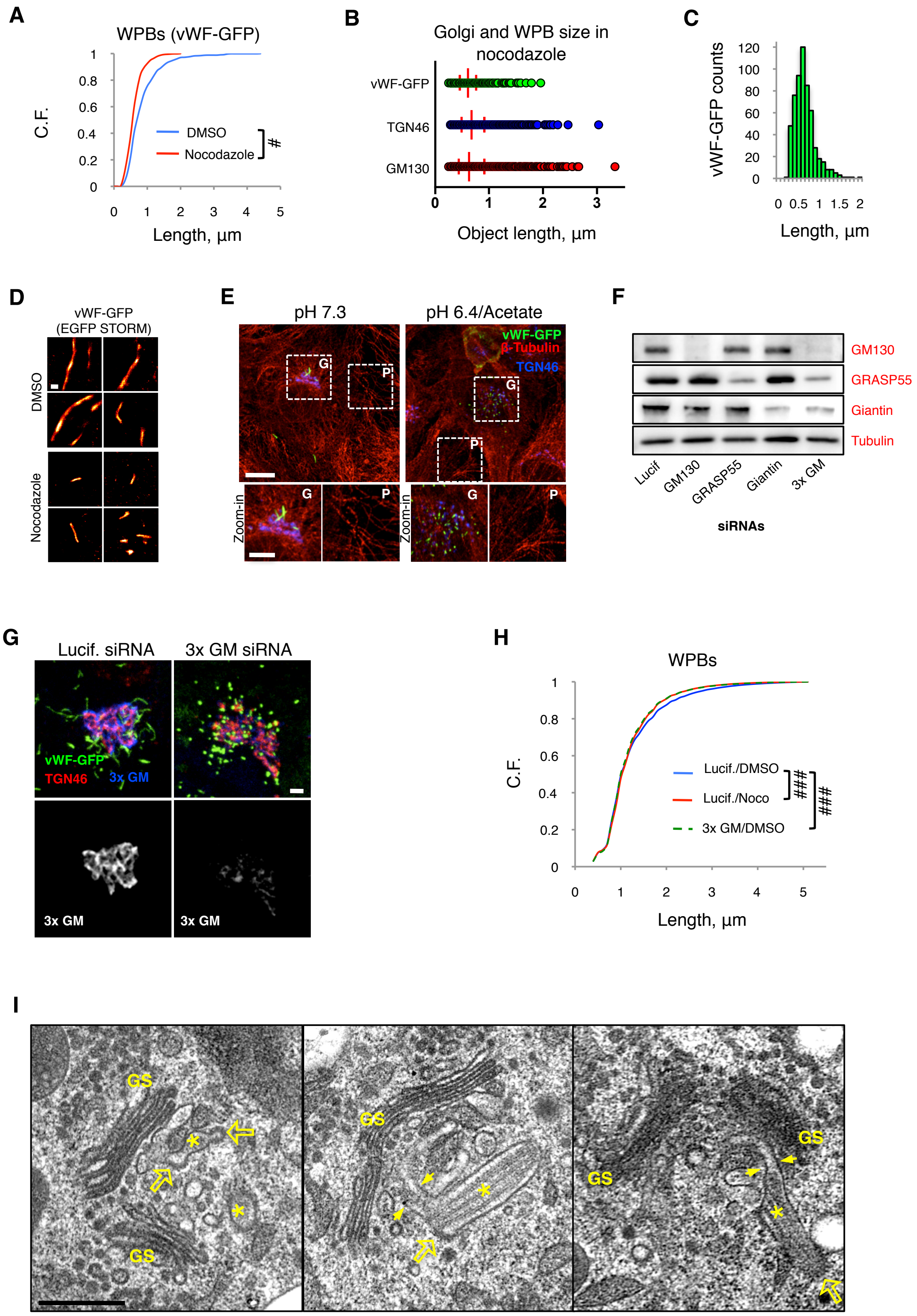
A
WPBs (vWF-GFP)
C.F.
DMSO
Nocodazole
#
Length, µm
B
Golgi and WPB size in nocodazole
vWF-GFP
TGN46
GM130
Object length, µm
C
vWF-GFP counts
Length, µm
D
vWF-GFP (EGFP STORM)
DMSO
Nocodazole
E
pH 7.3
pH 6.4/Acetate
vWF-GFP
β-Tubulin
TGN46
Zoom-in
F
GM130
GRASP55
Giantin
Tubulin
Lucif
GM130
GRASP55
Giantin
3x GM
siRNAs
G
Lucif. siRNA
3x GM siRNA
vWF-GFP
TGN46
3x GM
H
WPBs
C.F.
Lucif./DMSO
Lucif./Noco
3x GM/DMSO
###
###
Length, µm
I
GS

Figure S2

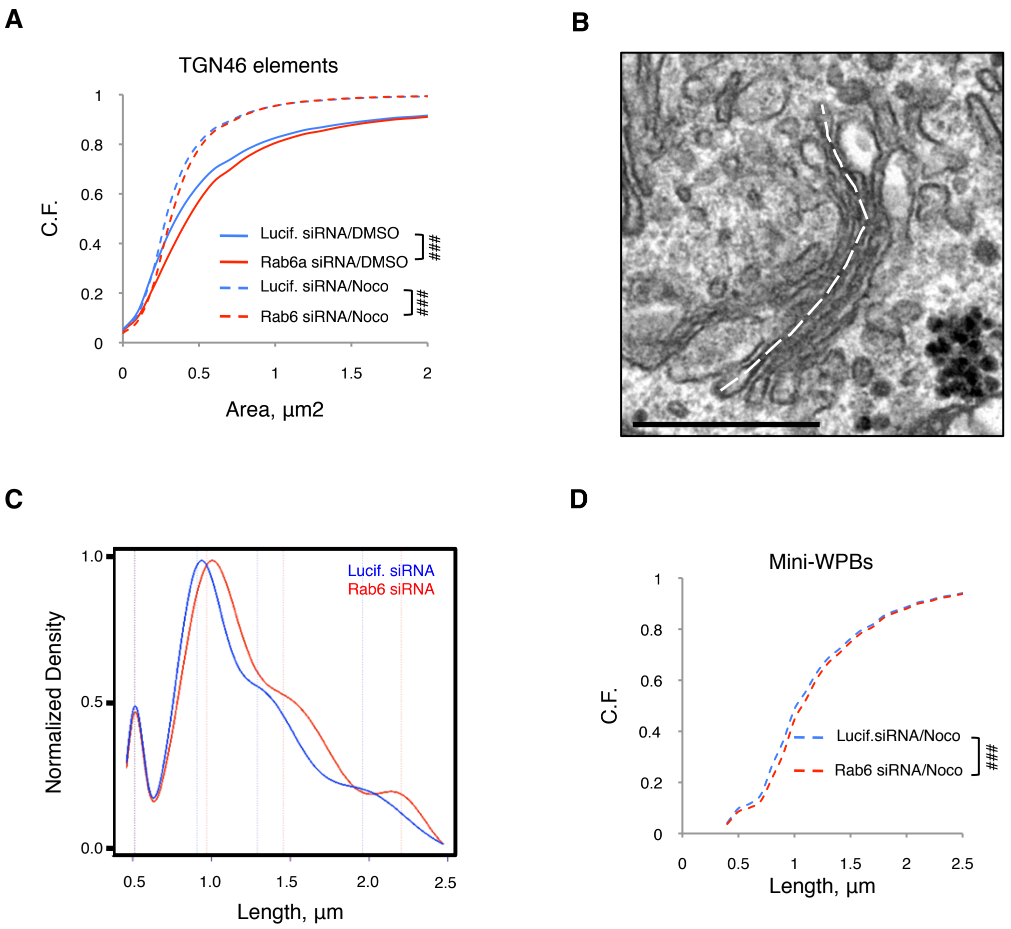
A
TGN46 elements
C.F.
1
0.8
0.6
0.4
0.2
0
0
0.5
1
1.5
2
Area, µm2
Lucif. siRNA/DMSO
Rab6a siRNA/DMSO
###
Lucif. siRNA/Noco
Rab6 siRNA/Noco
###
B
C
Normalized Density
1.0
0.5
0.0
0.5
1.0
1.5
2.0
2.5
Length, µm
Lucif. siRNA
Rab6 siRNA
D
Mini-WPBs
C.F.
1
0.8
0.6
0.4
0.2
0
0
0.5
1
1.5
2
2.5
Length, µm
Lucif.siRNA/Noco
Rab6 siRNA/Noco
###

Figure S3

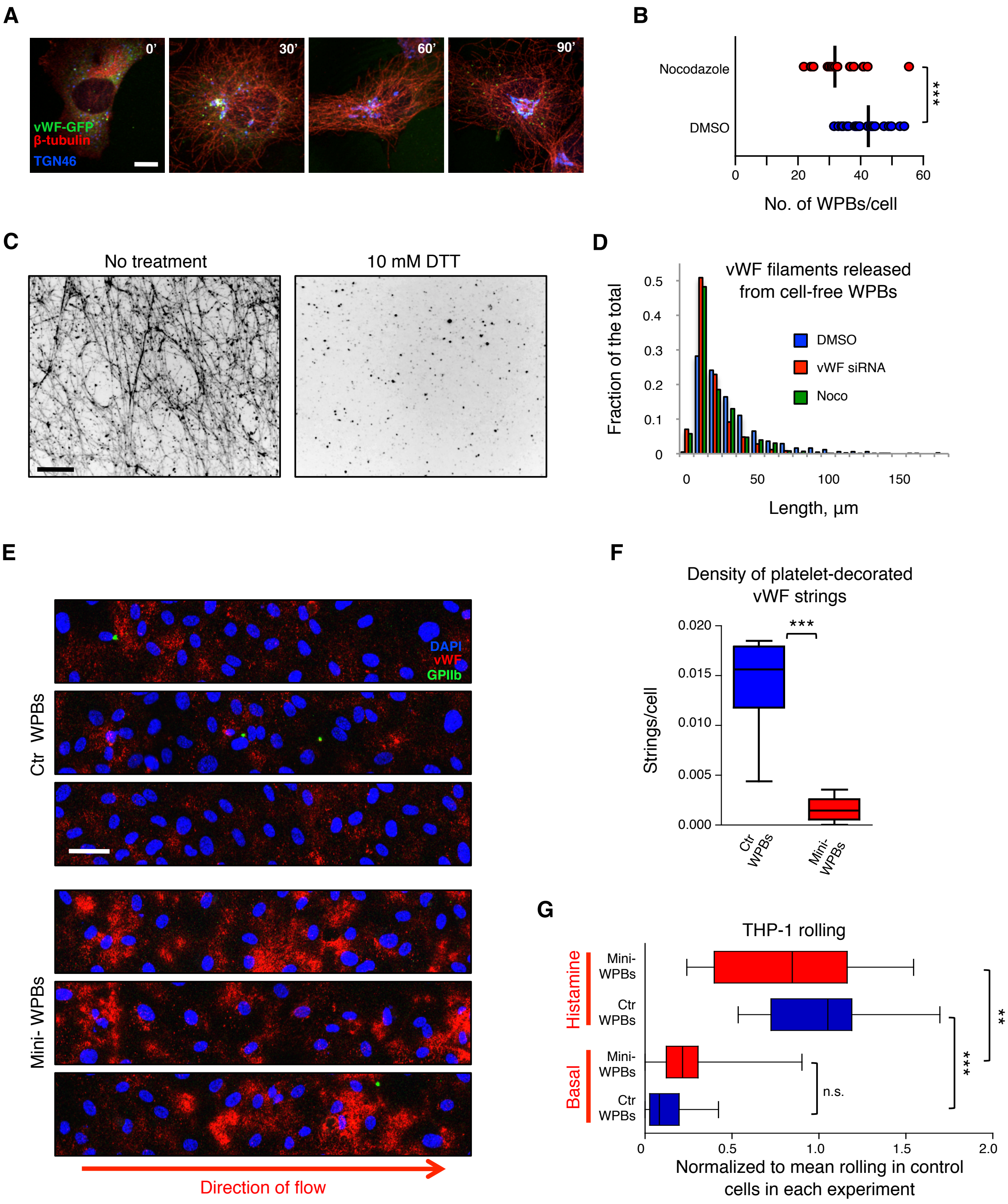
A
0'
30'
60'
90'
vWF-GFP
β-tubulin
TGN46
B
Nocodazole
DMSO
***
0
20
40
60
No. of WPBs/cell
C
No treatment
10 mM DTT
D
vWF filaments released
from cell-free WPBs
DMSO
vWF siRNA
Noco
Fraction of the total
0.5
0.4
0.3
0.2
0.1
0
0
50
100
150
Length, µm
E
DAPI
vWF
GPIIb
Ctr WPBs
Mini- WPBs
Direction of flow
F
Density of platelet-decorated
vWF strings
Strings/cell
0.020
0.015
0.010
0.005
0.000
***
Ctr
WPBs
Mini-
WPBs
G
THP-1 rolling
Histamine
Basal
Mini-
WPBs
Ctr
WPBs
Mini-
WPBs
Ctr
WPBs
**
***
n.s.
0
0.5
1.0
1.5
2.0
Normalized to mean rolling in control
cells in each experiment

Figure S4

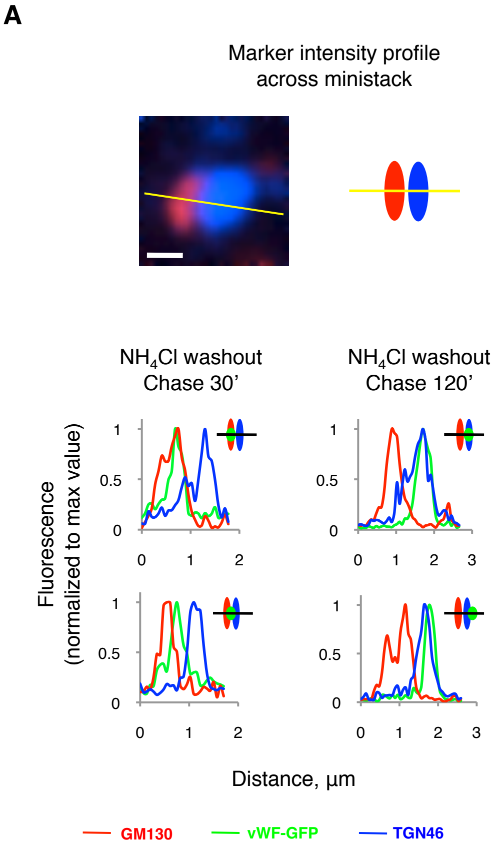
A
Marker intensity profile
across ministack
NH4Cl washout
Chase 30'
NH4Cl washout
Chase 120'
Fluorescence
(normalized to max value)
1
0.5
0
0
1
2
3
Distance, µm
GM130
vWF-GFP
TGN46

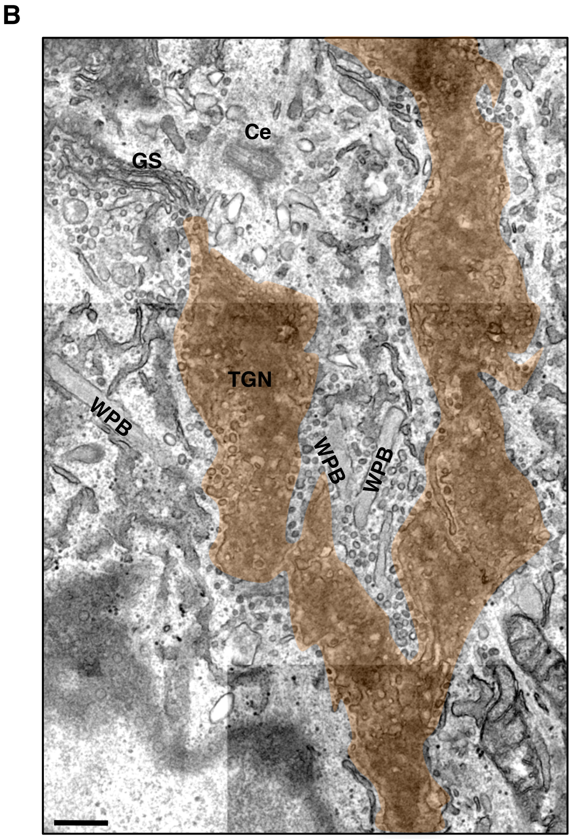
B
Ce
GS
TGN
WPB
WPB
WPB

Figure S5

Figure S1. Related to Figure 2. **High-throughput confocal morphometry and STORM imaging of WPBs**. (A) 3D-rendering of a confocal image stack of endothelial cells in which vWF was stained to label WPBs. Endothelial cell flatness forces WPBs to orient parallel to the substrate. This spatial constraint ensures that WPB lengths measured from 2D confocal images are a good approximation of actual organelle lengths. (B) High-throughput imaging and WPB segmentation. Top panels; left, a representative image acquired by the Opera LX confocal microscope; right, the same image after WPB segmentation (see Supplemental Experimental Procedures). Bottom panels; magnification of the insets shows that segmentation results in accurate identification of WPBs. Scale bar, 10 μm; zoom-in, 2 μm. (C) Two morphological parameters were measured. The "Feret diameter" (red lines) is the maximum distance between two points along the perimeter of one object and approximates the length of a WPB. The area is the sum of the pixels in the segmented object. (D) WPB number and area from the HTM survey data (cumulative frequencies plotted as function of organelle length). Long WPBs represent a minority of the total population (WPBs ≥ 3 μm are ~ 5%), but contain a significant amount of vWF (WPBs ≥ 3 μm account for ~ 20% of the total area covered by the segmented organelles). (E) The uncertainty in the attribution of any object (WPB) to two neighbouring length clusters in the HTM survey dataset. Low uncertainties corresponded to the density maxima identified by the Gaussian mixture modelling shown in Figure 2B (and indicated by the vertical lines), further supporting the presence of length clusters. (F) A montage of WPBs from vWF STORM images. Intensity values represent the number of accepted fluorophore localizations assigned to each pixel (20 nm x 20 nm), with 1 localization corresponding to 1 grey-scale intensity value. Intensities were color-coded for display purposes; on the right, intensity surface plots are shown. (G) Cell-free WPBs were obtained by centrifugation of HUVEC post-nuclear supernatants on discontinuous nycodenz gradients (see Extended Experimental Procedures). Peak 2 membranes, with the highest content of vWF, were adsorbed on Maxisorp™ surface (NUNC) by gentle centrifugation, fixed and processed for vWF immuno-fluorescence. Cell-free

WPBs maintain their typical elongated shape; scale bar, 10 µm. (H) STORM of cell-free WPBs centrifuged on coverslips to obtain 2D-constrained and well-separated organelles; scale bar, 500 nm.

Figure S2. Related to Figure 4**. Unlinked ministacks generate mini-WPBs**. (A) Cumulative frequency of the number of newly made WPBs (vWF-GFP labelled) as a function of organelle length. n: DMSO, 853; Nocodazole, 575. More than 95% of the WPBs produced by unlinked ministacks were ≤ 1 µm. #, $p < 10^{-10}$. (B) Lengths of Golgi elements (GM130- and TGN46-positive structures) and WPBs produced during nocodazole treatment. Medians and interquartile ranges are shown. n = 1530 and 1157 for GM130- and TGN46-positive objects, respectively. (C) Length frequency of vWF-GFP labelled WPBs generated by nocodazole treatment. (D) vWF-GFP STORM images (anti-EGFP antibody) of cells treated as in (A); scale bar, 500 nm. (E) Low pH/acetate treatment unlinks the Golgi ribbon without affecting the microtubule network (G, Golgi region; P, cell periphery). Scale bar, 10 µm; zoom-in, 5 µm. (F) The efficiency of individual or combined siRNA-mediated depletion of the three Golgi matrix proteins GM130, GRASP55 and Giantin was in the range of 80-95%. (G) Newly made WPBs (vWF-GFP) in control and triple GM130, GRASP55, Giantin knockdown (3x GM) in HUVECs. The Golgi matrix proteins were simultaneously stained with a mix of three specific antibodies; scale bar, 2 µm. (H) HTM analysis showed that 3x GM knockdown was as efficient as nocodazole in generating mini-WPBs. n = 1.27×10^6, 7.16×10^5 and 7.46×10^5 for Lucif./DMSO, 3xGM/DMSO and Lucif./Noco, respectively. ###, $p < 10^{-15}$. (I) Mini-WPBs generated by unlinked Golgi ministacks (nocodazole incubation) were ultrastructurally normal. Ministacks (GS), clathrin coats (hollow arrows), membrane continuities with the TGN (arrows) and vWF tubules (asterisks) are indicated; scale bar, 500 nm.

Figure S3. Related to Figure 4. **Effects of Rab6a/a' depletion on Golgi and WPB sizes.** (A) HTM analysis of the area of the TGN46-positive objects analyzed in Figure 4J. ###, $p < 10^{-15}$. (B) Golgi ribbons were unlinked with nocodazole into separated ministacks in order to facilitate cisternal length measurement from electron micrographs as previously described by (Trucco et al., 2004); scale bar, 500 nm. (C) Gaussian mixture models of WPB length clusters of Luciferase and Rab6a/a' siRNA-treated HUVECs. Rab6a/a' depletion shifted the clusters toward higher values. (D) HTM analysis of the length of nocodazole-induced mini-WPBs in Luciferase and Rab6a/a' siRNA-treated HUVECs. ###, $p < 10^{-15}$.

Figure S4. Related to Figure 6. **Size-dependent properties of WPBs *in vitro* and in cells.** (A) After nocodazole washout, the microtubule network reforms by 30 min, while Golgi ribbon reassembly is completed by 90 min; scale bar, 10 μm. (B) HTM analysis of WPB number per cell. Nocodazole treatment induced a minor reduction of the number of WPBs per cell at steady state. Median values are shown (n = 24; with each observation from a separate well of 96-well plates); ***, $p < 10^{-3}$. (C) Cell-free WPBs were treated in solution with control buffer or buffer supplemented with 10 mM dithiothreitol (DTT) to reduce multimer disulfide bonds. Treated WPBs were adsorbed by centrifugation on plastic, permeabilized and fixed. vWF filaments production was ablated by DTT incubation, indicating that vWF multimers are required for their formation. Inverted wide-field images are shown. Scale bar, 50 μm. (D) The frequency of filaments ≤ 20 μm produced by cell-free mini-WPBs was greatly increased. (E) Platelet-decorated vWF strings were not formed in the absence of secretagogue. In agreement with their increased levels of basal secretion (see Figure 6A), mini-WPB producing cells displayed higher vWF surface staining. Scale bar, 50 μm. (F) Density (number/HUVEC) of vWF-decorated platelet strings in samples stimulated with histamine. Box plots of data of 8 tile scans per treatment (with ~ 4 mm^2/tile scan) from 2 separate experiments; ***, $p < 10^{-3}$. (G) Basal and histamine-stimulated

rolling of THP-1 monocyte-like cells for the same experiments shown in Figure 6F. Histamine increased the rolling of monocytes to similar extent in control and mini-WPB enriched HUVECs. Note that there was no difference in basal levels of rolling between the treatments. Box plots of 12 determinations from 4 experiments; **, $p < 10^{-2}$; ***, $p < 10^{-3}$.

Figure S5. Related to Figure 7**. Ministacks and TGN in WPB biogenesis.** (A) NH_4Cl incubation/washout protocol was carried out in cells where the Golgi ribbon had been unlinked by nocodazole treatment. This allowed localization of vWF-GFP within the ministacks during WPB formation. Marker intensity across ministacks was measured in ImageJ with the “plot profile” tool (as shown for the confocal image, scale bar 0.5 µm, and exemplified by the diagram). Two examples each are reported for 30 and 120 min post NH_4Cl washout. Consistent with vWF quantum formation in the cisternae of the ministacks, at 30 min (early in WPB formation process; see Figures 3C-3E), vWF-GFP objects could be observed at locations corresponding to the cis- and median/trans-Golgi (shown plots) and the TGN (not shown). At 120 min (before WPB budding; see Figures 3C-3E), the most common location of vWF-GFP objects was at or protruding from the TGN. (B) EM Montage of the Golgi region of a HUVEC containing a portion of the TGN and seen “en face” (outlined by a light brown transparent mask). The continuity of the TGN is at the base of our model of VWF quanta co-packaging into forming WPBs. A ministack, connected to the ribbon and captured in transverse version (GS), a centriole (Ce) and WPBs are visible. Scale bar, 500 nm.

Table S1. Related to Figure 2.

**WPB length cluster**	**Range of Feret diameter analyzed**	**Cluster mean length (μm)**	**Cluster standard deviation (μm)**
1	< 2.5 μm	0.5	0.05
2	< 2.5 μm	1	0.18
3	< 2.5 μm	1.5	0.33
4	< 2.5 μm	2.2	0.15
5	2.5-4.0 μm	2.6	0.06
6	2.5-4.0 μm	2.9	0.16
7	2.5-4.0 μm	3.4	0.3
8	4.0-5.5 μm	4.1	0.08
9	4.0-5.5 μm	4.5	0.21
10	4.0-5.5 μm	5.1	0.22

Regularly spaced WPB length clusters.

WPB lengths cluster at values multiple of ~ 0.5 μm. Estimated means and standard deviations of the individual components of the Gaussian mixture (Figure 2B) calculated by Mclust.

Table S2. Related to Figure 5.

**Length interval (μm) 1.4-2.5**	**1st cluster mean (μm)**	**1st cluster St. Dev. (μm)**	**2nd cluster mean (μm)**	**2nd cluster St. Dev.**	**no. of WPBs in interval**
Control siRNA 200 pmol	1.55	0.09	1.97	0.25	212857
vWF siRNA 10 pmol	1.55	0.08	1.95	0.25	79580
vWF siRNA 50 pmol	1.54	0.08	1.92	0.25	45182
vWF siRNA 200 pmol	1.54	0.08	1.90	0.24	16095
**Length interval (μm) 1.9-3.1**	**1st cluster mean (μm)**	**1st cluster St. Dev. (μm)**	**2nd cluster mean (μm)**	**2nd cluster St. Dev. (μm)**	**no. of WPBs in interval**
Control siRNA 200 pmol	2.06	0.10	2.52	0.27	107686
vWF siRNA 10 pmol	2.06	0.09	2.50	0.27	34357
vWF siRNA 50 pmol	2.04	0.08	2.46	0.27	5613
vWF siRNA 200 pmol	2.06	0.08	2.50	0.27	456

vWF expression levels do not affect quantum size.

Estimated means and standard deviations of the two components of the Gaussian mixture calculated by Mclust for each siRNA treatment in the length intervals shown in Figure. 5E.

EXTENDED EXPERIMENTAL PROCEDURES

HUVEC growth medium (HGM). HUVECs were maintained in HGM, M199 (Gibco, Life Technologies) supplemented with 20% Fetal Bovine Serum, (Biosera), 30 µg/ml endothelial cell growth supplement from bovine neural tissue and 10 U/ml Heparin (both from Sigma-Aldrich).

Antibodies and reagents. Anti-vWF rabbit polyclonal (cat. no. A0082), rabbit-polyclonal HRP-conjugated (cat. no. P0226), and mouse monoclonal (clone F8/86), were from DAKO. Anti-GM130 mouse monoclonal, clone 35, was from BD Biosciences. Anti-TGN46 sheep polyclonal AHP500 was from AbD Serotec. Anti-GRASP55 rabbit polyclonal was from Proteintech. Anti-Giantin rabbit polyclonal (cat. no. Ab24568) was from Abcam. Anti-Rab6 rabbit polyclonal C-19 was from Santa Cruz Biotechnology. Anti-β-Tubulin mouse monoclonal (clone TUB 2.1) was from Sigma Aldrich. Anti-pan-Tubulin sheep polyclonal ATN02 was from Cytoskeleton. Anti-EGFP (mixture of two mouse monoclonal antibodies) was from Roche. Anti-CD41 mouse monoclonal (clone 5B12) was from Millipore. All reagents were from Sigma-Aldrich unless otherwise specified.

Plasmids and siRNAs. siRNA were custom synthesized (Eurofins MWG Operon). Targets and siRNA sequences:
Firefly Luciferase, sense, 5'-CGUACGCGGAAUACUUCGA[dT][dT]-3';
hvWF, sense, 5'-GGGCUCGAGUGUACCAAAA[dT][dT]-3';
hGM130, sense, 5'- AAGUUAGAGAUGACGGAACUC [dT][dT]-3';
hGRASP55, sense, 5'- AACUGUCGAGAAGUGAUUAUU[dT][dT]-3'
hGiantin, sense, 5' - AACUUCAUGCGAAGGCCAAAU [dT][dT]-3';
hRab6a/a', sense, 5'- GAGAAGAUAUGAUUGACAU[dT][dT] (targets both Rab6a and Rab6a' isoforms). All siRNAs were described and validated in other studies (Elbashir et al., 2001; Feinstein and Linstedt, 2008; Heuer et al., 2009; Puthenveedu et al., 2006; Starke et al., 2011; Sun et al., 2007). Plasmids encoding vWF-GFP, a gift from J. Voorberg and J.A. Van Mourik, and EGFP-Rab27A were previously described (Hannah et al., 2003; Romani de Wit et al.,

2003). Plasmid DNA was nucleofected at 4 to 7.5 µg per reaction. Cells treated with siRNAs targeting vWF and Luciferase were analyzed at 48 except in the case of time course experiments. siRNAs were delivered at 200 pmol per reaction or as indicated together with EGFP-Rab27A plasmid when the latter was used. siRNAs to GM130, GRASP55, Giantin, Rab6a/a' (and Luciferase as control) were used at 1000 pmol per reaction and a second round of nucleofection at 48 h was carried out. Cells were analyzed 24 h after the second nucleofection round. When vWF-GFP was used to label newly made WPBs, it was nucleofected at the time of the second round.

Stochastic Optical Reconstruction Microscopy (STORM). Fluorescence and excitation lights were spectrally separated by a full multi-edge filter set (LF405/488/561/635-A-000, Semrock). A diode laser operating at 640 nm (iBeam smart, 150mW, Toptica Photonic AG) was fibre coupled, expanded and focused at the edge of the back focal plane of the objective (UAPON 100xOTIRF, NA=1.49). The beam size of the excitation laser was matched to the field of view of the imaging system limited by the detector chip size (16µm/pixel*512pixel). The power density of the excitation laser on the sample was approximately 2 kW/cm^2. For data acquisition, a ROI (128×128 pixels) with the highest and most homogeneously illuminated area was used. Image stack files of fluorophore "blinking" events (composed of 10000 to 20000 frames) were captured using a low-noise, highly sensitive electron-multiplying EMCCD camera (Andor iXon 897, Belfast, UK) at frames rates of 54 per second with 10 ms exposure times. Fluorophores (AlexaFluor 647 or Cy5) were reactivated by a 405 nm laser (Mitsubishi Electronics Corp.) at low power density (<50 W/cm^2). Samples were imaged in a photo-switching buffer containing 50 µg/ml (5 units) glucose oxidase, 1 µg/ml (40-60 units) catalase and 100 mM mercaptoethylamine-HCl (Metcalf et al., 2013). Images were reconstructed using the rainSTORM algorithm (http://laser.cheng.cam.ac.uk/wiki/index.php/Resource (Metcalf et al., 2013; Rees et al., 2012), with super-resolution pixel sizes of 20 nm. Mean localization precisions were around 25 nm, giving an estimated resolution limit of 50-60 nm

(Rees et al., 2012). STORM data is displayed by histogram visualization; i.e. accepted localizations were assigned to 20 nm pixels, each localization corresponding to 1 grey scale intensity value. For clarity of viewing a "red hot" lookup table and a contrast enhancement were applied (0.01-0.1% pixel saturation) in ImageJ.

Image analysis. *Confocal microscopy*. Maximum intensity projections were analyzed in ImageJ (http://imagej.nih.gov/ij/). Background intensity in vWF-GFP channel was measured and used for threshold setting. The "Find Maxima" tool was used to identify positive objects and the image subjected to watershed segmentation, separating nearby objects. Segmentation was then superimposed on the binary image obtained after threshold application. Selection of a series of objects was carried out to identify the area of the smallest, which was set as a cut-off in the parameter of the "Analyze Particles" tool. The same procedure was applied for quantifications of Golgi compartments (i.e. GM130- and TGN46-positive objects).

STORM. Reconstructed images were analysed in ImageJ. vWF quanta were identified using the "Find maxima" tool and separated by watershed segmentation. Segmentation was applied to binary images of the reconstructions to separate the quanta within the same organelle. The "Analyze Particles" tool was used to measure the Feret diameter of each separated quantum.

High-throughput confocal microscopy and morphometric analysis (High-throughput morphometry, HTM). A high performance barebone computer system equipped with 256 GB RAM and four dodeca-core CPUs running at 2.2 GHz, allowing parallelization of computation, was used for image processing. The system was running a 64-bit version of Ubuntu Linux. Image processing was performed ImageJ program (Abramoff et al., 2004) version 1.45l (http://imagej.nih.gov/ij/) with Java 1.6.0 using 111 gigabytes of heap size and enabling the incremental Java garbage collector. Flex format image files were converted to Tagged Image Format (TIF). The processing steps were executed

as a custom-built ImageJ macro, processing a 96-well plate image dataset in a single ImageJ instance. The macro performed the following steps.

Pre-processing. Images of a whole 96 well plate were imported in ImageJ as an image sequence resulting in a 16-bit stack. The stack was converted into a multi-channel hyperstack. The pixel width and height was calibrated to 0.1615 µm (40x lens with no camera binning). The vWF channel (labelling Weibel-Palade bodies) was subjected to noise filtering. Endoplasmic reticulum vWF pool was an inherent source of noise and was removed by applying a rolling ball algorithm (Sternberg, 1983) with a paraboloid curvature radius of 1 or 5 pixels depending of the noise level.

Segmentation. The segmentation process of WPBs had to be able to (i) find contours of both strongly and weakly fluorescent WPBs and (ii) separate closely apposed WPBs. Global segmentation algorithms that calculate a single threshold for a whole image were not amenable to these criteria. Instead, a local segmentation was applied where threshold was calculated as a function of the coordinates at each pixel. The ImageJ implementation of the Bernsen (Bernsen, 1986) locally adaptive threshold algorithm is based on the local contrast around a central pixel. We used an experimentally determined 5-pixel radius circular window around the central pixel, as it was found to be the optimal parameter with respect to WPB size. The default contrast threshold level (set at 15) was used. An overlay image was generated automatically for quality control purpose for every segmented WPB image, where WPB contours were superimposed on the original image. The feature extraction step in the high-content analysis workflow allowed us to reduce the image dataset stored as pixels to more geometrically interpretable measurements, such as WPB area and Feret diameter (also known as maximum caliper), which is the longest distance between any two opposite points along the perimeter of an object. In the case of a WPB this distance approximates its major axis, and therefore its length. Despite reducing the dataset magnitude from hundreds of gigabytes to megabytes, this still led to a multidimensional space where a WPB was described by 22 measured values. During the feature selection step, area and Feret diameter were chosen to be the

most salient features to define a WPB. The objects thus identified were filtered based on size, cutting off those with sizes ≤ 2 pixels (approximately 300 nm), which visually did not conform to WPB morphology and likely represent vWF vesicular carriers.

Statistical data analysis. For the initial survey, WPB objects were measured (n = 1976161, from cells grown in 432 wells of 96-well plates) and the quantified features were saved into large text files. We employed the open source statistical and analytical tool ***R*** (http://r-project.org) (Team, 2009) for data analysis. Visual inspection indicated that WPB length frequency distribution was multi-modal, presenting local maxima (that is, preferred lengths or length clusters). We hypothesized that WPB length distribution could be modelled by a mixture of Gaussian functions. To estimate the positions of the length clusters, we used model-based clustering to fit a Gaussian mixture model as a density estimate for our dataset. Using the ***R*** package “Mclust” version 4 (Fraley and Raftery, 2002) that fits a mixture of Gaussian functions, we performed cluster analysis and calculated the univariate density and uncertainty. The assumed number of mixture components (clusters) of the Mclust function were chosen as G = 4, which was found as the optimal number of mixture components with unequal variance model for lengths < 2.5 µm. G = 3 with unequal variance model was optimal for the 2.5-4.0 µm and 4.0-5.5 µm length intervals. All other parameters were used as default. The mean and standard deviation of each cluster were calculated (see Supplementary Table 1). The ‘mclust1Dplot’ function was also used to calculate and plot, from the original length dataset, the density and uncertainty (misclassification) for value attribution to neighbouring clusters. The most uncertain (misclassified) values were located at length intermediate between two clusters. ***R*** was also used to analyze all the other experimental high-throughput imaging datasets.

Golgi (TGN46-positive) elements were subjected essentially to the same processing and segmentation as described for WPBs. Lengths (Feret diameters) and areas of the segmented Golgi elements were analyzed with ***R***.

NH_4Cl incubation/washout. HUVECs were treated with 10 mM NH_4Cl in growth medium for the times described to prevent generation of WPBs at the Golgi (Wagner et al., 1986). Resumption of WPB formation was obtained washing out NH_4Cl by extensive rinsing with ice-cold HGM or serum-free (SF) medium (M199 supplemented with 10 mM HEPES-NaOH, pH 7.4 and 0.1 mg/ml BSA, followed by incubation in media at 37 °C for the indicated times.

Golgi ribbon unlinking. Cells were incubated with nocodazole, diluted in HGM to 1 µg/ml (from a 10 µg/ml stock in DMSO) for 18-20 h. HGM supplemented with DMSO (0.01% final concentration) was used as a control. In a second approach, we adopted the protocol described by (Yoshida et al. 1999) with slight modifications. HEPES-NaOH, pH 7.3, from a 1 M solution, was added to HGM (final pH of ~ 7.3) to a final concentration of 30 mM (control). 2-(N-Morpholino)ethanesulphonic acid (MES) and sodium acetate, both from 1 M stocks, were added to 30 and 25 mM final concentrations, respectively. The pH of this medium was ~ 6.4. Cells were grown in these media for 24 h before being processed for immuno-fluorescence.

Golgi ribbon unlinking during WPB formation. Cells were nucleofected with vWF-GFP and incubated with NH_4Cl overnight as described above. To allow formation of WPBs, NH_4Cl was washed out and cells were chased with medium supplemented with DMSO. To unlink the Golgi ribbon at the indicated times, cells were transferred on ice for 30 min to depolymerize microtubules and then chased in nocodazole at 37 ºC for the indicated times before being processed for immuno-fluorescence.

vWF imaging at the Golgi during WPB formation. Cells were nucleofected with vWF-GFP and incubated overnight with both NH_4Cl and nocodazole at the concentrations described above. NH_4Cl was then washed out and cells chased for the indicated times in the continued presence of nocodazole before being processed for immuno-fluorescence. Fluorescence maxima for vWF-GFP and

the Golgi markers GM130 (cis-Golgi) and TGN46 (TGN) were identified with ImageJ's "plot profile" tool, in order to localize the WPB marker in the context of the ministacks.

Western blotting. Cells were lysed on ice with the following buffer: 100 mM Tris-HCl, pH 7.5/150 mm NaCl/1% TX-100/0.5% Na-Deoxycholate/0.05% SDS, supplemented with protease inhibitor cocktail (Sigma-Aldrich). Lysates were clarified by centrifugation and protein concentration measured by bicinchoninic acid assay (Pierce). Equal protein amounts were fractionated by SDS-PAGE in denaturing and reducing conditions and electro-blotted on PVDF membranes. After blocking with 5% BSA in 0.05% Tween-20 PBS (PBS-T), membranes were incubated with primary antibodies diluted in 5% BSA/PBS-T, followed by HRP-conjugated secondary antibodies. Chemiluminescent signals were acquired in digital format with ImageQuant LAS 4000 (GE Healthcare Life Sciences) Signals were quantified in ImageJ; pan-tubulin signal was used for normalization.

vWF ELISA and multimer analysis. vWF content in cell lysates, releasates and fractions form gradients was measured using a sandwich ELISA, as previously described (Blagoveshchenskaya et al., 2002). vWF multimers were fractionated by agarose gel electrophoresis, electro-blotted on PVDF and detected as described in (Nightingale et al., 2009).

Secretion assays. After 5 rinses in SF medium (5 incubations, each for 3-4 min) cells were assayed in the same medium. vWF releasates in the absence (basal) or the presence of the secretagogue histamine (100 μM), each for 30 min at 37 ºC, were quantified by ELISA and expressed as percent of total vWF content (obtained by adding the amounts of vWF in lysates and releasates). Cells treated with DMSO and nocodazole were subjected to washout 1 h before assay to allow complete reassembly of the microtubule network.

Preparation of cell-free WPBs. HUVECs were homogenized on ice in 250 mM sucrose/5 mM Na_2-EDTA/20 mM HEPES-NaOH, pH 7.4, supplemented with protease inhibitor cocktail. Homogenates were centrifuged at 1000 g for 5 min (at 4 °C). The resulting in post-nuclear supernatants were loaded on 15%-40% Nycodenz (Axis-Shields) cushions (both in homogenization buffer) and centrifuged at 18000 x g and 4 °C for 30 min with MLS50 (Beckman Coulter) in an Optima™ Max ultracentrifuge. Cell-free WPBs were recovered at the 15%-40% Nycodenz interface.

vWF filaments generated from cell-free WPBs. vWF-silenced (for 48 h), DMSO and nocodazole treated (both for 18-20 h) HUVECs were processed to obtain cell-free WPBs as described. Fractions containing WPBs (equal vWF amounts by ELISA) were serially diluted in 96-well Maxisorp™ plates (NUNC, Thermo Scientific) and centrifuged (2000 r.p.m. with a GH3.8 rotor, Beckman Coulter) for 30 min at 4 °C. Surface-adsorbed membranes were permeabilized (30 min on ice with 1% Triton X-100 in homogenization buffer) and fixed with 4% formaldehyde in the same buffer. After immuno-staining, vWF filaments were imaged by wide-field microscopy with a 20x objective lens (NA 0.4) on a Leica DM IRB microscope. vWF string lengths were measured using ImageJ's "segmented line" tool.

Platelet-decorated vWF strings generation and analysis. HUVECs were seeded in flow chambers (μ-Slide I, Ibidi) to produce confluent monolayers and then fed with growth medium supplemented with either vehicle (DMSO) or 1 μg/ml nocodazole to obtain control and mini-WPB enriched cells. After 20 h culture, cells were extensively rinsed to remove DMSO or nocodazole and incubated at 37 °C in growth medium for 45 minutes before being connected to a pump system (Harvard Apparatus, Holliston, MA, USA). Hanks buffer (containing Ca^{2+}, $Mg^{2+}$and 0.2 % BSA) pre-warmed at 37 °C was perfused for a few minutes to rinse out the growth medium and loosely attached cells. Then, the same buffer supplemented with 100 μM histamine was perfused for 1 min alone and then with

platelets (freshly prepared as described in (Michaux et al., 2006), at a concentration of 0.5-1 x 10^8 per ml) for 5 min. A constant shear stress of 0.25 MPa (2.5 dynes/cm^2) was maintained throughout the experiment. In some experiments histamine was omitted throughout the perfusion in order to visualize the formation of strings in basal conditions. Cells were then fixed under flow with 4% formaldehyde in PBS for 5 min, gradually reducing the flow rate to zero. Cells were not permeabilized and extracellular pools of vWF (to visualize strings) and GPIIb/CD41 (to visualize platelets) were immuno-stained; DAPI (Life Technologies) was used to label the nuclei. Stacks of confocal images were acquired from 20 adjacent fields of view and stitched together into tilescans. Maximum intensity projections of the tilescans were used for quantification of length and density of vWF-decorated platelet string (i.e. number of strings/nuclei in each tilescan) with ImageJ, using the “straight line” tool. Platelet string length was defined as the maximum distance between platelets decorating an underlying vWF filament.

Leukocyte rolling assays. HUVECs (seeded on µ-slides VI, Ibidi) were essentially prepared as described for the platelet string assays. At the time of assay, flow chambers were transferred to the microscope stage of an Axiovert 100 (Carl Zeiss, Welwyn Garden City, UK) maintained at 37 °C and imaged using a 10x objective. After a brief perfusion with Hanks buffer (supplemented as described for the platelet string assays), THP-1 monocytes (10^6 cells/ml in Hanks buffer) were perfused at a shear stress of 0.07 Pa (0.7 dyne/cm^2) on the endothelial monolayers for a 3 min period to measure basal rolling. HUVECs were then perfused (in the absence of THP-1s) with buffer supplemented with 100 μM histamine for 5 min, followed by THP-1 (10^6 cells/ml) suspended in Hanks buffer devoid of secretagogue for a 3 min period. The rolling on the same field of view, in basal and stimulated conditions, was imaged by video-microscopy (at 24 frames/sec, using a QIMAGING Scientific CMOS Rolera bolt camera). Only THP-1 cells that rolled on the HUVEC monolayer at speeds ≤ 40 µm/sec were counted. To minimize the variability between experiments carried

out on different days, the number of rolling cells were normalized to the number of HUVECs imaged in the analyzed field of view and expressed as fraction of the mean rolling for histamine-stimulated controls in that day/experiment.

3D reconstruction from fixed cells. Confocal image stacks of fixed HUVECs, labelled with DAPI and antibodies to the appropriate organelle marker, were imported in Volocity (Pelkin Elmer) or IMARIS (Bitplane) for tri-dimensional rendering of nuclei, WPBs and Golgi regions.

Live cell imaging. HUVECs were nucleofected with vWF-GFP plasmid, seeded on gelatine coated glass bottom imaging dishes (PAA laboratories GmbH) and cultured overnight in growth medium. To label the Golgi Complex, cells were rinsed three times with serum-free medium and then incubated with 5 μM BODIPY Ceramide (Molecular Probes, Life Technologies) in serum-free medium for 10 min at 37 ºC. Cells were then washed three times in serum-free medium and incubated for 30 more min in growth medium at 37 ºC before imaging. Image acquisition was carried out with an Ultra*VIEW* VoX spinninig disk confocal system (Perkin Elmer) equipped with temperature and CO_2 controls (set at 37 ºC and 5%, respectively). Stacks of images (*z*-step, 0.25 μm), encompassing the entire cell volume, were sequentially acquired for the ceramide and the GFP channels at a rate of 1 stack every 10 sec for 20-150 min. Image sequences were imported in IMARIS (Bitplane Scientific Software) for object tracking and 3D-rendering.

SUPPLEMENTAL REFERENCES

Abramoff, M.D., Magalhães, P.J., and Ram, S.J. (2004). Image processing with ImageJ. Biophotonics international *11*, 36-42.

Bernsen, J. (1986). Dynamic Thresholding of Grey-Level Images. Paper presented at: 8th Int Conf on Pattern Recognition (ICPR8) (Paris, France).

Blagoveshchenskaya, A.D., Hannah, M.J., Allen, S., and Cutler, D.F. (2002). Selective and signal-dependent recruitment of membrane proteins to secretory granules formed by heterologously expressed von Willebrand factor. Molecular biology of the cell *13*, 1582-1593.

Elbashir, S.M., Harborth, J., Lendeckel, W., Yalcin, A., Weber, K., and Tuschl, T. (2001). Duplexes of 21-nucleotide RNAs mediate RNA interference in cultured mammalian cells. Nature *411*, 494-498.

Fraley, C., and Raftery, A.E. (2002). Model-based clustering, discriminant analysis, and density estimation. J Am Stat Assoc *97*, 611-631.

Hannah, M.J., Hume, A.N., Arribas, M., Williams, R., Hewlett, L.J., Seabra, M.C., and Cutler, D.F. (2003). Weibel-Palade bodies recruit Rab27 by a content-driven, maturation-dependent mechanism that is independent of cell type. Journal of cell science *116*, 3939-3948.

Metcalf, D.J., Edwards, R., Kumarswami, N., and Knight, A.E. (2013). Test samples for optimizing STORM super-resolution microscopy. J Vis Exp.

Rees, J.E., Erdelyi, M., Pinotsi, D., Knight, A., Metcalf, D., and Kaminski, C.F. (2012). Blind assessment of localization microscope image resolution. Optical Nanoscopy *1*.

Romani de Wit, T., Rondaij, M.G., Hordijk, P.L., Voorberg, J., and van Mourik, J.A. (2003). Real-time imaging of the dynamics and secretory behavior of Weibel-Palade bodies. Arterioscler Thromb Vasc Biol *23*, 755-761.

Starke, R.D., Ferraro, F., Paschalaki, K.E., Dryden, N.H., McKinnon, T.A., Sutton, R.E., Payne, E.M., Haskard, D.O., Hughes, A.D., Cutler, D.F., *et al.* (2011). Endothelial von Willebrand factor regulates angiogenesis. Blood *117*, 1071-1080.

Sternberg, S.R. (1983). Biomedical Image Processing. Computer *16*, 22-34.

Sun, Y., Shestakova, A., Hunt, L., Sehgal, S., Lupashin, V., and Storrie, B. (2007). Rab6 regulates both ZW10/RINT-1 and conserved oligomeric Golgi complex-dependent Golgi trafficking and homeostasis. Molecular biology of the cell *18*, 4129-4142.

Team, R.D.C. (2009). R: A language and environment for statistical computing (Vienna, Austria, R Foundation for Statistical Computing).

Trucco, A., Polishchuk, R.S., Martella, O., Di Pentima, A., Fusella, A., Di Giandomenico, D., San Pietro, E., Beznoussenko, G.V., Polishchuk, E.V., Baldassarre, M., *et al.* (2004). Secretory traffic triggers the formation of tubular continuities across Golgi sub-compartments. Nat Cell Biol *6*, 1071-1081.
