# Supplementary figures and images for: A Two-Tier Golgi-Based Control of Organelle Size Underpins the Functional Plasticity of Endothelial Cells
Source: Dev Cell. 2014 May 12;29(3):292–304. doi: 10.1016/j.devcel.2014.03.021 (PMC4022834; doi:10.1016/j.devcel.2014.03.021)

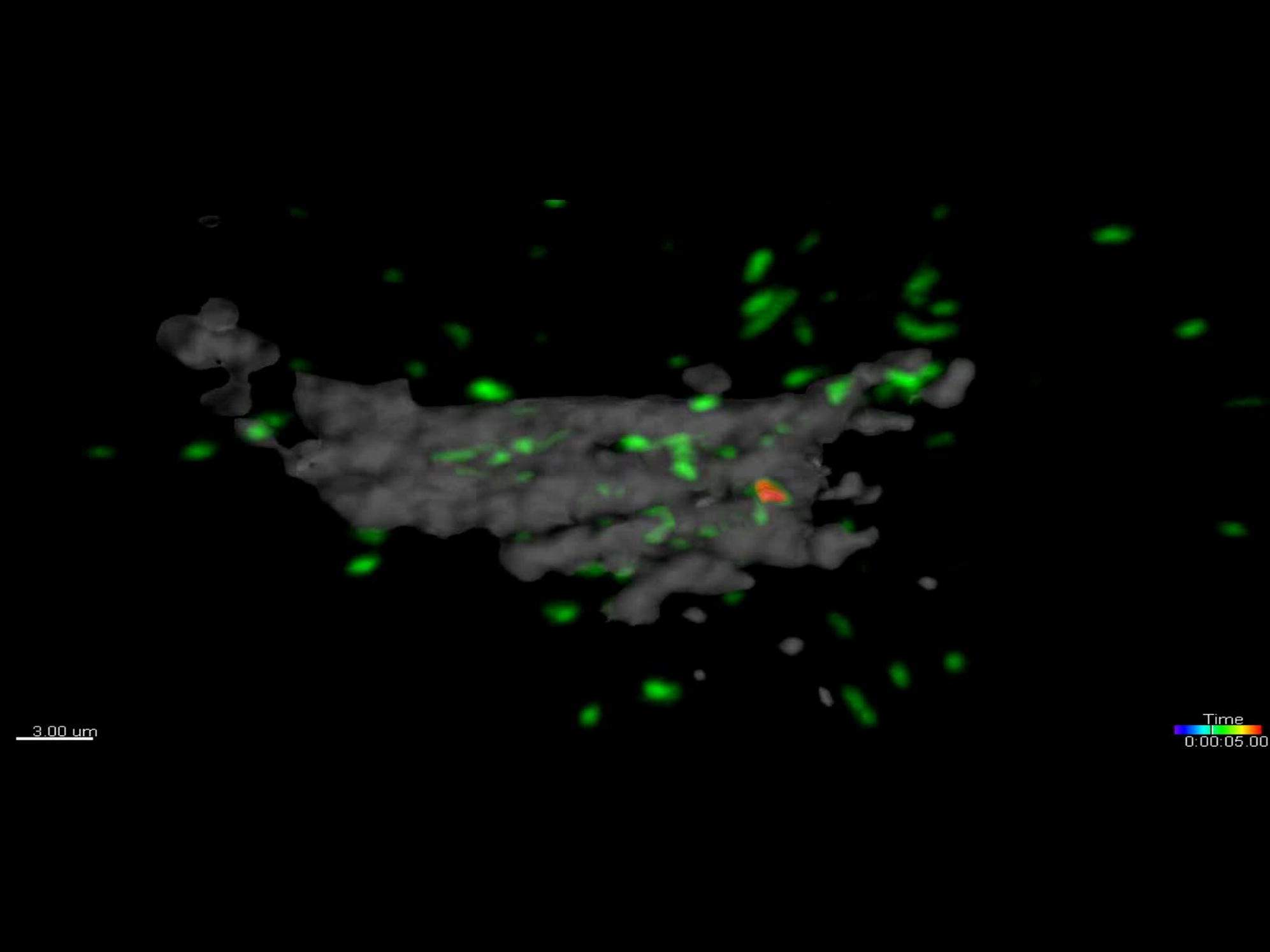

Supplement: Movie S1. vWF-Positive Objects Merge within the Golgi to Generate a WPB, Related to Figure 3 — HUVECs were nucleofected with vWF-GFP plasmid and incubated with BODIPY ceramide to label the Golgi apparatus. Image stacks were acquired every 10 s and 3D reconstructed with IMARIS. Two vWF-GFP-positive objects (highlighted in orange) are seen to merge within the volume of the Golgi apparatus (rendered in transparent gray) and move to the cell periphery as a single object. [file mmc2.jpg]
