# Supplementary material for: A Two-Tier Golgi-Based Control of Organelle Size Underpins the Functional Plasticity of Endothelial Cells
Source: Dev Cell. 2014 May 12;29(3):292–304. doi: 10.1016/j.devcel.2014.03.021 (PMC4022834; doi:10.1016/j.devcel.2014.03.021)
Supplement: Document S2. Article plus Supplemental Information [file mmc3.pdf]

Developmental Cell
Article

# A Two-Tier Golgi-Based Control of Organelle Size Underpins the Functional Plasticity of Endothelial Cells

Francesco Ferraro,[1] Janos Kriston-Vizi,[2,3] Daniel J. Metcalf,[4] Belen Martin-Martin,[1] Jamie Freeman,[2] Jemima J. Burden,[5] David Westmoreland,[1] Clare E. Dyer,[1] Alex E. Knight,[4] Robin Ketteler,[2] and Daniel F. Cutler[1,*]
[1]Endothelial Cell Biology Laboratory, Laboratory for Molecular and Cellular Biology, University College London, Gower Street, London WC1E 6BT, UK
[2]Translational Research Resource Center, Laboratory for Molecular and Cellular Biology, University College London, Gower Street, London WC1E 6BT, UK
[3]Bioinformatics Image Core, Laboratory for Molecular and Cellular Biology, University College London, Gower Street, London WC1E 6BT, UK
[4]Analytical Science Division, National Physical Laboratory, Hampton Road, Teddington, Middlesex TW11 0LW, UK
[5]Electron Microscopy Laboratory, Laboratory for Molecular and Cellular Biology, University College London, Gower Street, London WC1E 6BT, UK
*Correspondence: d.cutler@ucl.ac.uk
http://dx.doi.org/10.1016/j.devcel.2014.03.021
This is an open access article under the CC BY license (http://creativecommons.org/licenses/by/3.0/).

## SUMMARY

**Weibel-Palade bodies (WPBs), endothelial-specific secretory granules that are central to primary hemostasis and inflammation, occur in dimensions ranging between 0.5 and 5 μm. How their size is determined and whether it has a functional relevance are at present unknown. Here, we provide evidence for a dual role of the Golgi apparatus in controlling the size of these secretory carriers. At the ministack level, cisternae constrain the size of nanostructures ("quanta") of von Willebrand factor (vWF), the main WPB cargo. The ribbon architecture of the Golgi then allows copackaging of a variable number of vWF quanta within the continuous lumen of the *trans*-Golgi network, thereby generating organelles of different sizes. Reducing the WPB size abates endothelial cell hemostatic function by drastically diminishing platelet recruitment, but, strikingly, the inflammatory response (the endothelial capacity to engage leukocytes) is unaltered. Size can thus confer functional plasticity to an organelle by differentially affecting its activities.**

## INTRODUCTION

Discovered five decades ago (Weibel and Palade, 1964), Weibel-Palade bodies (WPBs) are endothelial-specific secretory granules that are fundamental to the initiation of hemostatic and inflammatory responses. WPBs store endothelial von Willebrand factor (vWF), a large glycoprotein that undergoes complex processing along the secretory pathway (Metcalf et al., 2008). Synthesized as a proprotein, vWF dimerizes in the ER and is proteolytically cleaved in the Golgi apparatus, generating the propeptide and mature vWF forms, which remain tightly associated (Figures 1A–1C). The acidic milieu of the Golgi lumen is needed for cleavage, conformational changes, and self-assembly (Huang et al., 2008; Zhou et al., 2011). These processes are required for VWF's compact packaging into tubules visible by electron microscopy (EM) (Weibel and Palade, 1964; Zenner et al., 2007), as well as its multimerization via extensive interchain disulfide bonding between mature dimers (Mayadas and Wagner, 1989; Figures 1C–1E). At the *trans*-Golgi network (TGN), vWF is finally packaged into nascent WPBs, which are 200–300 nm wide and have lengths in the micrometer range, through a process requiring the cytosolic clathrin/AP-1 coat machinery (Lui-Roberts et al., 2005; Metcalf et al., 2008).

vWF is fundamental to primary hemostasis. Upon vessel injury, WPBs undergo exocytosis, and the coiled vWF multimers, with masses reaching 20 MDa (Figures 1D and 1E), unfurl in the direction of blood flow, ultimately forming strings that can extend hundreds of micrometers. These strings bind circulating platelets, promoting their aggregation into a loose plug, the first step to curb hemorrhage. Quantitative and/or qualitative deficiencies in secreted vWF multimers lead to the most common bleeding disorder in humans, von Willebrand's disease, which is estimated to affect up to 1% of the population (Sadler, 1998), while elevated serum vWF levels are associated with cardiovascular pathologies (van Galen et al., 2012). vWF is also involved in angiogenesis and inflammation and has been linked to atherosclerosis (Methia et al., 2001; Petri et al., 2010; Starke et al., 2011). We previously established that the typical elongated shape of WPBs reflects the lumenal arrangement of vWF tubules and is optimal for the organelle's hemostatic role (Michaux et al., 2006a). However, how WPBs of vastly different sizes are generated and whether this is reflected in their physiology remain unknown. Here, we addressed these questions.

## RESULTS

### A Length Unit for WPBs

WPBs are present in different sizes ranging between 0.5 and 5 μm (Weibel and Palade, 1964). To gain insight into possible

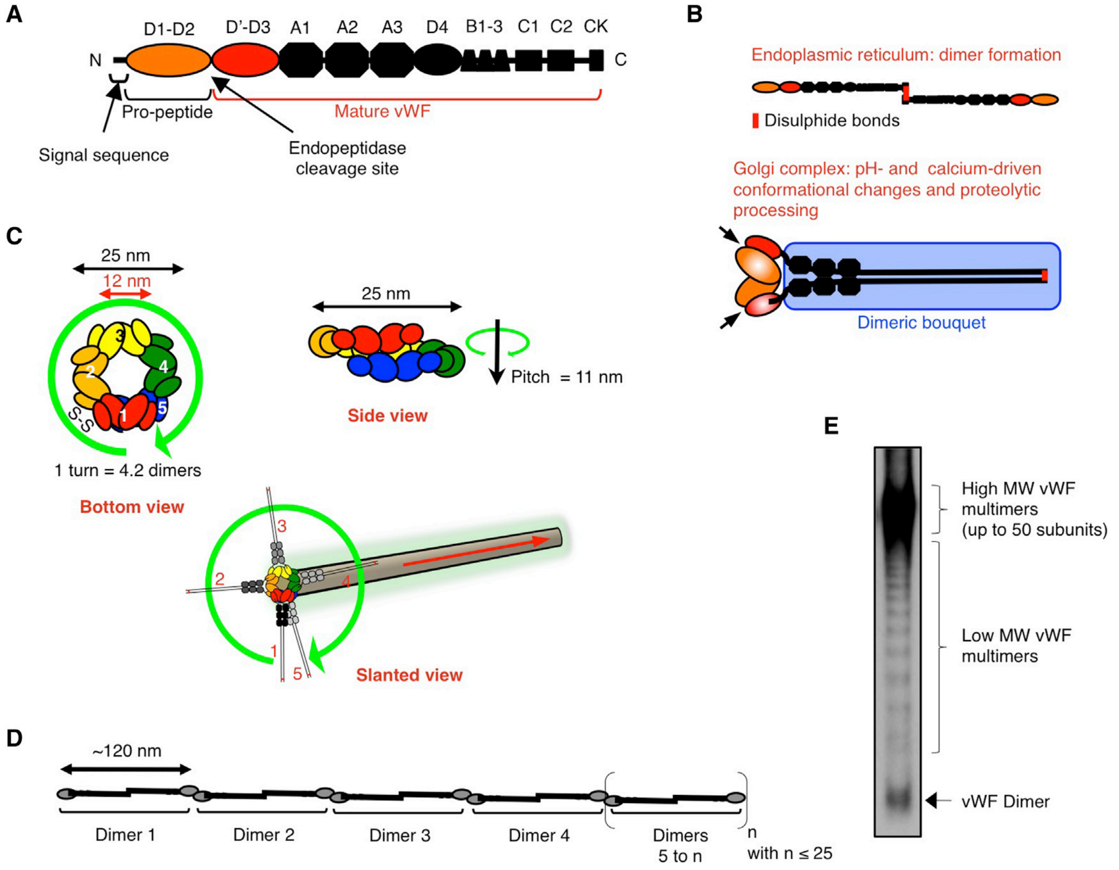


**Figure 1. Biosynthesis and Structural Features of vWF**

(A) Diagram of the domain composition of prepro-vWF (Metcalf et al., 2008; Sadler, 1998).

(B) Disulfide bonds formation in the ER generates pro-vWF dimers, and in the Golgi lumen, acidic pH and calcium promote the "dimeric bouquet" (blue boxed region) conformation (Zhou et al., 2011). Golgi's acidic milieu is also required for proteolytic processing (arrows), likely by furin, which generates the prodomain and mature vWF.

(C) At the Golgi, calcium and low pH promote vWF tubulation. A diagram of the spatial arrangement of five dimers (numbered 1–5) assembled into a tubule is shown. The propeptide (D1-D2) and the D'-D3 domains of each dimer are arranged into a right-hand helix forming the wall of the tubule (Huang et al., 2008). The helix has a period of 4.2 dimers and a pitch of 11 nm (for clarity, domains A1-CK are omitted in "bottom" and "side" views). In tubules, the configuration of D'-D3 domains of adjacent dimers is favorable to interdimer disulfide bond formation (S-S in bottom view) required for vWF multimerization.

(D) Upon exocytosis, the shift to neutral pH disrupts propeptide/D'-D3 interactions and the bouquet conformation, leading to the extension of multimerized vWF.

(E) vWF multimer analysis of an endothelial cell fraction containing WPBs (see Supplemental Experimental Procedures and Figure S1G). From bottom to top, bands visualize dimers, tetramers, hexamers, etc. In WPBs, the high-molecular-weight multimers contribute the majority of multimerized vWF.

mechanisms of size acquisition, we carried out a high-throughput microscopic survey on cultured human umbilical vein endothelial cells (HUVECs) stained for vWF to label these organelles. Due to their elongated shape (Figure 2A), changes in WPB size could be expressed as organelle length, an easily quantifiable parameter (see Supplemental Experimental Procedures and Figures S1A–S1C available online). Under our culture conditions, long WPBs represented a minority of the total population but contained a significant amount of vWF (Figure S1D, "number" versus "area"), a possible indication that long WPBs might be physiologically more important than suggested by their number. Most strikingly, our survey uncovered that WPB lengths cluster around values regularly spaced at ~0.5 μm intervals (Figures 2B and S1E; Table S1). The periodic occurrence of "preferred" organelle lengths suggested the existence of a "length unit" for WPBs, with the number of units determining the size of individual organelles.

Since vWF expression is necessary for the formation of WPBs (Denis et al., 1998; Wagner et al., 1991) (see Figures 5A and 5B), we tested whether it also accounted for the "unit" identified in our morphometric survey. Nanoscopic imaging of vWF immunoreactivity in HUVECs by stochastic optical reconstruction microscopy (STORM), a superresolution technique (Fölling et al., 2008; Rust et al., 2006), showed that this cargo displayed localization clusters discretely spaced along the length of each organelle (Figures 2C, arrowheads, and S1F). These localization clusters were also observed using a monoclonal antibody to vWF (data not shown) and were not artifacts due to optical sectioning, since cell-free WPBs lying flat showed the same vWF pattern (Figures S1G and S1H). vWF is thus organized within WPBs into nanoscopic clusters whose median size

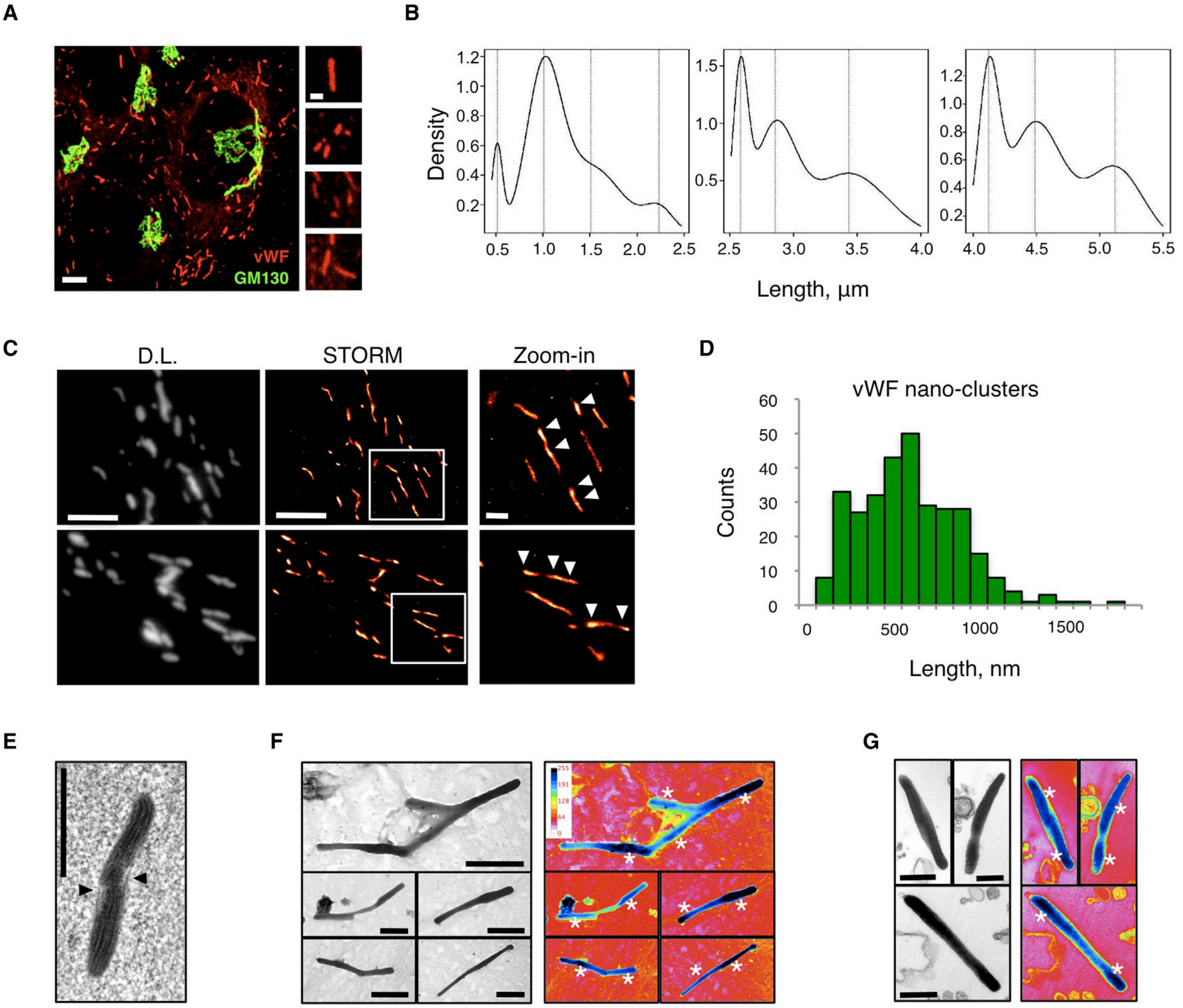


**Figure 2. A Length Unit for WPBs**

(A) WPBs (vWF) and the Golgi (GM130) were visualized in HUVECs; scale bar, 5 µm. Right: magnified regions exemplify WPB length variability; scale bar, 1 µm.

(B) High-throughput microscopic survey; the lengths of ~2 million WPBs were measured and modeled to a mixture of Gaussian distributions (see Supplemental Experimental Procedures).

(C) Diffraction-limited (DL) images of vWF-labeled HUVECs and their STORM reconstructions. Scale bar, 2 µm; zoom, 1 µm. Arrowheads indicate vWF nanoclusters within WPBs.

(D) vWF nanocluster size frequency was quantified from STORM images; n = 312; median [quartiles] = 576 [382, 797] nm.

(E) Electron micrograph from an HPF/FS HUVEC sample of a WPB (membrane continuities, arrowheads) showing two distinct regions.

(F and G) TEM of cell-free WPBs chemically fixed and prepared for whole-mount (F) or thick sections (G). Gray levels were color-coded to highlight variation in content density (denser regions labeled by asterisks). Scale bars in (E)–(G), 500 nm.

See also Figure S1 and Table S1.

correlates to the length unit inferred from high-throughput confocal morphometry (HTM; Figure 2D). Since vWF nanoclusters likely are the physical basis of the "length unit" uncovered by HTM and thus the origin of the WPBs' discrete sizes (Figure 2B), we called them "quanta."

vWF is the most abundant WPB cargo (Ewenstein et al., 1987); therefore, at the ultrastructural level, an uneven distribution of vWF within WPBs might be reflected by variations in the organelle content. Electron micrographs of WPBs, imaged within cells or isolated, were consistent with this prediction and displayed regions of higher electron density similar in size to that observed for the vWF quantum (Figures 2E–2G).

## WPB Length Is Determined at the Golgi

The observation of long WPBs at or in close proximity to the Golgi (Figure 3A) is consistent with the notion that their formation is complete before they bud from the TGN (Lui-Roberts et al., 2005; Zenner et al., 2007). However, our findings raised the

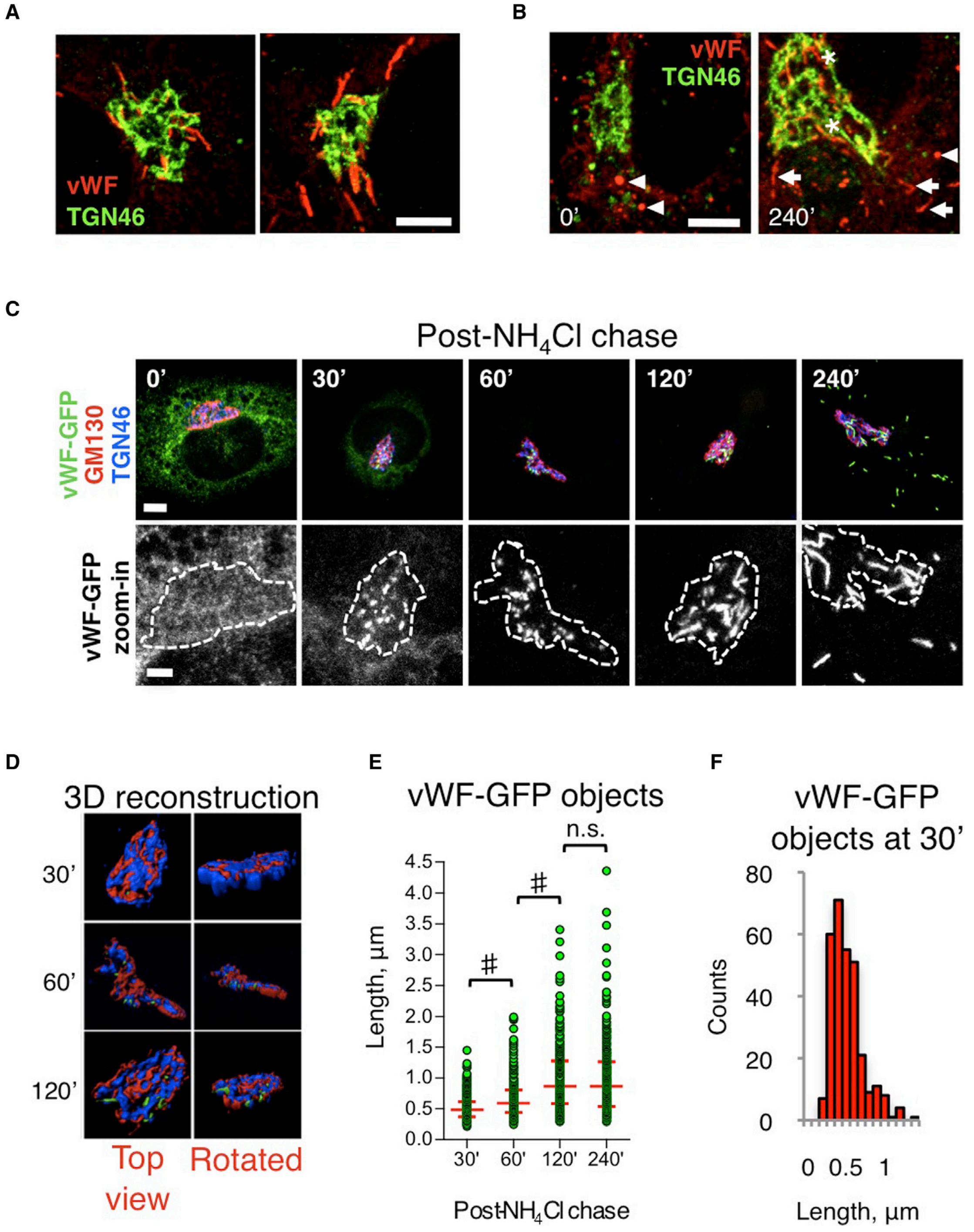


**Figure 3. WPB Size Is Acquired before Budding from Golgi**

(A) WPBs in the Golgi region of untreated HUVECs. Scale bar, 5 μm.

(B) WPB formation can be manipulated by $NH_4Cl$ incubation and washout. $NH_4Cl$-treated HUVECs (2 days) were subjected to washout and fixed immediately or after 240 min, and endogenous vWF was visualized. Neutralization of the lumen induces organelle rounding (arrowheads). After Golgi reacidification (240 min), organelles forming at the Golgi were visible (asterisks) and newly made WPBs (elongated shape, arrows) repopulated the cell periphery. Scale bar, 5 μm.

(C) The dynamics of WPB formation was analyzed using a vWF-GFP reporter. Dashed lines (zoom-in) outline the Golgi complex (GM130 and TGN46 staining). Scale bar, 5 μm; zoom, 2 μm.

(D) 3D reconstruction of the Golgi region of the cells shown in (C); forming WPBs are within the Golgi volume up to 120 min.

(E) Length of the vWF-GFP-labeled objects; medians and interquartile ranges are shown. n = 299, 243, 241, and 308 for 30', 60', 120', and 240', respectively. #$p < 10^{-10}$.

(F) Length frequency of vWF-GFP objects at 30 min.

See also Movie S1.

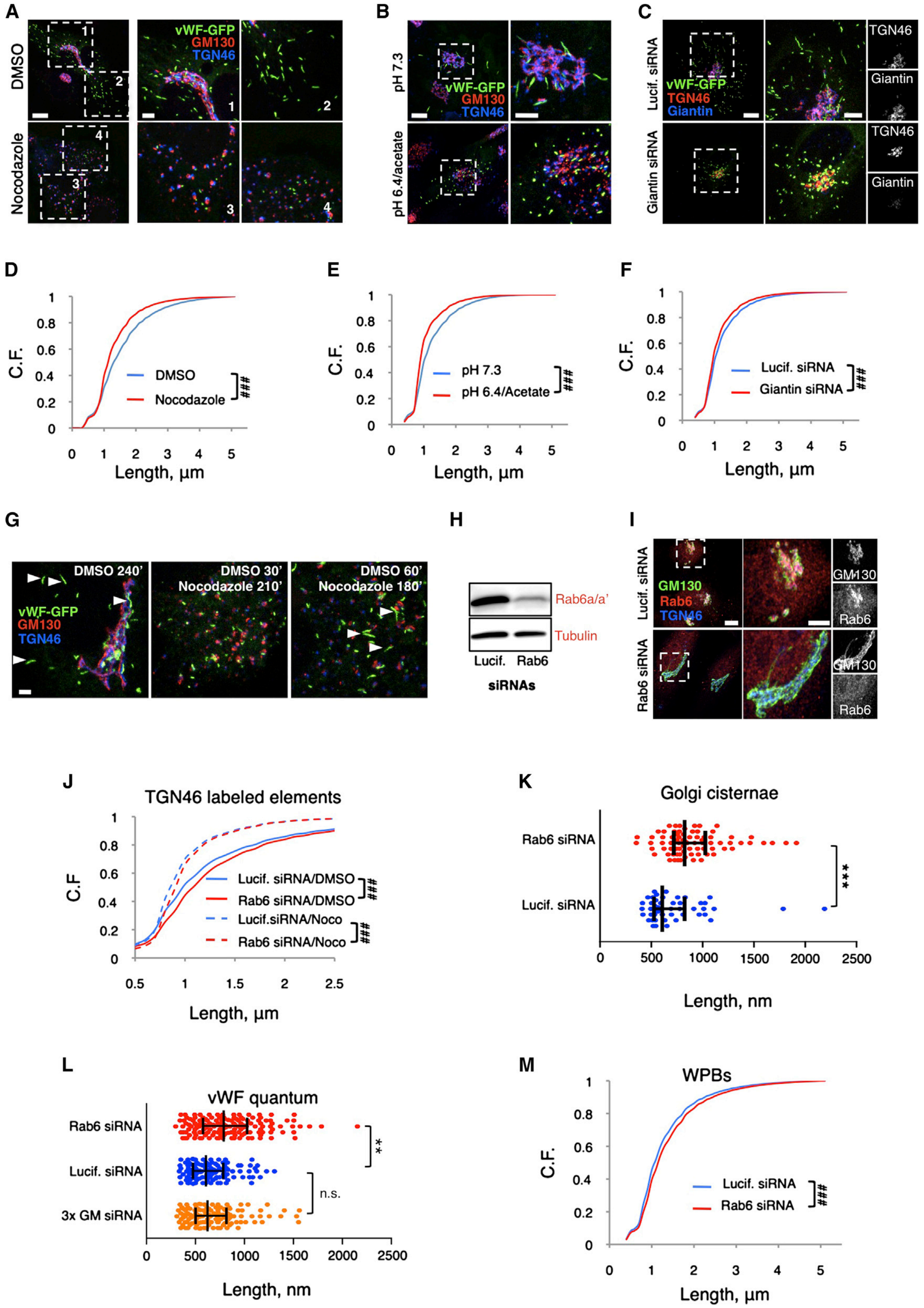


**Figure 4. Golgi Ministacks and Ribbon Architecture Determine the Size of WPBs**

(A–C) Effect of Golgi ribbon unlinking on newly made WPBs (vWF-GFP labeled). Unlinking was induced with nocodazole (A), by lowering the pH of the cytosol in the presence of acetate (B), or by Giantin siRNA (C). Scale bars, 10 µm; magnifications, 5 µm.

(legend continued on next page)

possibility that WPBs containing two or more quanta could be produced by post-Golgi homotypic fusion of one-quantum intermediates, in a process similar to the biogenesis of other secretory granules (Morvan and Tooze, 2008). vWF is structurally necessary for WPB production, and disrupting its processing and conformational rearrangements by neutralizing the acidic pH of the Golgi lumen prevents formation of WPBs (Wagner et al., 1986; Figure 3B). By coupling vWF-GFP expression to label newly formed WPBs with neutralization/reacidification of the Golgi lumen to control biogenesis, we analyzed the dynamics of WPB formation. At the Golgi, the initially small vWF-GFP-positive objects increased in size for up to 2 hr (Figures 3C–3E), when they resembled nascent Golgi-localized WPBs (refer to Figure 3A). However, between 2 and 4 hr, the time during which the organelles budded from the Golgi and populated the cell periphery, their size remained unchanged (Figures 3C and 3E). Of note, at 30 min, vWF-GFP object length peaked at ~500 nm (Figure 3F), similar to the vWF unit size identified by HTM and superresolution imaging and suggested by EM. These data indicate that the final length of WPBs is reached during formation at the Golgi and not by postbudding head-on homotypic fusion of short WPBs. This conclusion is also supported by live imaging (Movie S1).

## Dual Role of the Golgi in Determining WPB Size

What structural feature of the WPB biogenetic compartment, the Golgi apparatus, might be responsible for the generation of vWF quanta? The Golgi apparatus, as part of the endomembrane system, evolved before the divergence of nucleated cell lineages (Dacks and Field, 2007; Klute et al., 2011; Mowbrey and Dacks, 2009) and almost all eukaryotes share an identical organization of this organelle in the form of stacks of flattened membrane cisternae, which receive cargos on the entry side from the ER and, after processing, release them at the exit side for delivery to their final cellular destination. While in most eukaryotic phyla, the Golgi apparatus is a collection of monocisternal piles (ministacks) separated from one another and scattered throughout the cytoplasm (Donohoe et al., 2013; Kang and Staehelin, 2008; Mogelsvang et al., 2003; Witte et al., 2011), in vertebrates, through mechanisms involving microtubules, motors, tethers, and homotypic cisternal fusion events, the ministacks are linked together into a centralized Golgi apparatus. At the light-microscopy level, this appears as a lace-like perinuclear structure, known as the ribbon (Nakamura et al., 2012). Three-dimensional, ultrastructural imaging shows that within the ribbon, individual ministacks can still be recognized as piled cisternal "compact zones" separated from each other by either interruptions or tubular networks, known as "fenestrated zones" (Ladinsky et al., 1999; Weidman et al., 1993). The basic structure of the ministack is not the only common Golgi feature across eukaryotes; strikingly, its dimensions are conserved as well. Individual cisternae, and thus the ministacks, have sizes in the range of 500–1,000 nm (Donohoe et al., 2013; Kang and Staehelin, 2008; Ladinsky et al., 1999; Mogelsvang et al., 2003; Weidman et al., 1993; Witte et al., 2011). These features led us to hypothesize that the cisternal dimensions within ministacks might limit the size of the vWF quanta. At the exit side of the Golgi, the TGN, especially in HUVECs (Figure S5B), is mostly a continuous compartment within which adjacent quanta could be positioned for copackaging into nascent WPBs, explaining the generation of a population of organelles of variable size (see Figures 7A, S5A, and S5B). This mode of biogenesis predicts that unlinking the Golgi ribbon into separated ministacks should prevent copackaging of vWF quanta at the TGN, resulting in the production of short WPBs (see Figure 7B, "ribbon unlinking"). To test this prediction, we disassembled the ribbon by depolymerizing microtubules with nocodazole, a treatment that reverts the Golgi to the eukaryotic default organization with ministacks juxtaposed to ER exit sites (Cole et al., 1996; Donohoe et al., 2013; Kang and Staehelin, 2008; Mogelsvang et al., 2003; Witte et al., 2011). In nocodazole, only short ($\leq 2$ μm) new WPBs were formed (Figures 4A and S2A). The size of these "mini" WPBs seemed to be limited by that of the Golgi elements that generated them and similar to that of the quantum, suggesting that Golgi ministacks make "mini-WPBs," mostly containing a single vWF quantum, a conclusion supported by nanoscopy (Figures S2B–S2D). Ultrastructurally, these short organelles appeared morphologically normal (Figure S2I). Two alternative experimental approaches confirmed the effects of unlinking the Golgi ribbon on the size of newly made WPBs. In one, we lowered the cytosolic pH in the presence of acetate, which results in separated ministacks while leaving the microtubules unaffected (Figures 4B and S2E; Yoshida et al., 1999). In the other, we depleted three Golgi matrix proteins (GM130, GRASP55 [not shown], and Giantin) individually or together (Figures 4C, S2F, and S2G). The roles of these proteins in ribbon maintenance and ectopic ribbon formation were previously reported (Feinstein and Linstedt,

(D–F) HTM analysis of WPB length in cells treated as in (A)–(C). Endogenous vWF was stained to label the whole organelle population. Cumulative frequencies (C.F.) of the organelle number were plotted as function of organelle length. Number of WPBs analyzed: $\sim 10^4$–$10^6$. ###$p < 10^{-15}$.
(G) Cells nucleofected with vWF-GFP were incubated with $NH_4Cl$ as described in Figure 3. Golgi ribbon was unlinked with nocodazole at the indicated times after $NH_4Cl$ washout. Arrowheads indicate long WPBs; scale bar, 2 μm.
(H and I) siRNA-treatment targeting Rab6a/a' isoforms resulted in an ~85% reduction of their protein levels (H), in agreement with the fraction of cells showing loss of Rab6 staining at the Golgi (I). Scale bars, 10 μm; magnified 5 μm.
(J) Golgi-positive structures (TGN46 positive) in siRNA-treated cells incubated overnight with DMSO or nocodazole and analyzed by HTM. For each treatment, n = $\sim 10^5$; ###$p < 10^{-15}$.
(K) siRNA-treated cells were incubated with nocodazole to unlink ministacks in order to facilitate quantification. Electron micrographs of cells were inspected and cisternal lengths were measured. Medians (quartiles): luciferase, 612 (529, 827) nm; Rab6, 829 (725, 1,029) nm. n = 47 and 77 for luciferase and Rab6 siRNAs, respectively. ***$p < 10^{-3}$.
(L) vWF quantum size was measured in STORM images of siRNA-treated cells (3× GM indicates GM130, GRASP55, and Giantin triple knockdown). Medians and quartiles are shown. n = 153, 117, and 129 for luciferase, 3× GM, and Rab6 siRNAs, respectively. **$p < 10^{-2}$.
(M) HTM analysis of WPB length in siRNA-treated cells. For both treatments, n = $\sim 8 \times 10^5$; ###$p < 10^{-15}$.
See also Figures S2 and S3.

2008; Heuer et al., 2009; Koreishi et al., 2013; Puthenveedu et al., 2006). Irrespective of the ribbon-unlinking method used, the effect on the size of WPBs made during the experiments was sufficient to shift the length of the entire organelle population (Figures 4D–4F and S2H) while leaving the quantum size unchanged (Figure 4L). Finally, unlinking the ministacks before (30 min, Figures 3C and 3E) or soon after (60 min, Figures 3C and 3E) vWF-GFP objects began to grow in size during organelle formation showed that once WPB elongation (i.e., quanta copackaging) occurred, the ribbon was no longer required for the production of long organelles (Figure 4G).

Our model also predicts that if the cisternal dimensions limit those of the quantum, then changing the size of the cisternae should alter that of the quanta and, ultimately, that of WPBs (see Figure 7B, "cisternal lengthening"). Knockdown of Rab6a/a′ isoforms results in longer Golgi cisternae (Storrie et al., 2012), an effect that should be measurable as larger ribbons and, when they are unlinked, as larger ministacks. We confirmed both of these outcomes of Rab6a/a′ knockdown by HTM and by measuring cisternal length in electron micrographs (Figures 4H–4K, S3A, and S3B). vWF quantum size was increased in Rab6-siRNA treated cells (Figure 4L), an event reflected in a shift to higher values of WPB length clusters (Figure S3C). Finally, WPB size was increased in Rab6-depleted HUVECs, as was that of mini-WPBs upon ribbon unlinking (Figures 4M and S3D).

The effects of Golgi unlinking and cisternal lengthening on the sizes of the vWF quantum and WPBs support our biogenesis model (see Figure 7) and stress a requirement for the ribbon architecture of the Golgi to generate long WPBs from the quanta molded within the ministacks (Figure S5A).

## WPB Number and Size Depend on Cargo Availability

One model of organelle size control invokes regulation by component availability, that is, abundance of structural and enzymatic components will determine the size or number of organelles (Goehring and Hyman, 2012). In the case of WPBs, it is well established that the cargo protein vWF is the driver for organelle formation (Wagner et al., 1991). We observed that the siRNA-mediated reduction of vWF protein levels resulted in a decrement not only of the number of WPBs, as might be expected, but also of the length of the residual organelles (Figure 5C). We tested the impact of vWF levels on the length of WPBs by titrating the vWF-targeting siRNA. The consequent gradual reduction in vWF cellular content correlated with a gradual decrease in the number of WPBs and, importantly, a shortening of WPB length (Figures 5D–5F). In contrast to the number and length of WPBs, the quantum size, as measured by the distance between length clusters, remained constant upon progressive reduction of vWF cell content (Figure 5G; Table S2). Control of the quantum size, therefore, seems to be independent of vWF levels and primarily set by cisternal dimensions (see Figure 7C).

## Functional Effects of WPB Size

Long WPBs are a minority but contain a considerable fraction of vWF cargo (Figure S1D), suggesting that this subpopulation of organelles might be especially important. We assayed the secretory behavior of cells enriched in short WPBs. To prompt the formation of mini-WPBs, we performed the two manipulations that had been shown to control organelle size: low levels of vWF expression and Golgi ribbon unlinking. We used nocodazole to unlink the ribbon, but before assaying secretion, we subjected the cells to washout and a short chase to allow reformation of microtubules (Figure S4A). Although working through different mechanisms, both of these size-reducing treatments had the same effect: vWF secretion was enhanced in the absence of secretagogue and reduced in its presence when compared with control cells (Figure 6A). Since ~80% of synthesized vWF is stored in WPBs and its basal secretion occurs from WPBs (Giblin et al., 2008), the secretory phenotype observed suggested that short WPBs are less responsive to secretory agonists and are the main contributors to basal secretion of vWF. WPB size might therefore represent an important factor in the prothrombotic function of endothelial cells.

Although it may seem obvious that the size of WPBs is reflected in their hemostatic capability, structural considerations highlight that this relationship in fact cannot be predicted. vWF strings provide the adhesion platforms for circulating platelets in primary hemostasis (De Ceunynck et al., 2013; Dong et al., 2002). Strings are formed from secreted multimerised vWF, and multimer size affects string length (Nightingale et al., 2009). During biosynthesis, an unknown fraction of the total vWF is tubulated, a conformational arrangement that seems to be required for multimer formation (Huang et al., 2008; Zhou et al., 2011; summarized in Figure 1).

The largest multimers found in WPBs (Figure 1E) with ~50 subunits (i.e., ~25 dimers) in the coiled configuration would span only ~63 nm of a tubule length (4.2 dimers per turn with a 11 nm pitch; see Figure 1; Huang et al., 2008). Therefore, the structural data suggest that tubules are for the most part composed of more than one multimer. Similarly, at exocytosis, a vWF 50-mer would extend for ~3 μm (~60 nm per subunit; see Figure 1D; Fowler et al., 1985), a size that alone cannot account for that of the vWF platelet-catching strings, some of which extend up to hundreds of micrometers (De Ceunynck et al., 2013). To establish whether organelle size does indeed have an effect on WPB hemostatic function, we experimentally addressed the issue.

Cell-free mini-WPBs produced shorter vWF filaments than control WPBs (Figures 6B, 6C, and S4D), confirming that experimentally induced mini-WPBs are structurally normal, since they store vWF that can make multimers and filaments (Figures 6B and S4C), and strongly suggesting a link between organelle length and hemostatic capability.

To test this more directly, we assayed vWF string formation and platelet capture by HUVECs under flow. In these experiments, mini-WPBs in HUVECs were obtained by nocodazole treatment, which had only a minimal effect on the number of WPBs per cell compared with vWF knockdown (Figures 5E and S4B). No platelet-decorated vWF strings were formed in unstimulated control or mini-WPB-bearing cells (Figure S4E). However, in the presence of histamine, platelet-decorated vWF string generation was strongly reduced from cells with short WPBs, and those fewer strings were also shorter (Figures 6D, 6E, and S4F). Of note, the extent of string number reduction could not be accounted for by the modest reduction in WPB numbers in nocodazole-treated cells (Figures S4B and S4F), suggesting that string formation might be cooperative in nature and thus subject to threshold effects. These data show that

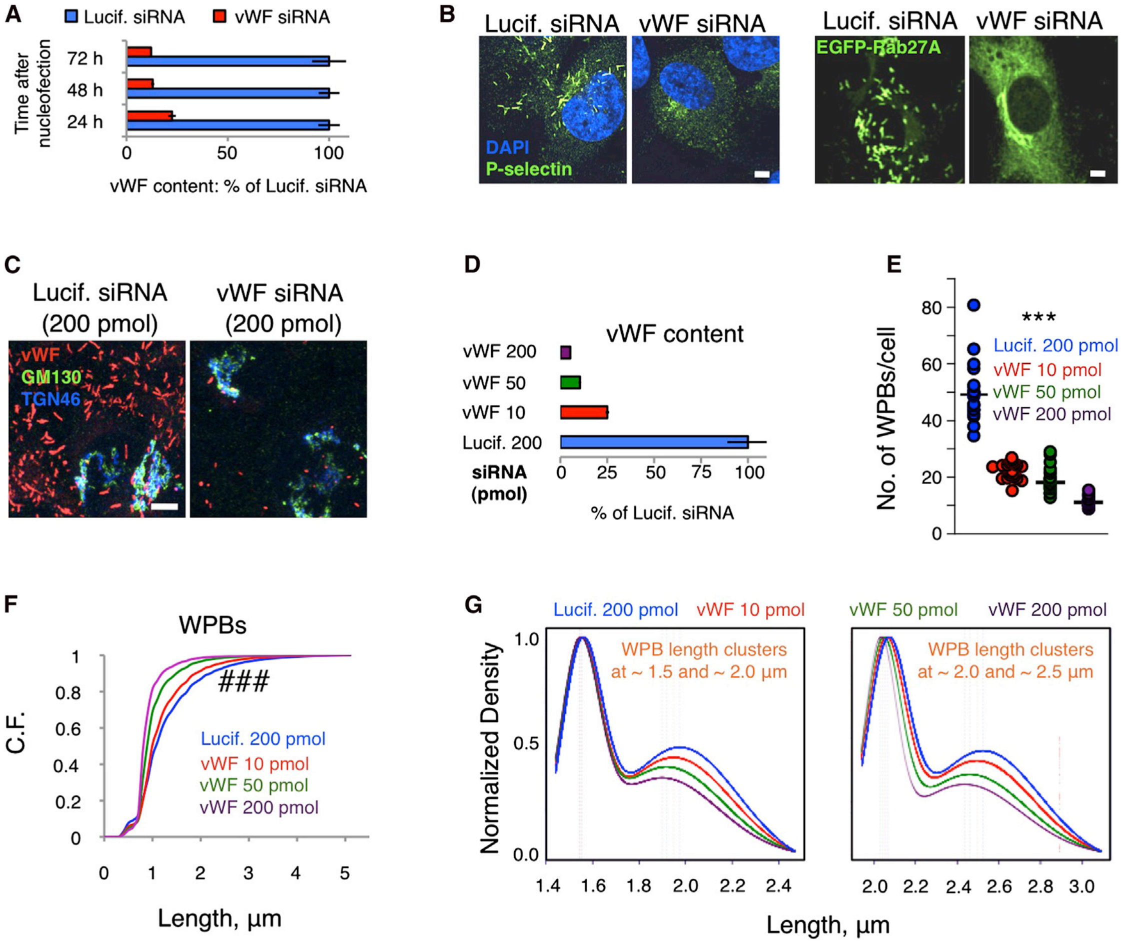


**Figure 5. vWF Levels Control WPB Number and Size**

(A) Time course of siRNA-mediated vWF depletion (at 200 pmol siRNA/nucleofection reaction). vWF content was normalized to protein in cell lysates; mean ± SD (n = 3).

(B and C) vWF depletion causes mislocalization of the WPB markers P-selectin and Rab27A (B) and a reduction in size of the residual organelles (C). Scale bars, 5 µm.

(D) vWF cell content was modulated by titrating the amount of vWF-targeting siRNA delivered; mean ± SD.

(E) HTM analysis of the number of WPBs per cell following vWF-targeting siRNA titration; median values are shown (n = 24 per treatment, with each observation from a separate well of 96-well plates); ***$p < 10^{-3}$ for all the pairwise comparisons.

(F) Decrease in vWF cell content results in shortening of WPBs. Per treatment, n = $2.3 \times 10^5$ to $8 \times 10^5$; ###$p < 10^{-15}$ for all pairwise comparisons.

(G) Kernel densities for each data set in the indicated length ranges were calculated as in Figure 2B and normalized to the length cluster of highest density. The distance between the length clusters was unaffected (Table S2), indicating that vWF quantum size remains constant over an ~20-fold change in cargo abundance (see D, Lucif. 200 pmol versus vWF 200 pmol).

the subpopulation of long WPBs is disproportionately important for the production of vWF strings efficient in platelet recruitment.

In response to inflammation, circulating leukocytes are recruited to the affected tissues. Signaling to endothelial cells prompts exocytosis of WPBs, whose membrane cargo, P-selectin, becomes exposed in the vessel lumen, where it functions by binding circulating leukocytes. By decelerating the leukocytes, this interaction allows them to roll along the vessel wall and then firmly adhere to it, as required for their extravasation into the inflamed tissue (Mayadas et al., 1993). We tested whether the size of WPBs had any impact on this function, by measuring the rolling of THP-1 monocyte-like cells on HUVECs under conditions in which leukocyte-endothelial cell interactions depend exclusively on P-selectin (Doyle et al., 2011). Histamine stimulation induced this interaction in control cells and their mini-WPB-enriched counterparts to the same extent (Figures 5F and S4G). Thus, the proinflammatory function of WPBs is not affected by their size.

## DISCUSSION

We report a previously unappreciated role for the Golgi apparatus in controlling the size of a secretory carrier. This control requires cooperation between the two structural levels of this fundamental organelle: its functional unit, the ministack, and its compound architecture, the ribbon. Our data support

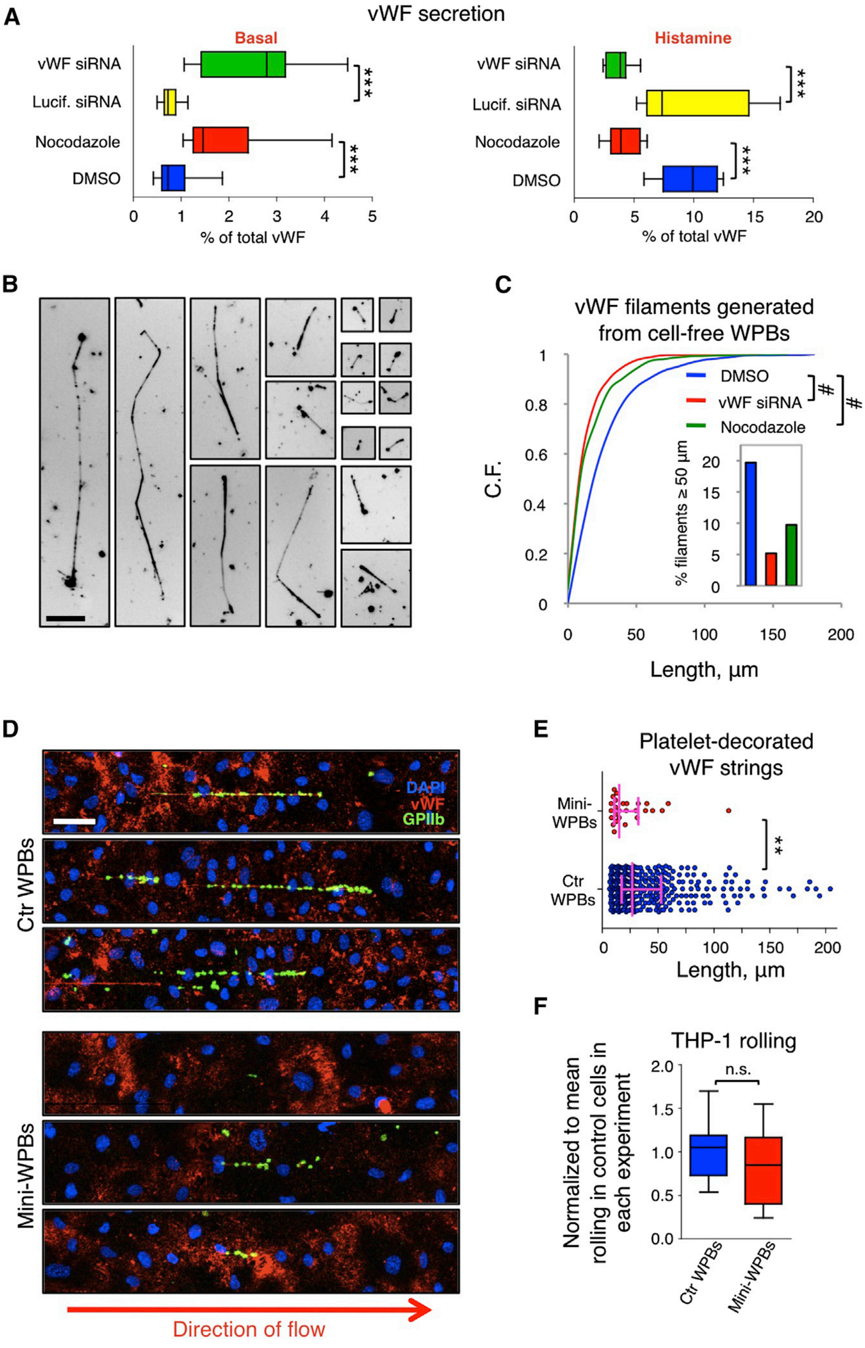


**Figure 6. WPB Size and Endothelial Function**

(A) vWF release in the absence or presence of histamine measured from control (200 pmol luciferase siRNA/reaction or DMSO) and mini-WPB enriched HUVEC cells (200 pmol vWF siRNA/reaction or nocodazole). Box plots of 9–18 measurements per treatment from 3–4 experiments are shown. ***$p < 10^{-3}$.

(B) Cell-free WPBs were diluted and permeabilized to obtain well-separated vWF filaments. Representative images from control, VWF siRNA-, and nocodazole-treated samples are shown. Scale bar, 25 μm.

(C) Cumulative frequency of vWF filament number as a function of filament length generated by cell-free WPBs obtained from HUVECs treated as indicated. DMSO, n = 858; vWF siRNA, n = 851; nocodazole, n = 885. #$p < 10^{-10}$. Inset: percentage of long filaments in each treatment.

(D) Formation of platelet-decorated vWF strings under flow following HUVEC stimulation with histamine. Platelets were identified by CD41/GPIIb labeling.

(E) Length of platelet-decorated vWF strings; median and interquartile ranges are shown. Control, n = 366; mini-WPBs, n = 27 from 2 separate experiments. **$p < 10^{-2}$.

(F) Rolling of THP-1 monocyte-like cells on HUVEC monolayers following histamine stimulation; box plot of 12 measurements from 4 separate experiments.

See also Figure S4.

a "two-tier" model of control of organelle size during biogenesis: within the ministacks, cisternal dimensions limit those of the forming vWF quanta, which in the continuous lumen provided by the TGN of linked ministacks can then be copackaged into nascent WPBs of sizes determined by the number of quanta they contain (Figures 7 and S5).

The Golgi apparatus is almost universally present in eukaryotes as scattered ministacks (Donohoe et al., 2013; Kang and Staehelin, 2008; Kondylis et al., 2007; Mogelsvang et al., 2003; Witte et al., 2011) and the mammalian Golgi ribbon can be reverted to this "default" organization without major defects in secretion (Cole et al., 1996). Why vertebrate cells need a ribbon is not fully understood, though experimental evidence suggests that this peculiar architecture might be required for the homogeneous distribution of processing enzymes, polarized secretion, cell migration, and function as a mitotic checkpoint (Miller et al., 2009; Puthenveedu et al., 2006; Rabouille and Kondylis, 2007; Yadav et al., 2009). Now we find that endothelial cells exploit the presence of a ribbon to produce a subset of long WPBs that are required for recruitment of platelets, a newly appreciated function for this Golgi superstructure.

Control of size endows WPBs with plasticity by allowing the uncoupling of their hemostatic and proinflammatory activities. Long WPBs are necessary for efficient platelet recruitment, a key event in primary hemostasis, but are dispensable for leukocyte rolling on endothelia, a process mediated by P-selectin and necessary for an adequate inflammatory response (Mayadas et al., 1993). P-selectin, the principal leukocyte receptor in the initial inflammatory response, is sorted to the WPB membrane during formation of these organelles by interacting with vWF (Bonfanti et al., 1989; Michaux et al., 2006b). Storage of P-selectin in WPBs is required for its function, since endothelial cells of

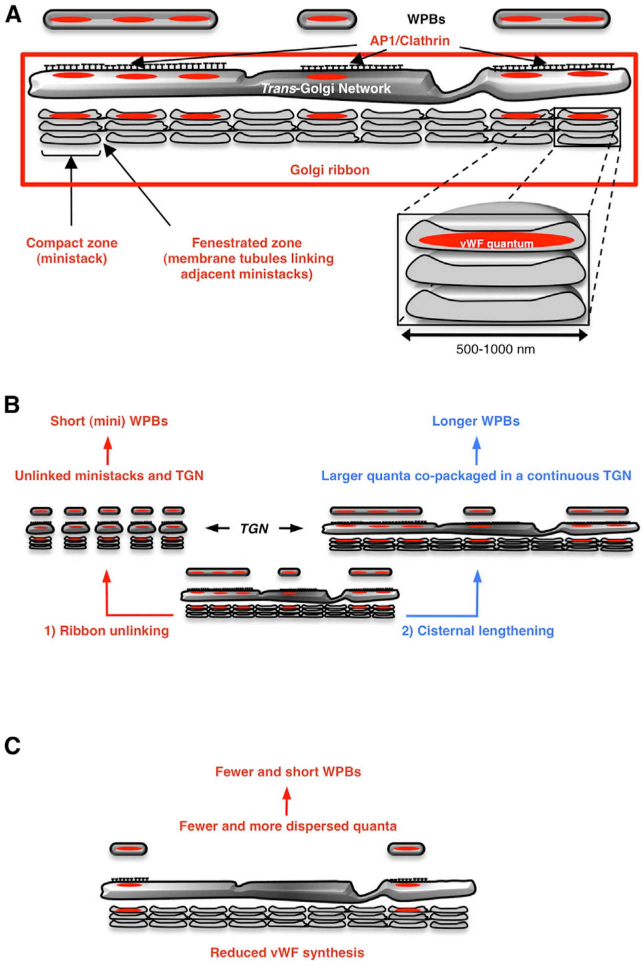


**Figure 7. Structural Units and Compound Architecture of the Golgi Cooperate to Generate WPB Size**

(A) In vertebrates, the Golgi's structural units, the ministacks, are brought into close proximity by motors and microtubules and fuse to their neighbors via tubular connections between homologous cisternae (fenestrated zones), generating a higher-order architecture, the ribbon (boxed in red). The cisternal dimensions limit the size of the vWF quanta, but in the continuous lumen of the TGN, adjacent quanta can be copackaged together into forming organelles. This process requires the adaptor complex AP1 and clathrin, which likely act as a scaffold (Lui-Roberts et al., 2005). The size of WPBs generated in this process depends on the number of copackaged quanta.

(B) Unlinking the ribbon into separated ministacks has no effect on quantum size, but partitions the TGN and prevents multiple quanta copackaging. This results in the formation of short WPBs (pathway 1). Increasing the dimension of Golgi cisternae allows formation of bigger vWF quanta, whose copackaging produces longer WPBs (pathway 2).

(C) Reduced synthesis of vWF results in fewer and more dispersed quanta, which are packaged in fewer and shorter WPBs.

See also Figure S5.

vWF-deficient mice, which are devoid of WPBs but maintain levels of P-selectin similar to those in wild-type cells, display impaired leukocyte rolling and extravasation (Denis et al., 2001). Our finding that the production of short WPBs does not affect leukocyte recruitment in vitro indicates that vWF-dependent P-selectin sorting to the smaller organelles and thus the function of those organelles in inflammation are unaffected.

The discovery of WPB plasticity suggests a potential capability for vascular beds to regulate, spatially and/or temporally, their prothrombotic propensity by controlling the size of the WPBs they produce. Locally and over time, an endothelial district could modulate its own function by two mechanisms: transcriptional regulation of vWF cellular levels and changes in the Golgi architecture. Signaling networks modulate the architecture of the Golgi apparatus (Chia et al., 2012); therefore, pathways driving a lower or higher degree of ministack linking into a ribbon are bound to result in the production of short or long WPB cohorts, respectively, with consequences for their hemostatic activity. It has been reported that endothelial expression of the transcription factor KFL2 induces formation of short WPBs (van Agtmaal et al., 2012). Our findings suggest the intriguing possibility that the antithrombotic status induced by KLF2 (Lin et al., 2005) might be mediated at least in part by its effects on WPB size. Since expression of KLF2 does not reduce but actually upregulates vWF levels (Dekker et al., 2006; van Agtmaal et al., 2012), its effects on WPB size might be mediated through regulation of the Golgi ribbon, a hypothesis worth future study.

Size control of cellular organelles seems to be required in many developmental and homeostatic processes (Kirk et al., 2010; Levy and Heald, 2010, 2012; Rafelski et al., 2012; Sardiello et al., 2009; Schuck et al., 2009; Settembre et al., 2012). One mode of size control invokes component-mediated regulation, whereby levels of structural components, such as proteins and lipids, determine the organelle volume/size (Chan and Marshall, 2010; Goehring and Hyman, 2012). For single-copy organelles, component upregulation can thus lead to expansion, as in the case of the ER (Kirk et al., 2010; Schuck et al., 2009), whereas in the case of multicopy organelles, it can modulate the combined compartment volume by adjusting the number, as lysosomes do (Sardiello et al., 2009).

WPBs conform to the component-mediated size-control model by combining the modalities for single- and multicopy organelles: the expression levels of vWF, the cargo that is structurally necessary for organelle formation, impact not only the number but also the length of these secretory granules. This peculiarity might be explained by the existence of the vWF quantum. The Golgi will organize lower levels of vWF into fewer, more dispersed quanta that are not copackaged, thus generating both fewer and smaller WPBs (Figure 7C).

With regard to the supramolecular nature of the quantum, further investigations are needed to clarify its relationship to the other vWF structure observed within WPBs, the tubule. It is generally assumed (but remains to be proved) that vWF sorted into WPBs is present only in its tubulated form. Electron micrographs of WPB transverse sections often show amorphous content covering a large area interspersed with tubules, a morphological arrangement that has sparked speculation about the presence of an intertubule scaffold (Valentijn et al., 2008). Since vWF is the most abundant protein in WPBs (Ewenstein et al., 1987), it is reasonable to conclude that much of this cargo is not tubulated and might therefore provide the suggested "scaffold." STORM images reflect antibody labeling and may detect vWF pools that overlap with the fraction structured into tubules only partially or not at all. Irrespective of its supramolecular nature, the quantum reflects either different vWF

concentrations (as suggested in part by the inhomogeneous electron density in our EM data) and/or antibody accessibility. We also note that vWF tubules are almost invariably straight, an indication that they are stiff structures. Were all tubules to extend for the entire length of an organelle, it would be difficult to reconcile this rigidity with live imaging data showing that WPBs do bend, a clear sign of structural flexibility (Zenner et al., 2007). A possibility is therefore that the quantal arrangement of vWF may confer or reflect structural flexibility in long organelles.

The distinctive cigar-like shape of WPBs is of physiological significance because it mirrors the lumenal arrangement of vWF cargo in tubules. Perturbing the structure of the tubules results in round WPBs that respond normally to secretagogues but release tangled vWF strings that bind fewer platelets (Michaux et al., 2006a). Here, we report how size is also critical to WPB function.

## EXPERIMENTAL PROCEDURES

### Cells, Culture Conditions, and Nucleofection

HUVECs from multiple donors were obtained from TCS Cellworks or Lonza. Cells were maintained as previously described (Zenner et al., 2007) and used within passage 4. Plasmids and siRNAs were introduced by nucleofection (Lonza) using a constant number of cells ($2 \times 10^6$) per reaction. See Supplemental Experimental Procedures for further details.

### Confocal Microscopy

Cells were fixed with 4% formaldehyde in PBS. Unless otherwise specified, samples were permeabilized and then incubated with primary antibodies followed by secondary antibodies conjugated to Alexa Fluor dyes (Molecular Probes, Life Technologies) or Cy5 (Jackson ImmunoResearch Laboratories). Mounted samples (ProLong Gold antifade reagent, Life Technologies) were imaged with a 63× oil immersion objective (NA 1.3) on a Leica Microsystems TCS SPE confocal system. Maximum intensity projections of image stacks (0.5 μm z step) are shown unless stated otherwise. See Supplemental Experimental Procedures for details.

### High-Throughput Confocal Microscopy

Cells cultured in 96-well plates were fixed and immunostained to label WPBs. Hoechst 33342 (Life Technologies) was used to counterstain nuclei. An Opera High Content Screening System (Perkin Elmer) was used for image acquisition of 5 to 25 fields of view per well (depending on the experiment) using a 40× air objective (NA 0.6). Image processing and parameter measurements from HTM are detailed in the Supplemental Experimental Procedures.

### Stochastic Optical Reconstruction Microscopy

Cells were immunostained as described for confocal microscopy. Images were acquired using a modified Olympus IX71 inverted objective-based total internal reflection (TIRF) microscope. The detailed workflow is available in Supplemental Experimental Procedures.

### Electron Microscopy

Chemically fixed or high-pressure frozen and free-substituted (HPF/FS) samples were processed as previously described (Michaux et al., 2006a; Zenner et al., 2007). For whole mounts of cell-free WPBs, samples were adsorbed for 10 min on formvar, carbon-coated, and glow-discharged copper grids (Agar Scientific) before fixation in 2% paraformaldehyde/1.5% glutaraldehyde in 0.1 M sodium cacodylate for 1 hr. After osmication and serial dehydration to 100% dry ethanol, the grids were critical-point dried using a Leica EM-CPD300 (Leica Microsystems). All samples were imaged with a Morada camera (OlympusSIS) in a Tecnai20 (FEI). iTEM software (Olympus SIS) was used to measure cisternal length of ministacks in nocodazole-treated luciferase- and Rab6a/a′-siRNA-treated HUVECs.

### Platelet-Decorated vWF Strings and Leukocyte Rolling

Recruitment assays of platelets or THP-1 cells under flow by resting or secretagogue stimulated endothelial monolayers were carried out essentially as previously described (Doyle et al., 2011; Michaux et al., 2006a). See Supplemental Experimental Procedures for details.

### Statistical Analysis

Nonparametric, two-tailed, two-independent-sample Wilcoxon rank-sum test was used. Data sets were analyzed using R (http://www.r-project.org/; see Supplemental Experimental Procedures; p values calculated up $10^{-15}$) or SOCR (Statistical Online Computational Resource, at University of California, Los Angeles; http://www.socr.ucla.edu/SOCR.html) for calculation of p values up to $10^{-10}$.

## SUPPLEMENTAL INFORMATION

Supplemental Information includes Supplemental Experimental Procedures, five figures, two tables, and one movie and can be found with this article online at http://dx.doi.org/10.1016/j.devcel.2014.03.021.

## ACKNOWLEDGMENTS

The authors thank Martin Raff, Louise Cramer, Brian Burke, Gustavo Pigino, Steven Moss, Michael Marks, and members of the D.F.C. lab for critical readings of the manuscript and are grateful to Andrew Vaughan and Lauren Mclaughlin for technical assistance. The Medical Research Council UK funded the work at the Laboratory for Molecular Cell Biology. The European Union Seventh Framework Programme (FP7/2007-2013, grant PIRG08-GA-2010-276811) funded J.K.-V. The Chemical and Biological Metrology Programme of the UK's National Measurement Office funded D.J.M. and A.E.K.

Received: July 15, 2013
Revised: February 3, 2014
Accepted: March 27, 2014
Published: May 1, 2014

## REFERENCES

Bonfanti, R., Furie, B.C., Furie, B., and Wagner, D.D. (1989). PADGEM (GMP140) is a component of Weibel-Palade bodies of human endothelial cells. Blood *73*, 1109–1112.

Chan, Y.H., and Marshall, W.F. (2010). Scaling properties of cell and organelle size. Organogenesis *6*, 88–96.

Chia, J., Goh, G., Racine, V., Ng, S., Kumar, P., and Bard, F. (2012). RNAi screening reveals a large signaling network controlling the Golgi apparatus in human cells. Mol. Syst. Biol. *8*, 629.

Cole, N.B., Sciaky, N., Marotta, A., Song, J., and Lippincott-Schwartz, J. (1996). Golgi dispersal during microtubule disruption: regeneration of Golgi stacks at peripheral endoplasmic reticulum exit sites. Mol. Biol. Cell *7*, 631–650.

Dacks, J.B., and Field, M.C. (2007). Evolution of the eukaryotic membrane-trafficking system: origin, tempo and mode. J. Cell Sci. *120*, 2977–2985.

De Ceunynck, K., De Meyer, S.F., and Vanhoorelbeke, K. (2013). Unwinding the von Willebrand factor strings puzzle. Blood *121*, 270–277.

Dekker, R.J., Boon, R.A., Rondaij, M.G., Kragt, A., Volger, O.L., Elderkamp, Y.W., Meijers, J.C., Voorberg, J., Pannekoek, H., and Horrevoets, A.J. (2006). KLF2 provokes a gene expression pattern that establishes functional quiescent differentiation of the endothelium. Blood *107*, 4354–4363.

Denis, C., Methia, N., Frenette, P.S., Rayburn, H., Ullman-Culleré, M., Hynes, R.O., and Wagner, D.D. (1998). A mouse model of severe von Willebrand disease: defects in hemostasis and thrombosis. Proc. Natl. Acad. Sci. USA *95*, 9524–9529.

Denis, C.V., André, P., Saffaripour, S., and Wagner, D.D. (2001). Defect in regulated secretion of P-selectin affects leukocyte recruitment in

von Willebrand factor-deficient mice. Proc. Natl. Acad. Sci. USA *98*, 4072–4077.

Dong, J.F., Moake, J.L., Nolasco, L., Bernardo, A., Arceneaux, W., Shrimpton, C.N., Schade, A.J., McIntire, L.V., Fujikawa, K., and López, J.A. (2002). ADAMTS-13 rapidly cleaves newly secreted ultralarge von Willebrand factor multimers on the endothelial surface under flowing conditions. Blood *100*, 4033–4039.

Donohoe, B.S., Kang, B.H., Gerl, M.J., Gergely, Z.R., McMichael, C.M., Bednarek, S.Y., and Staehelin, L.A. (2013). Cis-Golgi cisternal assembly and biosynthetic activation occur sequentially in plants and algae. Traffic *14*, 551–567.

Doyle, E.L., Ridger, V., Ferraro, F., Turmaine, M., Saftig, P., and Cutler, D.F. (2011). CD63 is an essential cofactor to leukocyte recruitment by endothelial P-selectin. Blood *118*, 4265–4273.

Ewenstein, B.M., Warhol, M.J., Handin, R.I., and Pober, J.S. (1987). Composition of the von Willebrand factor storage organelle (Weibel-Palade body) isolated from cultured human umbilical vein endothelial cells. J. Cell Biol. *104*, 1423–1433.

Feinstein, T.N., and Linstedt, A.D. (2008). GRASP55 regulates Golgi ribbon formation. Mol. Biol. Cell *19*, 2696–2707.

Fölling, J., Bossi, M., Bock, H., Medda, R., Wurm, C.A., Hein, B., Jakobs, S., Eggeling, C., and Hell, S.W. (2008). Fluorescence nanoscopy by ground-state depletion and single-molecule return. Nat. Methods *5*, 943–945.

Fowler, W.E., Fretto, L.J., Hamilton, K.K., Erickson, H.P., and McKee, P.A. (1985). Substructure of human von Willebrand factor. J. Clin. Invest. *76*, 1491–1500.

Giblin, J.P., Hewlett, L.J., and Hannah, M.J. (2008). Basal secretion of von Willebrand factor from human endothelial cells. Blood *112*, 957–964.

Goehring, N.W., and Hyman, A.A. (2012). Organelle growth control through limiting pools of cytoplasmic components. Curr. Biol. *22*, R330–339.

Heuer, D., Rejman Lipinski, A., Machuy, N., Karlas, A., Wehrens, A., Siedler, F., Brinkmann, V., and Meyer, T.F. (2009). Chlamydia causes fragmentation of the Golgi compartment to ensure reproduction. Nature *457*, 731–735.

Huang, R.H., Wang, Y., Roth, R., Yu, X., Purvis, A.R., Heuser, J.E., Egelman, E.H., and Sadler, J.E. (2008). Assembly of Weibel-Palade body-like tubules from N-terminal domains of von Willebrand factor. Proc. Natl. Acad. Sci. USA *105*, 482–487.

Kang, B.H., and Staehelin, L.A. (2008). ER-to-Golgi transport by COPII vesicles in Arabidopsis involves a ribosome-excluding scaffold that is transferred with the vesicles to the Golgi matrix. Protoplasma *234*, 51–64.

Kirk, S.J., Cliff, J.M., Thomas, J.A., and Ward, T.H. (2010). Biogenesis of secretory organelles during B cell differentiation. J. Leukoc. Biol. *87*, 245–255.

Klute, M.J., Melançon, P., and Dacks, J.B. (2011). Evolution and diversity of the Golgi. Cold Spring Harb. Perspect. Biol. *3*, a007849.

Kondylis, V., van Nispen tot Pannerden, H.E., Herpers, B., Friggi-Grelin, F., and Rabouille, C. (2007). The golgi comprises a paired stack that is separated at G2 by modulation of the actin cytoskeleton through Abi and Scar/WAVE. Dev. Cell *12*, 901–915.

Koreishi, M., Gniadek, T.J., Yu, S., Masuda, J., Honjo, Y., and Satoh, A. (2013). The golgin tether giantin regulates the secretory pathway by controlling stack organization within Golgi apparatus. PLoS ONE *8*, e59821.

Ladinsky, M.S., Mastronarde, D.N., McIntosh, J.R., Howell, K.E., and Staehelin, L.A. (1999). Golgi structure in three dimensions: functional insights from the normal rat kidney cell. J. Cell Biol. *144*, 1135–1149.

Levy, D.L., and Heald, R. (2010). Nuclear size is regulated by importin $\alpha$ and Ntf2 in Xenopus. Cell *143*, 288–298.

Levy, D.L., and Heald, R. (2012). Mechanisms of intracellular scaling. Annu. Rev. Cell Dev. Biol. *28*, 113–135.

Lin, Z., Kumar, A., SenBanerjee, S., Staniszewski, K., Parmar, K., Vaughan, D.E., Gimbrone, M.A., Jr., Balasubramanian, V., García-Cardeña, G., and Jain, M.K. (2005). Kruppel-like factor 2 (KLF2) regulates endothelial thrombotic function. Circ. Res. *96*, e48–e57.

Lui-Roberts, W.W., Collinson, L.M., Hewlett, L.J., Michaux, G., and Cutler, D.F. (2005). An AP-1/clathrin coat plays a novel and essential role in forming the Weibel-Palade bodies of endothelial cells. J. Cell Biol. *170*, 627–636.

Mayadas, T.N., and Wagner, D.D. (1989). In vitro multimerization of von Willebrand factor is triggered by low pH. Importance of the propolypeptide and free sulfhydryls. J. Biol. Chem. *264*, 13497–13503.

Mayadas, T.N., Johnson, R.C., Rayburn, H., Hynes, R.O., and Wagner, D.D. (1993). Leukocyte rolling and extravasation are severely compromised in P selectin-deficient mice. Cell *74*, 541–554.

Metcalf, D.J., Nightingale, T.D., Zenner, H.L., Lui-Roberts, W.W., and Cutler, D.F. (2008). Formation and function of Weibel-Palade bodies. J. Cell Sci. *121*, 19–27.

Methia, N., André, P., Denis, C.V., Economopoulos, M., and Wagner, D.D. (2001). Localized reduction of atherosclerosis in von Willebrand factor-deficient mice. Blood *98*, 1424–1428.

Michaux, G., Abbitt, K.B., Collinson, L.M., Haberichter, S.L., Norman, K.E., and Cutler, D.F. (2006a). The physiological function of von Willebrand's factor depends on its tubular storage in endothelial Weibel-Palade bodies. Dev. Cell *10*, 223–232.

Michaux, G., Pullen, T.J., Haberichter, S.L., and Cutler, D.F. (2006b). P-selectin binds to the D'-D3 domains of von Willebrand factor in Weibel-Palade bodies. Blood *107*, 3922–3924.

Miller, P.M., Folkmann, A.W., Maia, A.R., Efimova, N., Efimov, A., and Kaverina, I. (2009). Golgi-derived CLASP-dependent microtubules control Golgi organization and polarized trafficking in motile cells. Nat. Cell Biol. *11*, 1069–1080.

Mogelsvang, S., Gomez-Ospina, N., Soderholm, J., Glick, B.S., and Staehelin, L.A. (2003). Tomographic evidence for continuous turnover of Golgi cisternae in Pichia pastoris. Mol. Biol. Cell *14*, 2277–2291.

Morvan, J., and Tooze, S.A. (2008). Discovery and progress in our understanding of the regulated secretory pathway in neuroendocrine cells. Histochem. Cell Biol. *129*, 243–252.

Mowbrey, K., and Dacks, J.B. (2009). Evolution and diversity of the Golgi body. FEBS Lett. *583*, 3738–3745.

Nakamura, N., Wei, J.H., and Seemann, J. (2012). Modular organization of the mammalian Golgi apparatus. Curr. Opin. Cell Biol. *24*, 467–474.

Nightingale, T.D., Pattni, K., Hume, A.N., Seabra, M.C., and Cutler, D.F. (2009). Rab27a and MyRIP regulate the amount and multimeric state of VWF released from endothelial cells. Blood *113*, 5010–5018.

Petri, B., Broermann, A., Li, H., Khandoga, A.G., Zarbock, A., Krombach, F., Goerge, T., Schneider, S.W., Jones, C., Nieswandt, B., et al. (2010). von Willebrand factor promotes leukocyte extravasation. Blood *116*, 4712–4719.

Puthenveedu, M.A., Bachert, C., Puri, S., Lanni, F., and Linstedt, A.D. (2006). GM130 and GRASP65-dependent lateral cisternal fusion allows uniform Golgi-enzyme distribution. Nat. Cell Biol. *8*, 238–248.

Rabouille, C., and Kondylis, V. (2007). Golgi ribbon unlinking: an organelle-based G2/M checkpoint. Cell Cycle *6*, 2723–2729.

Rafelski, S.M., Viana, M.P., Zhang, Y., Chan, Y.H., Thorn, K.S., Yam, P., Fung, J.C., Li, H., Costa Lda, F., and Marshall, W.F. (2012). Mitochondrial network size scaling in budding yeast. Science *338*, 822–824.

Rust, M.J., Bates, M., and Zhuang, X. (2006). Sub-diffraction-limit imaging by stochastic optical reconstruction microscopy (STORM). Nat. Methods *3*, 793–795.

Sadler, J.E. (1998). Biochemistry and genetics of von Willebrand factor. Annu. Rev. Biochem. *67*, 395–424.

Sardiello, M., Palmieri, M., di Ronza, A., Medina, D.L., Valenza, M., Gennarino, V.A., Di Malta, C., Donaudy, F., Embrione, V., Polishchuk, R.S., et al. (2009). A gene network regulating lysosomal biogenesis and function. Science *325*, 473–477.

Schuck, S., Prinz, W.A., Thorn, K.S., Voss, C., and Walter, P. (2009). Membrane expansion alleviates endoplasmic reticulum stress independently of the unfolded protein response. J. Cell Biol. *187*, 525–536.

Settembre, C., Zoncu, R., Medina, D.L., Vetrini, F., Erdin, S., Erdin, S., Huynh, T., Ferron, M., Karsenty, G., Vellard, M.C., et al. (2012). A lysosome-to-nucleus signalling mechanism senses and regulates the lysosome via mTOR and TFEB. EMBO J. *31*, 1095–1108.

Starke, R.D., Ferraro, F., Paschalaki, K.E., Dryden, N.H., McKinnon, T.A., Sutton, R.E., Payne, E.M., Haskard, D.O., Hughes, A.D., Cutler, D.F., et al. (2011). Endothelial von Willebrand factor regulates angiogenesis. Blood *117*, 1071–1080.

Storrie, B., Micaroni, M., Morgan, G.P., Jones, N., Kamykowski, J.A., Wilkins, N., Pan, T.H., and Marsh, B.J. (2012). Electron tomography reveals Rab6 is essential to the trafficking of trans-Golgi clathrin and COPI-coated vesicles and the maintenance of Golgi cisternal number. Traffic *13*, 727–744.

Valentijn, K.M., Valentijn, J.A., Jansen, K.A., and Koster, A.J. (2008). A new look at Weibel-Palade body structure in endothelial cells using electron tomography. J. Struct. Biol. *161*, 447–458.

van Agtmaal, E.L., Bierings, R., Dragt, B.S., Leyen, T.A., Fernandez-Borja, M., Horrevoets, A.J., and Voorberg, J. (2012). The shear stress-induced transcription factor KLF2 affects dynamics and angiopoietin-2 content of Weibel-Palade bodies. PLoS ONE *7*, e38399.

van Galen, K.P., Tuinenburg, A., Smeets, E.M., and Schutgens, R.E. (2012). Von Willebrand factor deficiency and atherosclerosis. Blood Rev. *26*, 189–196.

Wagner, D.D., Mayadas, T., and Marder, V.J. (1986). Initial glycosylation and acidic pH in the Golgi apparatus are required for multimerization of von Willebrand factor. J. Cell Biol. *102*, 1320–1324.

Wagner, D.D., Saffaripour, S., Bonfanti, R., Sadler, J.E., Cramer, E.M., Chapman, B., and Mayadas, T.N. (1991). Induction of specific storage organelles by von Willebrand factor propolypeptide. Cell *64*, 403–413.

Weibel, E.R., and Palade, G.E. (1964). New cytoplasmic components in arterial endothelia. J. Cell Biol. *23*, 101–112.

Weidman, P., Roth, R., and Heuser, J. (1993). Golgi membrane dynamics imaged by freeze-etch electron microscopy: views of different membrane coatings involved in tubulation versus vesiculation. Cell *75*, 123–133.

Witte, K., Schuh, A.L., Hegermann, J., Sarkeshik, A., Mayers, J.R., Schwarze, K., Yates, J.R., 3rd, Eimer, S., and Audhya, A. (2011). TFG-1 function in protein secretion and oncogenesis. Nat. Cell Biol. *13*, 550–558.

Yadav, S., Puri, S., and Linstedt, A.D. (2009). A primary role for Golgi positioning in directed secretion, cell polarity, and wound healing. Mol. Biol. Cell *20*, 1728–1736.

Yoshida, T., Kamiya, T., Imanaka-Yoshida, K., and Sakakura, T. (1999). Low cytoplasmic pH causes fragmentation and dispersal of the Golgi apparatus in human hepatoma cells. Int. J. Exp. Pathol. *80*, 51–57.

Zenner, H.L., Collinson, L.M., Michaux, G., and Cutler, D.F. (2007). High-pressure freezing provides insights into Weibel-Palade body biogenesis. J. Cell Sci. *120*, 2117–2125.

Zhou, Y.F., Eng, E.T., Nishida, N., Lu, C., Walz, T., and Springer, T.A. (2011). A pH-regulated dimeric bouquet in the structure of von Willebrand factor. EMBO J. *30*, 4098–4111.

**Developmental Cell, Volume *29***

**Supplemental Information**

# A Two-Tier Golgi-Based Control of Organelle Size Underpins the Functional Plasticity of Endothelial Cells

**Francesco Ferraro, Janos Kriston-Vizi, Daniel J. Metcalf, Belen Martin-Martin, Jamie Freeman, Jemima J. Burden, David Westmoreland, Clare E. Dyer, Alex E. Knight, Robin Ketteler, and Daniel F. Cutler**

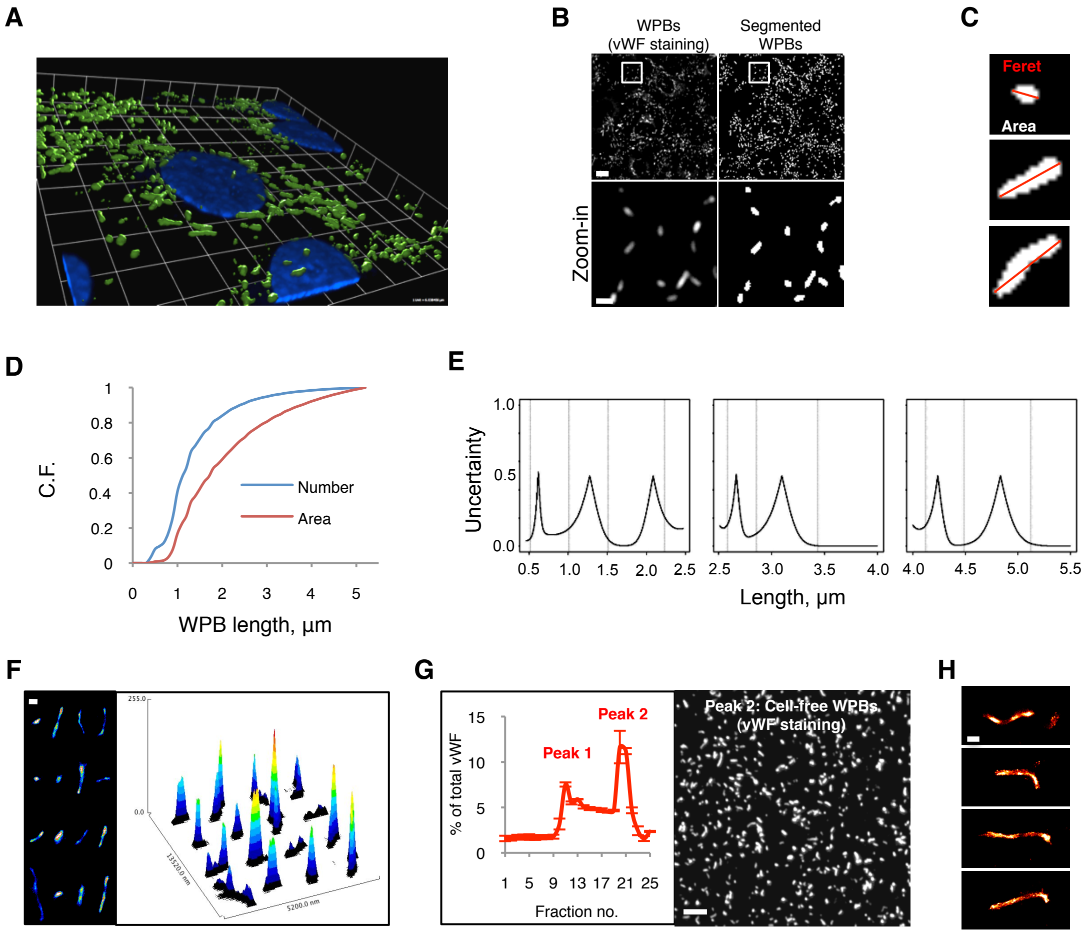
A
B
WPBs
(vWF staining)
Segmented
WPBs
Zoom-in
C
Feret
Area
D
C.F.
1
0.8
0.6
0.4
0.2
0
0
1
2
3
4
5
WPB length, µm
Number
Area
E
Uncertainty
1.0
0.5
0.0
0.5
1.0
1.5
2.0
2.5
2.5
3.0
3.5
4.0
4.0
4.5
5.0
5.5
Length, µm
F
G
15
10
5
0
% of total vWF
Peak 1
Peak 2
1
5
9
13
17
21
25
Fraction no.
Peak 2: Cell-free WPBs
(vWF staining)
H

Figure S1

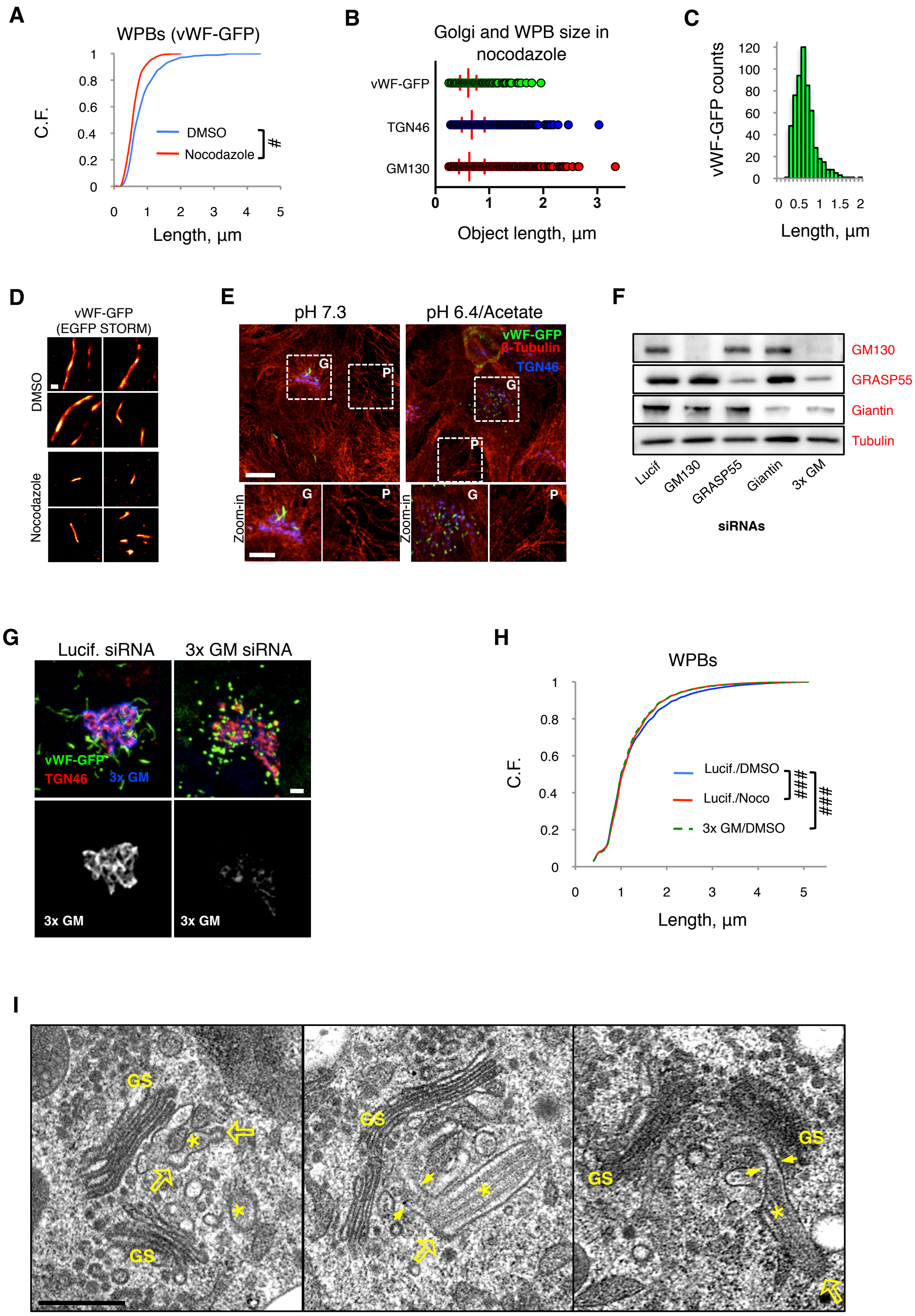
A
WPBs (vWF-GFP)
C.F.
DMSO
Nocodazole
#
Length, µm
B
Golgi and WPB size in nocodazole
vWF-GFP
TGN46
GM130
Object length, µm
C
vWF-GFP counts
Length, µm
D
vWF-GFP (EGFP STORM)
DMSO
Nocodazole
E
pH 7.3
pH 6.4/Acetate
vWF-GFP
β-Tubulin
TGN46
Zoom-in
F
GM130
GRASP55
Giantin
Tubulin
Lucif
GM130
GRASP55
Giantin
3x GM
siRNAs
G
Lucif. siRNA
3x GM siRNA
vWF-GFP
TGN46
3x GM
H
WPBs
C.F.
Lucif./DMSO
Lucif./Noco
3x GM/DMSO
###
###
Length, µm
I
GS

# Figure S2

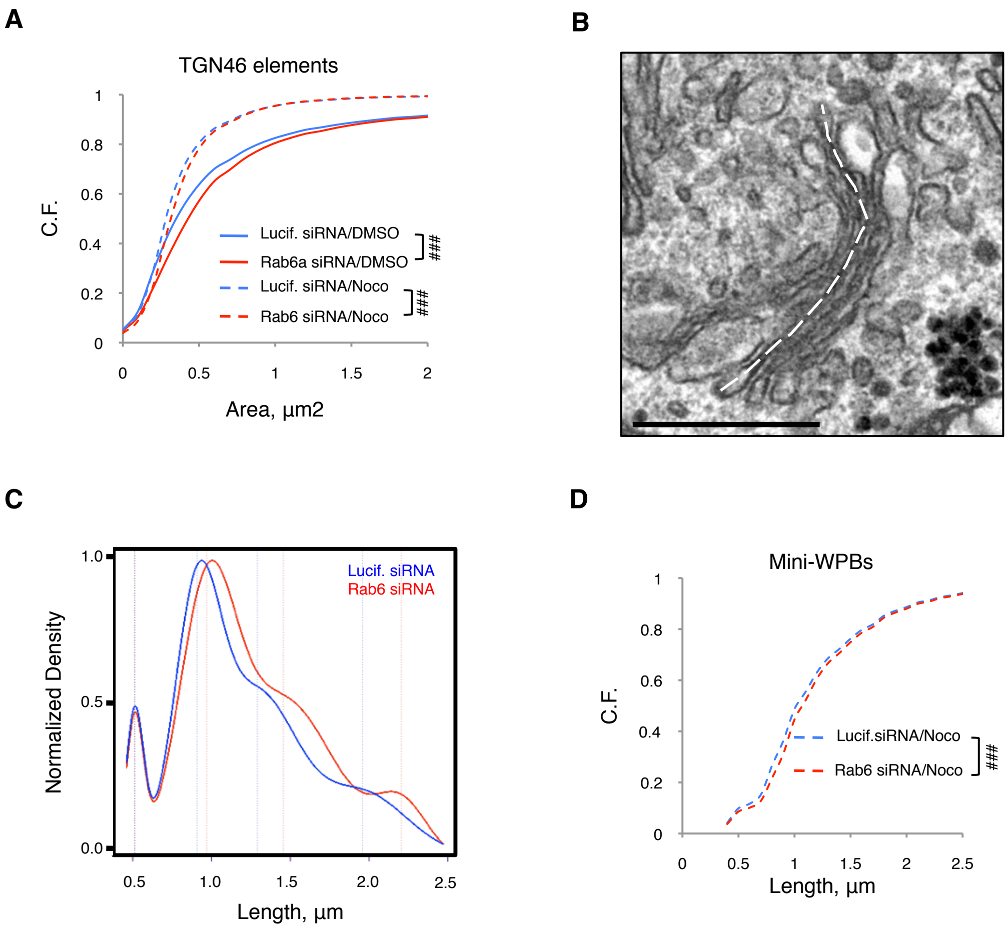
A
TGN46 elements
C.F.
Area, µm2
Lucif. siRNA/DMSO
Rab6a siRNA/DMSO
Lucif. siRNA/Noco
Rab6 siRNA/Noco
###
###
B
C
Normalized Density
Length, µm
Lucif. siRNA
Rab6 siRNA
D
Mini-WPBs
C.F.
Length, µm
Lucif.siRNA/Noco
Rab6 siRNA/Noco
###

**Figure S3**

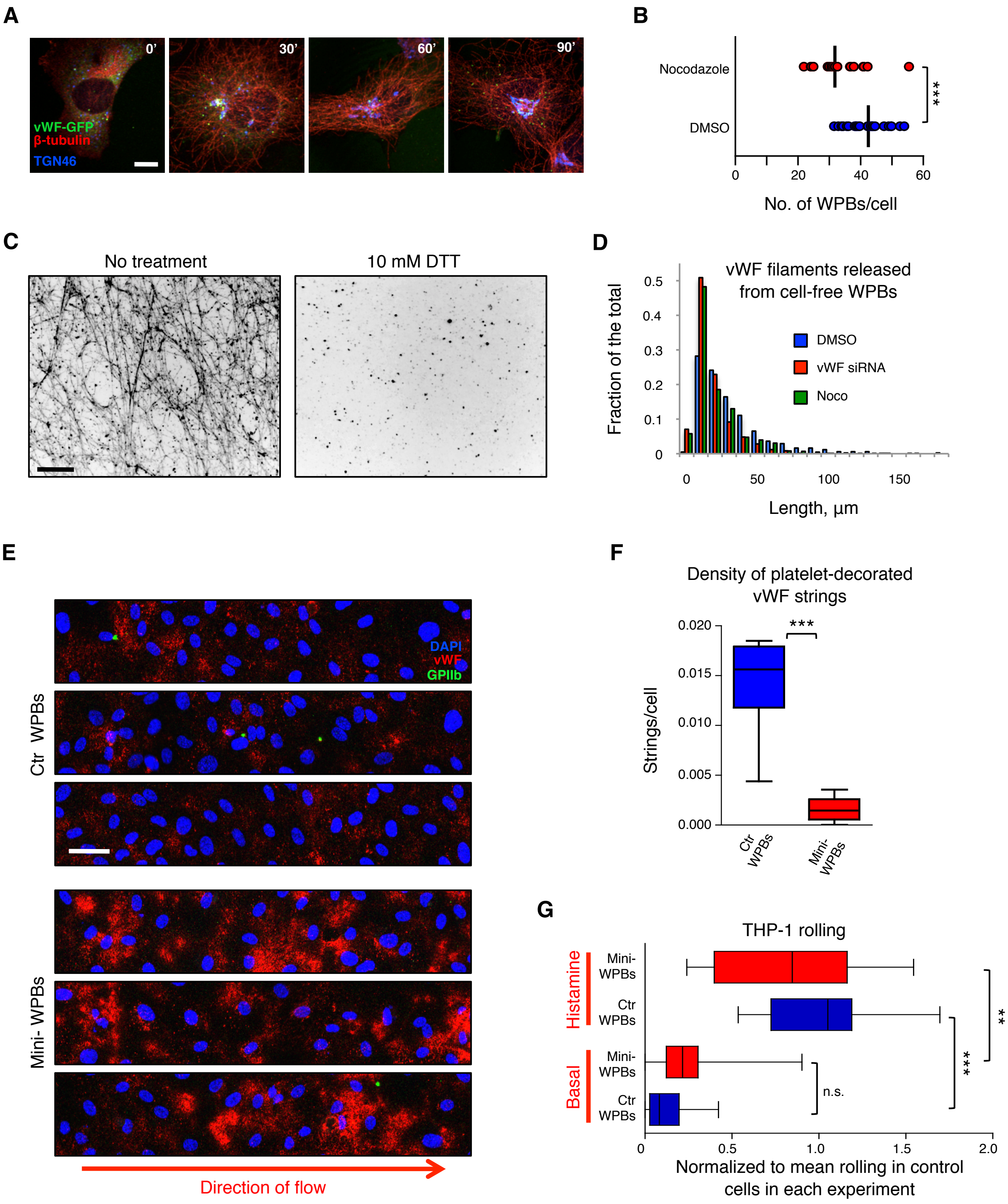
A
0'
30'
60'
90'
vWF-GFP
β-tubulin
TGN46
B
Nocodazole
DMSO
***
0
20
40
60
No. of WPBs/cell
C
No treatment
10 mM DTT
D
vWF filaments released
from cell-free WPBs
DMSO
vWF siRNA
Noco
Fraction of the total
0.5
0.4
0.3
0.2
0.1
0
0
50
100
150
Length, μm
E
DAPI
vWF
GPIIb
Ctr WPBs
Mini- WPBs
Direction of flow
F
Density of platelet-decorated
vWF strings
Strings/cell
0.020
0.015
0.010
0.005
0.000
***
Ctr WPBs
Mini-WPBs
G
THP-1 rolling
Histamine
Basal
Mini-WPBs
Ctr WPBs
Mini-WPBs
Ctr WPBs
**
***
n.s.
0
0.5
1.0
1.5
2.0
Normalized to mean rolling in control
cells in each experiment

**Figure S4**

A

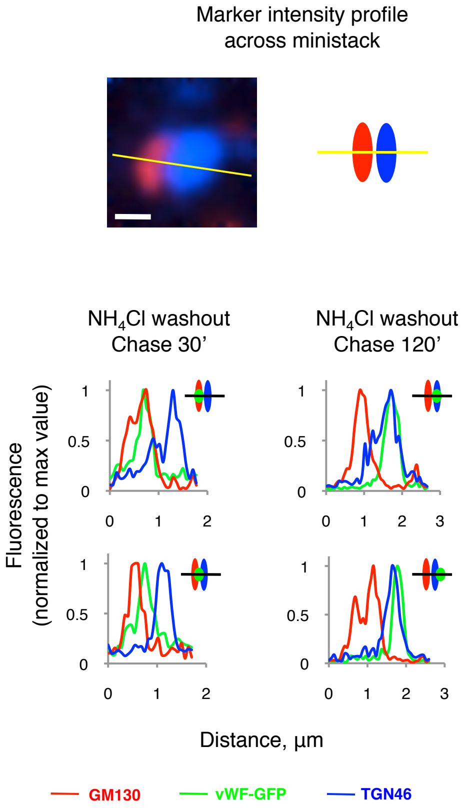
Marker intensity profile
across ministack
NH4Cl washout
Chase 30'
NH4Cl washout
Chase 120'
Fluorescence
(normalized to max value)
Distance, µm
GM130
vWF-GFP
TGN46

B

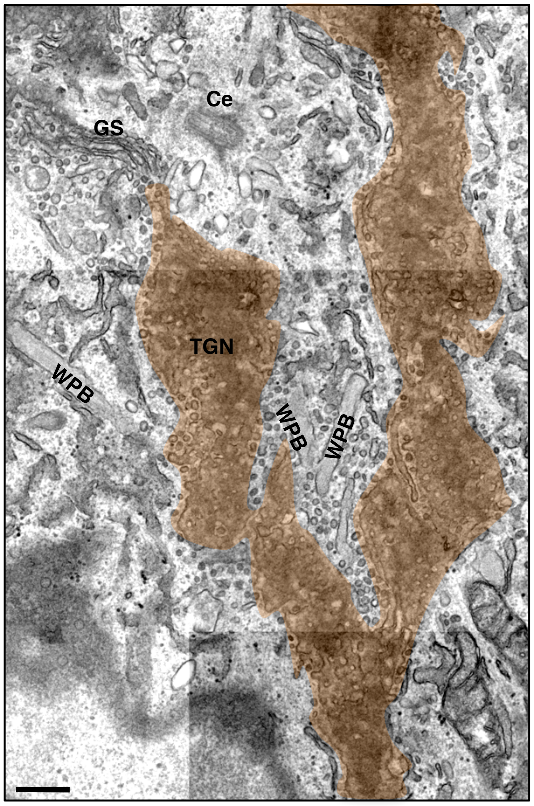
Ce
GS
TGN
WPB
WPB
WPB

**Figure S5**

**Figure S1.** Related to Figure 2. **High-throughput confocal morphometry and STORM imaging of WPBs**. (A) 3D-rendering of a confocal image stack of endothelial cells in which vWF was stained to label WPBs. Endothelial cell flatness forces WPBs to orient parallel to the substrate. This spatial constraint ensures that WPB lengths measured from 2D confocal images are a good approximation of actual organelle lengths. (B) High-throughput imaging and WPB segmentation. Top panels; left, a representative image acquired by the Opera LX confocal microscope; right, the same image after WPB segmentation (see Supplemental Experimental Procedures). Bottom panels; magnification of the insets shows that segmentation results in accurate identification of WPBs. Scale bar, 10 µm; zoom-in, 2 µm. (C) Two morphological parameters were measured. The "Feret diameter" (red lines) is the maximum distance between two points along the perimeter of one object and approximates the length of a WPB. The area is the sum of the pixels in the segmented object. (D) WPB number and area from the HTM survey data (cumulative frequencies plotted as function of organelle length). Long WPBs represent a minority of the total population (WPBs ≥ 3 µm are ~ 5%), but contain a significant amount of vWF (WPBs ≥ 3 µm account for ~ 20% of the total area covered by the segmented organelles). (E) The uncertainty in the attribution of any object (WPB) to two neighbouring length clusters in the HTM survey dataset. Low uncertainties corresponded to the density maxima identified by the Gaussian mixture modelling shown in Figure 2B (and indicated by the vertical lines), further supporting the presence of length clusters. (F) A montage of WPBs from vWF STORM images. Intensity values represent the number of accepted fluorophore localizations assigned to each pixel (20 nm x 20 nm), with 1 localization corresponding to 1 grey-scale intensity value. Intensities were color-coded for display purposes; on the right, intensity surface plots are shown. (G) Cell-free WPBs were obtained by centrifugation of HUVEC post-nuclear supernatants on discontinuous nycodenz gradients (see Extended Experimental Procedures). Peak 2 membranes, with the highest content of vWF, were adsorbed on Maxisorp™ surface (NUNC) by gentle centrifugation, fixed and processed for vWF immuno-fluorescence. Cell-free

WPBs maintain their typical elongated shape; scale bar, 10 µm. (H) STORM of cell-free WPBs centrifuged on coverslips to obtain 2D-constrained and well-separated organelles; scale bar, 500 nm.

**Figure S2.** Related to Figure 4**. Unlinked ministacks generate mini-WPBs**. (A) Cumulative frequency of the number of newly made WPBs (vWF-GFP labelled) as a function of organelle length. n: DMSO, 853; Nocodazole, 575. More than 95% of the WPBs produced by unlinked ministacks were ≤ 1 µm. #, $p < 10^{-10}$. (B) Lengths of Golgi elements (GM130- and TGN46-positive structures) and WPBs produced during nocodazole treatment. Medians and interquartile ranges are shown. n = 1530 and 1157 for GM130- and TGN46-positive objects, respectively. (C) Length frequency of vWF-GFP labelled WPBs generated by nocodazole treatment. (D) vWF-GFP STORM images (anti-EGFP antibody) of cells treated as in (A); scale bar, 500 nm. (E) Low pH/acetate treatment unlinks the Golgi ribbon without affecting the microtubule network (G, Golgi region; P, cell periphery). Scale bar, 10 µm; zoom-in, 5 µm. (F) The efficiency of individual or combined siRNA-mediated depletion of the three Golgi matrix proteins GM130, GRASP55 and Giantin was in the range of 80-95%. (G) Newly made WPBs (vWF-GFP) in control and triple GM130, GRASP55, Giantin knockdown (3x GM) in HUVECs. The Golgi matrix proteins were simultaneously stained with a mix of three specific antibodies; scale bar, 2 µm. (H) HTM analysis showed that 3x GM knockdown was as efficient as nocodazole in generating mini-WPBs. n = $1.27 \times 10^6$, $7.16 \times 10^5$ and $7.46 \times 10^5$ for Lucif./DMSO, 3xGM/DMSO and Lucif./Noco, respectively. ###, $p < 10^{-15}$. (I) Mini-WPBs generated by unlinked Golgi ministacks (nocodazole incubation) were ultrastructurally normal. Ministacks (GS), clathrin coats (hollow arrows), membrane continuities with the TGN (arrows) and vWF tubules (asterisks) are indicated; scale bar, 500 nm.

**Figure S3.** Related to Figure 4. **Effects of Rab6a/a' depletion on Golgi and WPB sizes.** (A) HTM analysis of the area of the TGN46-positive objects analyzed in Figure 4J. ###, $p < 10^{-15}$. (B) Golgi ribbons were unlinked with nocodazole into separated ministacks in order to facilitate cisternal length measurement from electron micrographs as previously described by (Trucco et al., 2004); scale bar, 500 nm. (C) Gaussian mixture models of WPB length clusters of Luciferase and Rab6a/a' siRNA-treated HUVECs. Rab6a/a' depletion shifted the clusters toward higher values. (D) HTM analysis of the length of nocodazole-induced mini-WPBs in Luciferase and Rab6a/a' siRNA-treated HUVECs. ###, $p < 10^{-15}$.

**Figure S4.** Related to Figure 6. **Size-dependent properties of WPBs *in vitro* and in cells.** (A) After nocodazole washout, the microtubule network reforms by 30 min, while Golgi ribbon reassembly is completed by 90 min; scale bar, 10 μm. (B) HTM analysis of WPB number per cell. Nocodazole treatment induced a minor reduction of the number of WPBs per cell at steady state. Median values are shown (n = 24; with each observation from a separate well of 96-well plates); ***, $p < 10^{-3}$. (C) Cell-free WPBs were treated in solution with control buffer or buffer supplemented with 10 mM dithiothreitol (DTT) to reduce multimer disulfide bonds. Treated WPBs were adsorbed by centrifugation on plastic, permeabilized and fixed. vWF filaments production was ablated by DTT incubation, indicating that vWF multimers are required for their formation. Inverted wide-field images are shown. Scale bar, 50 μm. (D) The frequency of filaments ≤ 20 μm produced by cell-free mini-WPBs was greatly increased. (E) Platelet-decorated vWF strings were not formed in the absence of secretagogue. In agreement with their increased levels of basal secretion (see Figure 6A), mini-WPB producing cells displayed higher vWF surface staining. Scale bar, 50 μm. (F) Density (number/HUVEC) of vWF-decorated platelet strings in samples stimulated with histamine. Box plots of data of 8 tile scans per treatment (with ~ 4 $mm^2$/tile scan) from 2 separate experiments; ***, $p < 10^{-3}$. (G) Basal and histamine-stimulated

rolling of THP-1 monocyte-like cells for the same experiments shown in Figure 6F. Histamine increased the rolling of monocytes to similar extent in control and mini-WPB enriched HUVECs. Note that there was no difference in basal levels of rolling between the treatments. Box plots of 12 determinations from 4 experiments; **, $p < 10^{-2}$; ***, $p < 10^{-3}$.

**Figure S5.** Related to Figure 7**. Ministacks and TGN in WPB biogenesis.** (A) $NH_4Cl$ incubation/washout protocol was carried out in cells where the Golgi ribbon had been unlinked by nocodazole treatment. This allowed localization of vWF-GFP within the ministacks during WPB formation. Marker intensity across ministacks was measured in ImageJ with the “plot profile” tool (as shown for the confocal image, scale bar 0.5 μm, and exemplified by the diagram). Two examples each are reported for 30 and 120 min post $NH_4Cl$ washout. Consistent with vWF quantum formation in the cisternae of the ministacks, at 30 min (early in WPB formation process; see Figures 3C-3E), vWF-GFP objects could be observed at locations corresponding to the cis- and median/trans-Golgi (shown plots) and the TGN (not shown). At 120 min (before WPB budding; see Figures 3C-3E), the most common location of vWF-GFP objects was at or protruding from the TGN. (B) EM Montage of the Golgi region of a HUVEC containing a portion of the TGN and seen “en face” (outlined by a light brown transparent mask). The continuity of the TGN is at the base of our model of VWF quanta co-packaging into forming WPBs. A ministack, connected to the ribbon and captured in transverse version (GS), a centriole (Ce) and WPBs are visible. Scale bar, 500 nm.

**Table S1.** Related to Figure 2.

| WPB length cluster | Range of Feret diameter analyzed | Cluster mean length (µm) | Cluster standard deviation (µm) |
|---|---|---|---|
| 1 | < 2.5 µm | 0.5 | 0.05 |
| 2 | < 2.5 µm | 1 | 0.18 |
| 3 | < 2.5 µm | 1.5 | 0.33 |
| 4 | < 2.5 µm | 2.2 | 0.15 |
| 5 | 2.5-4.0 µm | 2.6 | 0.06 |
| 6 | 2.5-4.0 µm | 2.9 | 0.16 |
| 7 | 2.5-4.0 µm | 3.4 | 0.3 |
| 8 | 4.0-5.5 µm | 4.1 | 0.08 |
| 9 | 4.0-5.5 µm | 4.5 | 0.21 |
| 10 | 4.0-5.5 µm | 5.1 | 0.22 |

**Regularly spaced WPB length clusters.**

WPB lengths cluster at values multiple of ~ 0.5 µm. Estimated means and standard deviations of the individual components of the Gaussian mixture (Figure 2B) calculated by Mclust.

**Table S2.** Related to Figure 5.

| Length interval (μm) 1.4-2.5 | 1st cluster mean (μm) | 1st cluster St. Dev. (μm) | 2nd cluster mean (μm) | 2nd cluster St. Dev. | no. of WPBs in interval |
|---|---|---|---|---|---|
| Control siRNA 200 pmol | 1.55 | 0.09 | 1.97 | 0.25 | 212857 |
| vWF siRNA 10 pmol | 1.55 | 0.08 | 1.95 | 0.25 | 79580 |
| vWF siRNA 50 pmol | 1.54 | 0.08 | 1.92 | 0.25 | 45182 |
| vWF siRNA 200 pmol | 1.54 | 0.08 | 1.90 | 0.24 | 16095 |
| **Length interval (μm) 1.9-3.1** | **1st cluster mean (μm)** | **1st cluster St. Dev. (μm)** | **2nd cluster mean (μm)** | **2nd cluster St. Dev. (μm)** | **no. of WPBs in interval** |
| Control siRNA 200 pmol | 2.06 | 0.10 | 2.52 | 0.27 | 107686 |
| vWF siRNA 10 pmol | 2.06 | 0.09 | 2.50 | 0.27 | 34357 |
| vWF siRNA 50 pmol | 2.04 | 0.08 | 2.46 | 0.27 | 5613 |
| vWF siRNA 200 pmol | 2.06 | 0.08 | 2.50 | 0.27 | 456 |

**vWF expression levels do not affect quantum size.**

Estimated means and standard deviations of the two components of the Gaussian mixture calculated by Mclust for each siRNA treatment in the length intervals shown in Figure. 5E.

EXTENDED EXPERIMENTAL PROCEDURES

**HUVEC growth medium (HGM).** HUVECs were maintained in HGM, M199 (Gibco, Life Technologies) supplemented with 20% Fetal Bovine Serum, (Biosera), 30 µg/ml endothelial cell growth supplement from bovine neural tissue and 10 U/ml Heparin (both from Sigma-Aldrich).

**Antibodies and reagents.** Anti-vWF rabbit polyclonal (cat. no. A0082), rabbit-polyclonal HRP-conjugated (cat. no. P0226), and mouse monoclonal (clone F8/86), were from DAKO. Anti-GM130 mouse monoclonal, clone 35, was from BD Biosciences. Anti-TGN46 sheep polyclonal AHP500 was from AbD Serotec. Anti-GRASP55 rabbit polyclonal was from Proteintech. Anti-Giantin rabbit polyclonal (cat. no. Ab24568) was from Abcam. Anti-Rab6 rabbit polyclonal C-19 was from Santa Cruz Biotechnology. Anti-β-Tubulin mouse monoclonal (clone TUB 2.1) was from Sigma Aldrich. Anti-pan-Tubulin sheep polyclonal ATN02 was from Cytoskeleton. Anti-EGFP (mixture of two mouse monoclonal antibodies) was from Roche. Anti-CD41 mouse monoclonal (clone 5B12) was from Millipore. All reagents were from Sigma-Aldrich unless otherwise specified.

**Plasmids and siRNAs.** siRNA were custom synthesized (Eurofins MWG Operon). Targets and siRNA sequences:
*Firefly Luciferase*, sense, 5'-CGUACGCGGAAUACUUCGA[dT][dT]-3';
*hvWF*, sense, 5'-GGGCUCGAGUGUACCAAAA[dT][dT]-3';
*hGM130*, sense, 5'- AAGUUAGAGAUGACGGAACUC [dT][dT]-3';
*hGRASP55*, sense, 5'- AACUGUCGAGAAGUGAUUAUU[dT][dT]-3'
*hGiantin*, sense, 5' - AACUUCAUGCGAAGGCCAAAU [dT][dT]-3';
*hRab6a/a'*, sense, 5'- GAGAAGAUAUGAUUGACAU[dT][dT] (targets both Rab6a and Rab6a' isoforms). All siRNAs were described and validated in other studies (Elbashir et al., 2001; Feinstein and Linstedt, 2008; Heuer et al., 2009; Puthenveedu et al., 2006; Starke et al., 2011; Sun et al., 2007). Plasmids encoding vWF-GFP, a gift from J. Voorberg and J.A. Van Mourik, and EGFP-Rab27A were previously described (Hannah et al., 2003; Romani de Wit et al.,

2003). Plasmid DNA was nucleofected at 4 to 7.5 µg per reaction. Cells treated with siRNAs targeting vWF and Luciferase were analyzed at 48 except in the case of time course experiments. siRNAs were delivered at 200 pmol per reaction or as indicated together with EGFP-Rab27A plasmid when the latter was used. siRNAs to GM130, GRASP55, Giantin, Rab6a/a' (and Luciferase as control) were used at 1000 pmol per reaction and a second round of nucleofection at 48 h was carried out. Cells were analyzed 24 h after the second nucleofection round. When vWF-GFP was used to label newly made WPBs, it was nucleofected at the time of the second round.

**Stochastic Optical Reconstruction Microscopy (STORM).** Fluorescence and excitation lights were spectrally separated by a full multi-edge filter set (LF405/488/561/635-A-000, Semrock). A diode laser operating at 640 nm (iBeam smart, 150mW, Toptica Photonic AG) was fibre coupled, expanded and focused at the edge of the back focal plane of the objective (UAPON 100xOTIRF, NA=1.49). The beam size of the excitation laser was matched to the field of view of the imaging system limited by the detector chip size (16μm/pixel*512pixel). The power density of the excitation laser on the sample was approximately 2 kW/cm$^2$. For data acquisition, a ROI (128×128 pixels) with the highest and most homogeneously illuminated area was used. Image stack files of fluorophore "blinking" events (composed of 10000 to 20000 frames) were captured using a low-noise, highly sensitive electron-multiplying EMCCD camera (Andor iXon 897, Belfast, UK) at frames rates of 54 per second with 10 ms exposure times. Fluorophores (AlexaFluor 647 or Cy5) were reactivated by a 405 nm laser (Mitsubishi Electronics Corp.) at low power density (<50 W/cm$^2$). Samples were imaged in a photo-switching buffer containing 50 µg/ml (5 units) glucose oxidase, 1 µg/ml (40-60 units) catalase and 100 mM mercaptoethylamine-HCl (Metcalf et al., 2013). Images were reconstructed using the rainSTORM algorithm (http://laser.cheng.cam.ac.uk/wiki/index.php/Resource (Metcalf et al., 2013; Rees et al., 2012), with super-resolution pixel sizes of 20 nm. Mean localization precisions were around 25 nm, giving an estimated resolution limit of 50-60 nm

(Rees et al., 2012). STORM data is displayed by histogram visualization; i.e. accepted localizations were assigned to 20 nm pixels, each localization corresponding to 1 grey scale intensity value. For clarity of viewing a "red hot" lookup table and a contrast enhancement were applied (0.01-0.1% pixel saturation) in ImageJ.

**Image analysis**. *Confocal microscopy*. Maximum intensity projections were analyzed in ImageJ (http://imagej.nih.gov/ij/). Background intensity in vWF-GFP channel was measured and used for threshold setting. The "Find Maxima" tool was used to identify positive objects and the image subjected to watershed segmentation, separating nearby objects. Segmentation was then superimposed on the binary image obtained after threshold application. Selection of a series of objects was carried out to identify the area of the smallest, which was set as a cut-off in the parameter of the "Analyze Particles" tool. The same procedure was applied for quantifications of Golgi compartments (i.e. GM130- and TGN46-positive objects).

*STORM*. Reconstructed images were analysed in ImageJ. vWF quanta were identified using the "Find maxima" tool and separated by watershed segmentation. Segmentation was applied to binary images of the reconstructions to separate the quanta within the same organelle. The "Analyze Particles" tool was used to measure the Feret diameter of each separated quantum.

**High-throughput confocal microscopy and morphometric analysis (High-throughput morphometry, HTM)**. A high performance barebone computer system equipped with 256 GB RAM and four dodeca-core CPUs running at 2.2 GHz, allowing parallelization of computation, was used for image processing. The system was running a 64-bit version of Ubuntu Linux. Image processing was performed ImageJ program (Abramoff et al., 2004) version 1.45l (http://imagej.nih.gov/ij/) with Java 1.6.0 using 111 gigabytes of heap size and enabling the incremental Java garbage collector. Flex format image files were converted to Tagged Image Format (TIF). The processing steps were executed

as a custom-built ImageJ macro, processing a 96-well plate image dataset in a single ImageJ instance. The macro performed the following steps.

*Pre-processing*. Images of a whole 96 well plate were imported in ImageJ as an image sequence resulting in a 16-bit stack. The stack was converted into a multi-channel hyperstack. The pixel width and height was calibrated to 0.1615 μm (40x lens with no camera binning). The vWF channel (labelling Weibel-Palade bodies) was subjected to noise filtering. Endoplasmic reticulum vWF pool was an inherent source of noise and was removed by applying a rolling ball algorithm (Sternberg, 1983) with a paraboloid curvature radius of 1 or 5 pixels depending of the noise level.

*Segmentation*. The segmentation process of WPBs had to be able to (i) find contours of both strongly and weakly fluorescent WPBs and (ii) separate closely apposed WPBs. Global segmentation algorithms that calculate a single threshold for a whole image were not amenable to these criteria. Instead, a local segmentation was applied where threshold was calculated as a function of the coordinates at each pixel. The ImageJ implementation of the Bernsen (Bernsen, 1986) locally adaptive threshold algorithm is based on the local contrast around a central pixel. We used an experimentally determined 5-pixel radius circular window around the central pixel, as it was found to be the optimal parameter with respect to WPB size. The default contrast threshold level (set at 15) was used. An overlay image was generated automatically for quality control purpose for every segmented WPB image, where WPB contours were superimposed on the original image. The feature extraction step in the high-content analysis workflow allowed us to reduce the image dataset stored as pixels to more geometrically interpretable measurements, such as WPB area and Feret diameter (also known as maximum caliper), which is the longest distance between any two opposite points along the perimeter of an object. In the case of a WPB this distance approximates its major axis, and therefore its length. Despite reducing the dataset magnitude from hundreds of gigabytes to megabytes, this still led to a multidimensional space where a WPB was described by 22 measured values. During the feature selection step, area and Feret diameter were chosen to be the

most salient features to define a WPB. The objects thus identified were filtered based on size, cutting off those with sizes ≤ 2 pixels (approximately 300 nm), which visually did not conform to WPB morphology and likely represent vWF vesicular carriers.

*Statistical data analysis*. For the initial survey, WPB objects were measured (n = 1976161, from cells grown in 432 wells of 96-well plates) and the quantified features were saved into large text files. We employed the open source statistical and analytical tool ***R*** (http://r-project.org) (Team, 2009) for data analysis. Visual inspection indicated that WPB length frequency distribution was multi-modal, presenting local maxima (that is, preferred lengths or length clusters). We hypothesized that WPB length distribution could be modelled by a mixture of Gaussian functions. To estimate the positions of the length clusters, we used model-based clustering to fit a Gaussian mixture model as a density estimate for our dataset. Using the ***R*** package “Mclust” version 4 (Fraley and Raftery, 2002) that fits a mixture of Gaussian functions, we performed cluster analysis and calculated the univariate density and uncertainty. The assumed number of mixture components (clusters) of the Mclust function were chosen as G = 4, which was found as the optimal number of mixture components with unequal variance model for lengths < 2.5 μm. G = 3 with unequal variance model was optimal for the 2.5-4.0 μm and 4.0-5.5 μm length intervals. All other parameters were used as default. The mean and standard deviation of each cluster were calculated (see Supplementary Table 1). The ‘mclust1Dplot’ function was also used to calculate and plot, from the original length dataset, the density and uncertainty (misclassification) for value attribution to neighbouring clusters. The most uncertain (misclassified) values were located at length intermediate between two clusters. ***R*** was also used to analyze all the other experimental high-throughput imaging datasets.

Golgi (TGN46-positive) elements were subjected essentially to the same processing and segmentation as described for WPBs. Lengths (Feret diameters) and areas of the segmented Golgi elements were analyzed with ***R***.

**$NH_4Cl$ incubation/washout.** HUVECs were treated with 10 mM $NH_4Cl$ in growth medium for the times described to prevent generation of WPBs at the Golgi (Wagner et al., 1986). Resumption of WPB formation was obtained washing out $NH_4Cl$ by extensive rinsing with ice-cold HGM or serum-free (SF) medium (M199 supplemented with 10 mM HEPES-NaOH, pH 7.4 and 0.1 mg/ml BSA, followed by incubation in media at 37 °C for the indicated times.

**Golgi ribbon unlinking.** Cells were incubated with nocodazole, diluted in HGM to 1 µg/ml (from a 10 µg/ml stock in DMSO) for 18-20 h. HGM supplemented with DMSO (0.01% final concentration) was used as a control. In a second approach, we adopted the protocol described by (Yoshida et al. 1999) with slight modifications. HEPES-NaOH, pH 7.3, from a 1 M solution, was added to HGM (final pH of ~ 7.3) to a final concentration of 30 mM (control). 2-(N-Morpholino)ethanesulphonic acid (MES) and sodium acetate, both from 1 M stocks, were added to 30 and 25 mM final concentrations, respectively. The pH of this medium was ~ 6.4. Cells were grown in these media for 24 h before being processed for immuno-fluorescence.

**Golgi ribbon unlinking during WPB formation**. Cells were nucleofected with vWF-GFP and incubated with $NH_4Cl$ overnight as described above. To allow formation of WPBs, $NH_4Cl$ was washed out and cells were chased with medium supplemented with DMSO. To unlink the Golgi ribbon at the indicated times, cells were transferred on ice for 30 min to depolymerize microtubules and then chased in nocodazole at 37 ºC for the indicated times before being processed for immuno-fluorescence.

**vWF imaging at the Golgi during WPB formation.** Cells were nucleofected with vWF-GFP and incubated overnight with both $NH_4Cl$ and nocodazole at the concentrations described above. $NH_4Cl$ was then washed out and cells chased for the indicated times in the continued presence of nocodazole before being processed for immuno-fluorescence. Fluorescence maxima for vWF-GFP and

the Golgi markers GM130 (cis-Golgi) and TGN46 (TGN) were identified with ImageJ's "plot profile" tool, in order to localize the WPB marker in the context of the ministacks.

**Western blotting.** Cells were lysed on ice with the following buffer: 100 mM Tris-HCl, pH 7.5/150 mm NaCl/1% TX-100/0.5% Na-Deoxycholate/0.05% SDS, supplemented with protease inhibitor cocktail (Sigma-Aldrich). Lysates were clarified by centrifugation and protein concentration measured by bicinchoninic acid assay (Pierce). Equal protein amounts were fractionated by SDS-PAGE in denaturing and reducing conditions and electro-blotted on PVDF membranes. After blocking with 5% BSA in 0.05% Tween-20 PBS (PBS-T), membranes were incubated with primary antibodies diluted in 5% BSA/PBS-T, followed by HRP-conjugated secondary antibodies. Chemiluminescent signals were acquired in digital format with ImageQuant LAS 4000 (GE Healthcare Life Sciences) Signals were quantified in ImageJ; pan-tubulin signal was used for normalization.

**vWF ELISA and multimer analysis.** vWF content in cell lysates, releasates and fractions form gradients was measured using a sandwich ELISA, as previously described (Blagoveshchenskaya et al., 2002). vWF multimers were fractionated by agarose gel electrophoresis, electro-blotted on PVDF and detected as described in (Nightingale et al., 2009).

**Secretion assays.** After 5 rinses in SF medium (5 incubations, each for 3-4 min) cells were assayed in the same medium. vWF releasates in the absence (basal) or the presence of the secretagogue histamine (100 μM), each for 30 min at 37 ºC, were quantified by ELISA and expressed as percent of total vWF content (obtained by adding the amounts of vWF in lysates and releasates). Cells treated with DMSO and nocodazole were subjected to washout 1 h before assay to allow complete reassembly of the microtubule network.

**Preparation of cell-free WPBs**. HUVECs were homogenized on ice in 250 mM sucrose/5 mM $Na_2$-EDTA/20 mM HEPES-NaOH, pH 7.4, supplemented with protease inhibitor cocktail. Homogenates were centrifuged at 1000 g for 5 min (at 4 °C). The resulting in post-nuclear supernatants were loaded on 15%-40% Nycodenz (Axis-Shields) cushions (both in homogenization buffer) and centrifuged at 18000 x g and 4 °C for 30 min with MLS50 (Beckman Coulter) in an Optima™ Max ultracentrifuge. Cell-free WPBs were recovered at the 15%-40% Nycodenz interface.

**vWF filaments generated from cell-free WPBs**. vWF-silenced (for 48 h), DMSO and nocodazole treated (both for 18-20 h) HUVECs were processed to obtain cell-free WPBs as described. Fractions containing WPBs (equal vWF amounts by ELISA) were serially diluted in 96-well Maxisorp™ plates (NUNC, Thermo Scientific) and centrifuged (2000 r.p.m. with a GH3.8 rotor, Beckman Coulter) for 30 min at 4 °C. Surface-adsorbed membranes were permeabilized (30 min on ice with 1% Triton X-100 in homogenization buffer) and fixed with 4% formaldehyde in the same buffer. After immuno-staining, vWF filaments were imaged by wide-field microscopy with a 20x objective lens (NA 0.4) on a Leica DM IRB microscope. vWF string lengths were measured using ImageJ's "segmented line" tool.

**Platelet-decorated vWF strings generation and analysis.** HUVECs were seeded in flow chambers (μ-Slide I, Ibidi) to produce confluent monolayers and then fed with growth medium supplemented with either vehicle (DMSO) or 1 μg/ml nocodazole to obtain control and mini-WPB enriched cells. After 20 h culture, cells were extensively rinsed to remove DMSO or nocodazole and incubated at 37 °C in growth medium for 45 minutes before being connected to a pump system (Harvard Apparatus, Holliston, MA, USA). Hanks buffer (containing $Ca^{2+}$, $Mg^{2+}$and 0.2 % BSA) pre-warmed at 37 °C was perfused for a few minutes to rinse out the growth medium and loosely attached cells. Then, the same buffer supplemented with 100 μM histamine was perfused for 1 min alone and then with

platelets (freshly prepared as described in (Michaux et al., 2006), at a concentration of 0.5-1 x $10^8$ per ml) for 5 min. A constant shear stress of 0.25 MPa (2.5 dynes/$cm^2$) was maintained throughout the experiment. In some experiments histamine was omitted throughout the perfusion in order to visualize the formation of strings in basal conditions. Cells were then fixed under flow with 4% formaldehyde in PBS for 5 min, gradually reducing the flow rate to zero. Cells were not permeabilized and extracellular pools of vWF (to visualize strings) and GPIIb/CD41 (to visualize platelets) were immuno-stained; DAPI (Life Technologies) was used to label the nuclei. Stacks of confocal images were acquired from 20 adjacent fields of view and stitched together into tilescans. Maximum intensity projections of the tilescans were used for quantification of length and density of vWF-decorated platelet string (i.e. number of strings/nuclei in each tilescan) with ImageJ, using the "straight line" tool. Platelet string length was defined as the maximum distance between platelets decorating an underlying vWF filament.

**Leukocyte rolling assays**. HUVECs (seeded on µ-slides VI, Ibidi) were essentially prepared as described for the platelet string assays. At the time of assay, flow chambers were transferred to the microscope stage of an Axiovert 100 (Carl Zeiss, Welwyn Garden City, UK) maintained at 37 °C and imaged using a 10x objective. After a brief perfusion with Hanks buffer (supplemented as described for the platelet string assays), THP-1 monocytes ($10^6$ cells/ml in Hanks buffer) were perfused at a shear stress of 0.07 Pa (0.7 dyne/$cm^2$) on the endothelial monolayers for a 3 min period to measure basal rolling. HUVECs were then perfused (in the absence of THP-1s) with buffer supplemented with 100 μM histamine for 5 min, followed by THP-1 ($10^6$ cells/ml) suspended in Hanks buffer devoid of secretagogue for a 3 min period. The rolling on the same field of view, in basal and stimulated conditions, was imaged by video-microscopy (at 24 frames/sec, using a QIMAGING Scientific CMOS Rolera bolt camera). Only THP-1 cells that rolled on the HUVEC monolayer at speeds ≤ 40 µm/sec were counted. To minimize the variability between experiments carried

out on different days, the number of rolling cells were normalized to the number of HUVECs imaged in the analyzed field of view and expressed as fraction of the mean rolling for histamine-stimulated controls in that day/experiment.

**3D reconstruction from fixed cells**. Confocal image stacks of fixed HUVECs, labelled with DAPI and antibodies to the appropriate organelle marker, were imported in Volocity (Pelkin Elmer) or IMARIS (Bitplane) for tri-dimensional rendering of nuclei, WPBs and Golgi regions.

**Live cell imaging**. HUVECs were nucleofected with vWF-GFP plasmid, seeded on gelatine coated glass bottom imaging dishes (PAA laboratories GmbH) and cultured overnight in growth medium. To label the Golgi Complex, cells were rinsed three times with serum-free medium and then incubated with 5 μM BODIPY Ceramide (Molecular Probes, Life Technologies) in serum-free medium for 10 min at 37 ºC. Cells were then washed three times in serum-free medium and incubated for 30 more min in growth medium at 37 ºC before imaging. Image acquisition was carried out with an Ultra*VIEW* VoX spinninig disk confocal system (Perkin Elmer) equipped with temperature and $CO_2$ controls (set at 37 ºC and 5%, respectively). Stacks of images (*z*-step, 0.25 μm), encompassing the entire cell volume, were sequentially acquired for the ceramide and the GFP channels at a rate of 1 stack every 10 sec for 20-150 min. Image sequences were imported in IMARIS (Bitplane Scientific Software) for object tracking and 3D-rendering.

## SUPPLEMENTAL REFERENCES


Abramoff, M.D., Magalhães, P.J., and Ram, S.J. (2004). Image processing with ImageJ. Biophotonics international *11*, 36-42.

Bernsen, J. (1986). Dynamic Thresholding of Grey-Level Images. Paper presented at: 8th Int Conf on Pattern Recognition (ICPR8) (Paris, France).

Blagoveshchenskaya, A.D., Hannah, M.J., Allen, S., and Cutler, D.F. (2002). Selective and signal-dependent recruitment of membrane proteins to secretory granules formed by heterologously expressed von Willebrand factor. Molecular biology of the cell *13*, 1582-1593.

Elbashir, S.M., Harborth, J., Lendeckel, W., Yalcin, A., Weber, K., and Tuschl, T. (2001). Duplexes of 21-nucleotide RNAs mediate RNA interference in cultured mammalian cells. Nature *411*, 494-498.

Fraley, C., and Raftery, A.E. (2002). Model-based clustering, discriminant analysis, and density estimation. J Am Stat Assoc *97*, 611-631.

Hannah, M.J., Hume, A.N., Arribas, M., Williams, R., Hewlett, L.J., Seabra, M.C., and Cutler, D.F. (2003). Weibel-Palade bodies recruit Rab27 by a content-driven, maturation-dependent mechanism that is independent of cell type. Journal of cell science *116*, 3939-3948.

Metcalf, D.J., Edwards, R., Kumarswami, N., and Knight, A.E. (2013). Test samples for optimizing STORM super-resolution microscopy. J Vis Exp.

Rees, J.E., Erdelyi, M., Pinotsi, D., Knight, A., Metcalf, D., and Kaminski, C.F. (2012). Blind assessment of localization microscope image resolution. Optical Nanoscopy *1*.

Romani de Wit, T., Rondaij, M.G., Hordijk, P.L., Voorberg, J., and van Mourik, J.A. (2003). Real-time imaging of the dynamics and secretory behavior of Weibel-Palade bodies. Arterioscler Thromb Vasc Biol *23*, 755-761.

Starke, R.D., Ferraro, F., Paschalaki, K.E., Dryden, N.H., McKinnon, T.A., Sutton, R.E., Payne, E.M., Haskard, D.O., Hughes, A.D., Cutler, D.F., *et al.* (2011). Endothelial von Willebrand factor regulates angiogenesis. Blood *117*, 1071-1080.

Sternberg, S.R. (1983). Biomedical Image Processing. Computer *16*, 22-34.

Sun, Y., Shestakova, A., Hunt, L., Sehgal, S., Lupashin, V., and Storrie, B. (2007). Rab6 regulates both ZW10/RINT-1 and conserved oligomeric Golgi complex-dependent Golgi trafficking and homeostasis. Molecular biology of the cell *18*, 4129-4142.

Team, R.D.C. (2009). R: A language and environment for statistical computing (Vienna, Austria, R Foundation for Statistical Computing).

Trucco, A., Polishchuk, R.S., Martella, O., Di Pentima, A., Fusella, A., Di Giandomenico, D., San Pietro, E., Beznoussenko, G.V., Polishchuk, E.V., Baldassarre, M., *et al.* (2004). Secretory traffic triggers the formation of tubular continuities across Golgi sub-compartments. Nat Cell Biol *6*, 1071-1081.
